# Author Correction: Late Paleolithic whale bone tools reveal human and whale ecology in the Bay of Biscay

**DOI:** 10.1038/s41467-025-63273-w

**Published:** 2025-08-21

**Authors:** Krista McGrath, Laura G. van der Sluis, Alexandre Lefebvre, Anne Charpentier, Ana S. L. Rodrigues, Esteban Álvarez-Fernández, François Baleux, Eduardo Berganza, François-Xavier Chauvière, Morgane Dachary, Elsa Duarte Matías, Claire Houmard, Ana B. Marín-Arroyo, Marco de la Rasilla Vives, Jesus Tapia, François Thil, Olivier Tombret, Leire Torres-Iglesias, Camilla Speller, Antoine Zazzo, Jean-Marc Pétillon

**Affiliations:** 1 Department of Prehistory and Institute of Environmental Science and Technology (ICTA-UAB), Universitat Autònoma de Barcelona, Bellaterra, Spain; 2 BioArchéologie, Interactions Sociétés Environnements (BioArch), UMR 7209, Muséum national d’Histoire naturelle, CNRS, Paris, France; 3 Department of Evolutionary Anthropology, University of Vienna, Vienna, Austria; 4 Human Evolution and Archaeological Sciences, Vienna, Austria; 5 Grupo I + D + i EVOADAPTA, Universidad de Cantabria, Santander, Spain; 6 De la Préhistoire à l’Actuel: Culture, Environnement et Anthropologie (PACEA), UMR 5199, CNRS, Université de Bordeaux, Pessac, France; 7 CEFE, Univ Montpellier, CNRS, EPHE, IRD, Montpellier, France; 8 GIR PREHUSAL, Universidad de Salamanca, Facultad de Geografía e Historia, Departamento de Prehistoria, Historia Antigua y Arqueología, Salamanca, Spain; 9 Travaux et Recherches Archéologiques sur les Cultures, les Espaces et les Sociétés (TRACES) UMR 5608, CNRS, Université Toulouse Jean Jaurès, Toulouse, France; 10 Sociedad de Ciencias Aranzadi, Donostia-San Sebastian, Spain; 11 Office du patrimoine et de l’archéologie du canton de Neuchâtel, section Archéologie, Laténium, Hauterive, Switzerland; 12 Ministère de la Culture, Service Régional de l’Archéologie de Nouvelle-Aquitaine, Limoges, France; 13 Departamento de Historia, Universidad de Oviedo, Oviedo, Spain; 14 Université de Franche-Comté, UMR 6249 Chrono-environnement, Besançon, France; 15 Laboratoire des Sciences du Climat et de l’Environnement (LSCE/IPSL), UMR 8212, CEA-CNRS-UVSQ, Université Paris Saclay, Gif-sur-Yvette, France; 16 Section for Molecular Ecology and Evolution, Globe Institute, University of Copenhagen, Øster Farimagsgade 5, Copenhagen, Denmark; 17 Department of Anthropology, University of British Columbia, Vancouver, Canada

**Keywords:** Palaeoecology, Biodiversity, Archaeology, Archaeology, Marine mammals

Correction to: *Nature Communications* 10.1038/s41467-025-59486-8, published online 27 May 2025

In the version of the article initially published, due to a version error, the image shown in Fig. 2 did not include an inset map shown in the “16-14.5 ka cal BP” panel indicating region 22, as is now updated in the HTML and PDF versions of the article.

