# Supplementary material for: Late Paleolithic whale bone tools reveal human and whale ecology in the Bay of Biscay
Source: Nat Commun. 2025 May 27;16:4646. doi: 10.1038/s41467-025-59486-8 (PMC12117114; doi:10.1038/s41467-025-59486-8)
Supplement: Supplementary file 1 — Supplementary Information [file 41467_2025_59486_MOESM1_ESM.pdf]

Supplementary Information for
"Late Paleolithic whale bone tools reveal human and whale ecology in the Bay of Biscay"

Krista McGrath[1α], Laura G. van der Sluis[2,3α], Alexandre Lefebvre[4,5], Anne Charpentier[6], Ana Rodrigues[6], Esteban Álvarez-Fernández[7], François Baleux[8], Eduardo Berganza[9], François-Xavier Chauvière[10], Morgane Dachary[8,11], Elsa Duarte Matías[12], Claire Houmard[13], Ana B. Marín-Arroyo[4], Marco de la Rasilla Vives[12], Jesus Tapia[9], François Thil[14], Olivier Tombret[2], Leire Torres-Iglesias[4], Camilla Speller[15], Antoine Zazzo[2] & Jean-Marc Pétillon[8]*

[α] These authors contributed equally.
* Corresponding author: jean-marc.petillon@cnrs.fr

[1]Department of Prehistory and Institute of Environmental Science and Technology (ICTA), Universidad Autònoma de Barcelona, Bellaterra, Spain. [2]BioArchéologie, Interactions Sociétés Environnements (BioArch), UMR 7209, Muséum national d'Histoire naturelle, CNRS, Paris, France. [3]Department of Evolutionary Anthropology, University of Vienna, Vienna, Austria. Human Evolution and Archaeological Sciences (HEAS), Vienna, Austria. [4]Grupo I+D+i EVOADAPTA, Universidad de Cantabria, Santander, Spain. [5]De la Préhistoire à l'Actuel : Culture, Environnement et Anthropologie (PACEA), UMR 5199, CNRS, Université de Bordeaux, Pessac, France. [6]CEFE, Univ Montpellier, CNRS, EPHE, IRD, Montpellier, France. [7]GIR PREHUSAL, Universidad de Salamanca, Facultad de Geografía e Historia, Departamento de Prehistoria, Historia Antigua y Arqueología, Salamanca, Spain. [8]Travaux et Recherches Archéologiques sur les Cultures, les Espaces et les Sociétés (TRACES) UMR 5608, CNRS, Université Toulouse Jean Jaurès, Toulouse, France. [9]Sociedad de Ciencias Aranzadi, Donostia-San Sebastian, Spain. [10]Office du patrimoine et de l'archéologie du canton de Neuchâtel, section Archéologie, Laténium, Hauterive, Switzerland. [11]Ministère de la Culture, Service Régional de l'Archéologie de Nouvelle-Aquitaine, Limoges, France. [12]Departamento de Historia, Universidad de Oviedo, Oviedo, Spain. [13]Université de Franche-Comté, UMR 6249 Chrono-environnement, Besançon, France. [14]Laboratoire des Sciences du Climat et de l'Environnement (LSCE/IPSL), UMR 8212, CEA-CNRS-UVSQ, Université Paris Saclay, Gif-sur-Yvette, France. [15]Department of Anthropology, University of British Columbia, Vancouver, Canada.

This pdf file includes:
- Supplementary Figures 1 through 102.
- Supplementary Discussion: Comparing stable isotope signatures in Paleolithic versus modern whales
- Supplementary References: references cited in the captions of Supplementary Figures 99 through 102, or in the Supplementary Discussion.

Supplementary Figures 1 through 90: Catalogue.

This catalogue includes the pictures of:

- All the worked objects whose identification as whale bone was confirmed by ZooMS, except: study numbers 590, 625 and 629, whose pictures could not be taken at the museum; study numbers 993, 994 and AN2 25/83-73 N17, published in other articles (Langley and Street 2013; Lucas et al. 2023).

- A sample of 26 unworked whale bone fragments from Santa Catalina, with a priority given to those that have an individual excavation number (as opposed to those found during screening, identified only through their spit number), and that were both analyzed with ZooMS and radiocarbon-dated.

Unless otherwise stated, the scale is 1:1, and the pictures were taken before sampling.

All pictures by A. Lefebvre and J.-M. Pétillon.

Supplementary Figure 1
Study number: 2
Museum number: MAN 77159I9, I E omega 1920
Site and layer: Isturitz, salle de Saint-Martin, SI/E omega
Typology: projectile point
ZooMS ID: Cetacean + cow glue contamination
^{14}C date: none

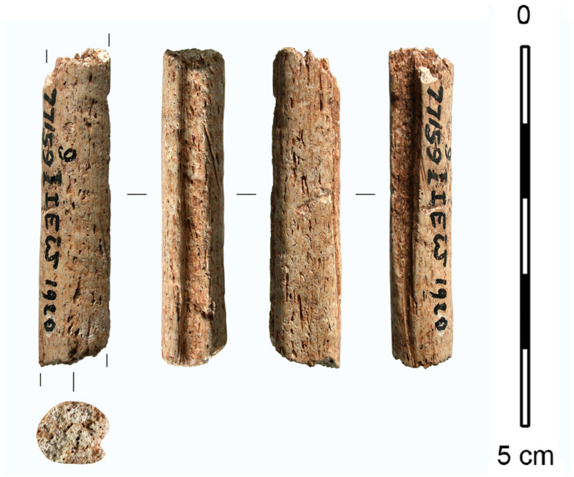

Supplementary Figure 2
Study number: 4
Museum number: MAN 77159I6, I E St alpha 1914
Site and layer: Isturitz, Grande Salle, II/E
Typology: projectile point
ZooMS ID: Sperm whale
^{14}C date: MUSE21043, 14260 ± 60 BP, 17268-16703 cal BP

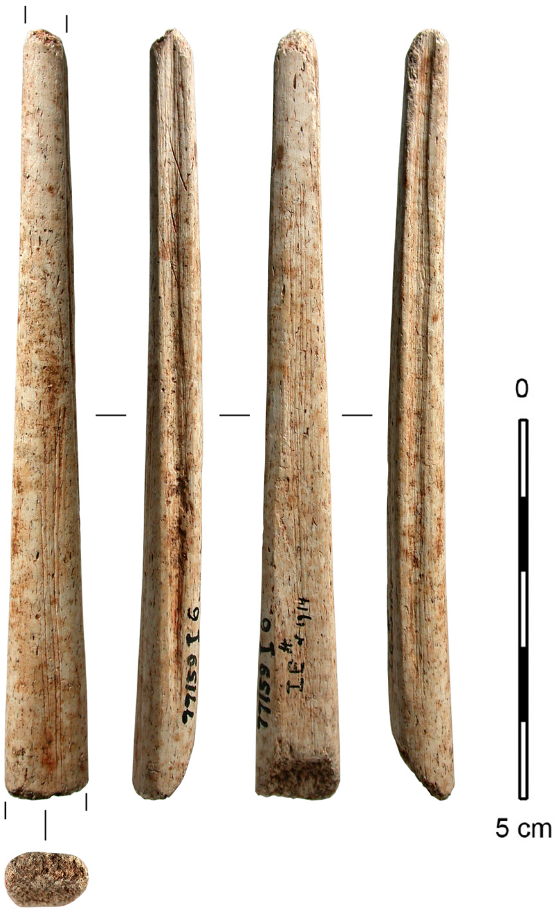

Supplementary Figure 3
Study number: 6
Museum number: MAN Ist I (S) 1928, C2RMF: A
Site and layer: Isturitz, salle de Saint-Martin, SI/E omega
Typology: projectile point with massive base
ZooMS ID: Right whale / Bowhead whale
^{14}C date: MUSE21044, 14290 ± 60 BP, 17299-16746 cal BP

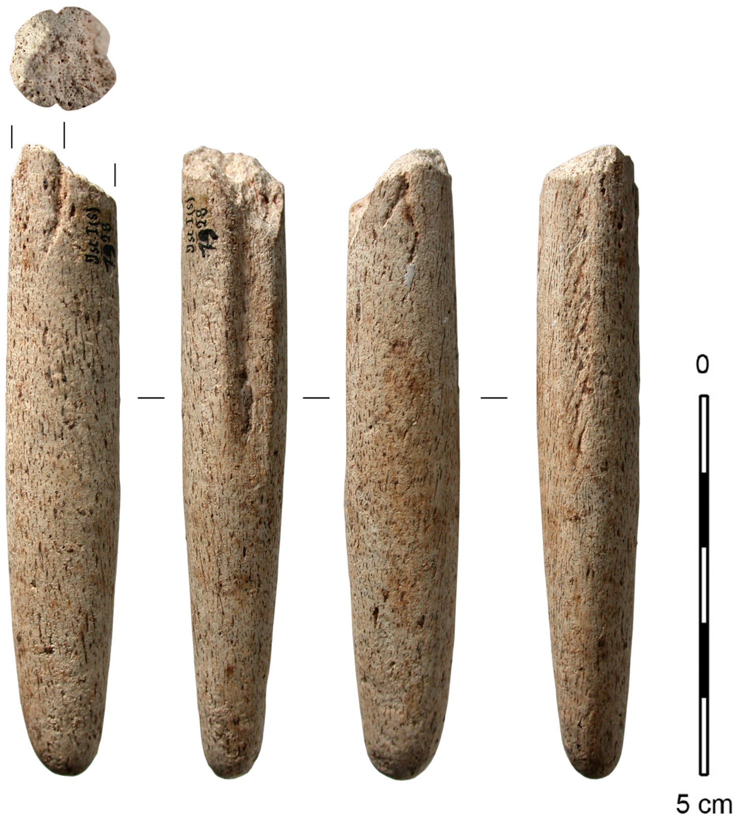

Supplementary Figure 4
Study number: 7
Museum number: MAN Ist SI 1929, C2RMF: K
Site and layer: Isturitz, salle de Saint-Martin, SI/E omega
Typology: wedge
ZooMS ID: Cetacean + cow glue contamination
^{14}C date: none

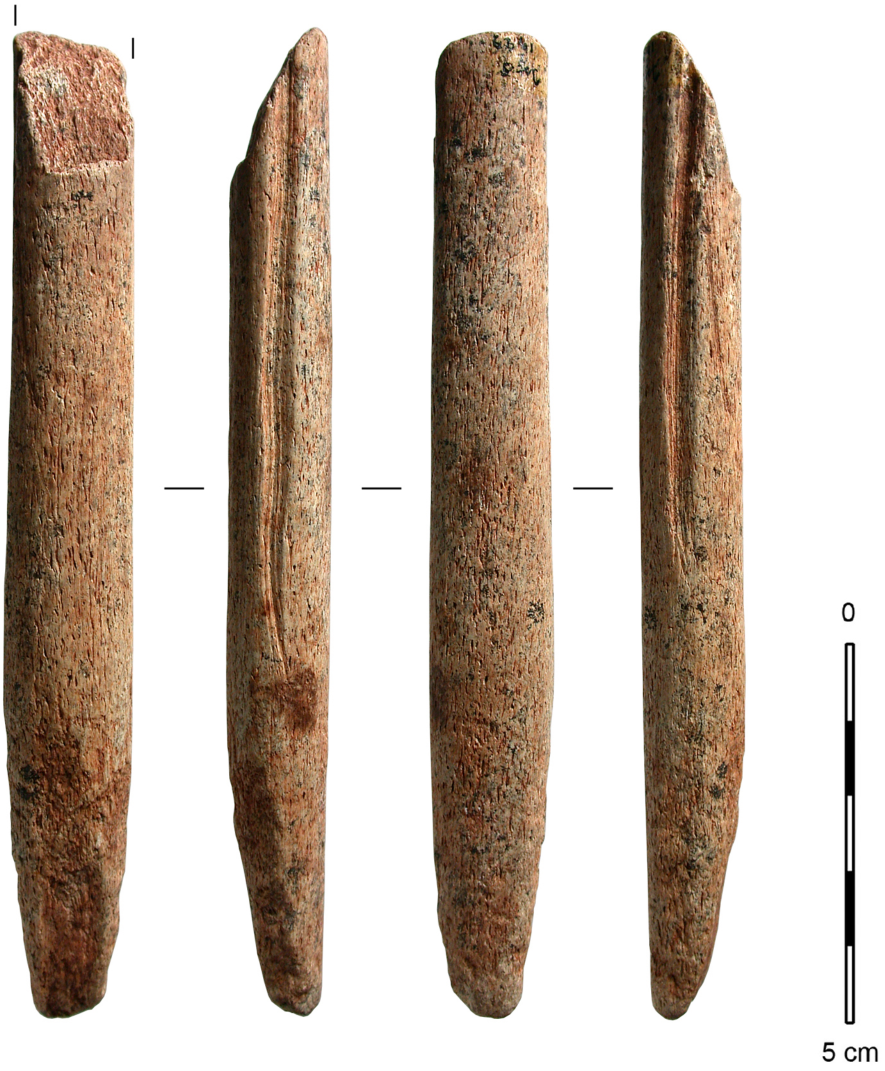

Supplementary Figure 5
Study number: 10
Museum number: MAN Ist II 1931
Site and layer: Isturitz, Grande Salle, II/E
Typology: projectile point
ZooMS ID: Sperm whale + cow glue contamination
^{14}C date: none

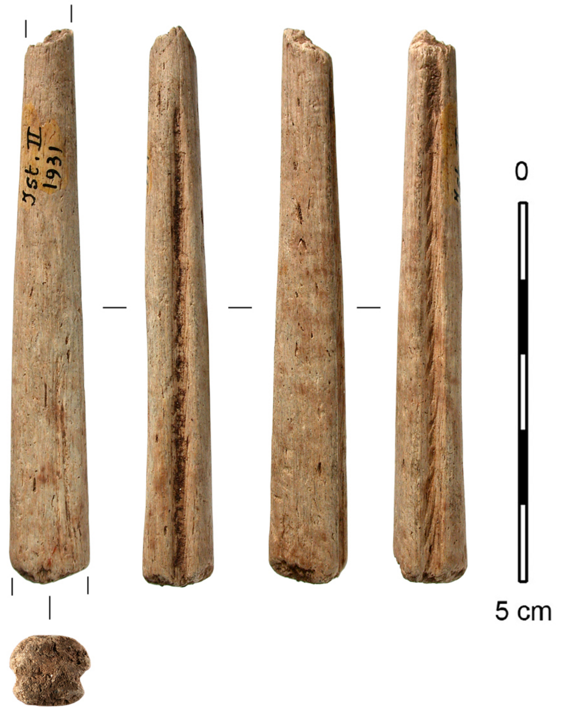

Supplementary Figure 6
Study number: 13
Museum number: MAN 77158A121
Site and layer: Isturitz, Grande Salle, II/E?
Typology: projectile point with massive base
ZooMS ID: Sperm whale
^{14}C date: MUSE21045, 15110 ± 70 BP, 18248-17742 cal BP

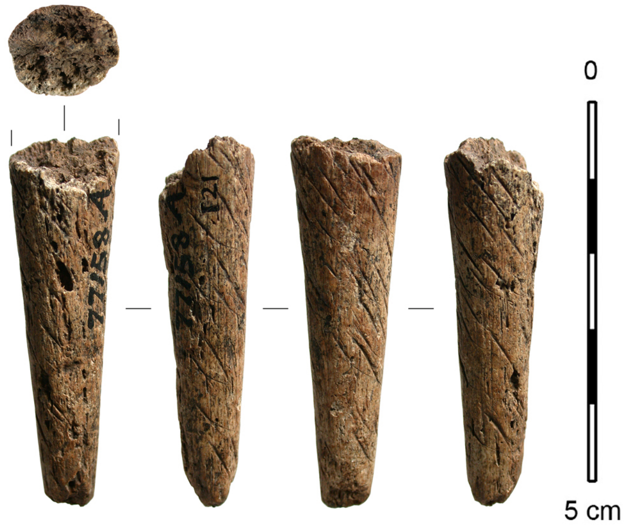

Supplementary Figure 7
Study number: 14
Museum number: MAN Ist II 1932
Site and layer: Isturitz, Grande Salle, II/E
Typology: projectile point with massive base
ZooMS ID: Sperm whale + cow glue contamination
^{14}C date: none

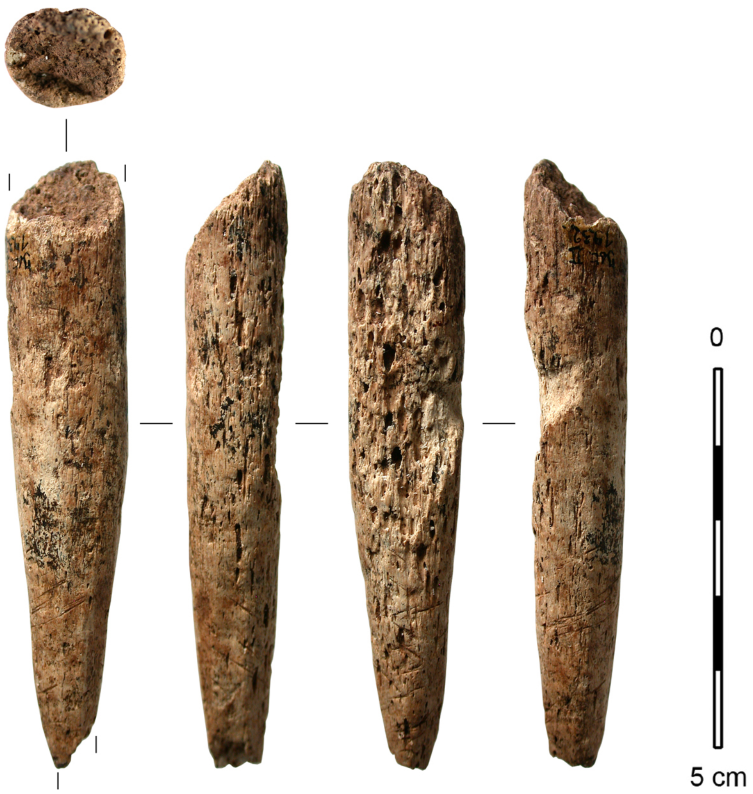

Supplementary Figure 8
Study number: 15
Museum number: MAN 77153A5, IE alpha 1914
Site and layer: Isturitz, Grande Salle, II/E
Typology: projectile point with massive base
ZooMS ID: Blue whale
^{14}C date: MUSE21046, 15530 ± 80 BP, 18718-18198 cal BP

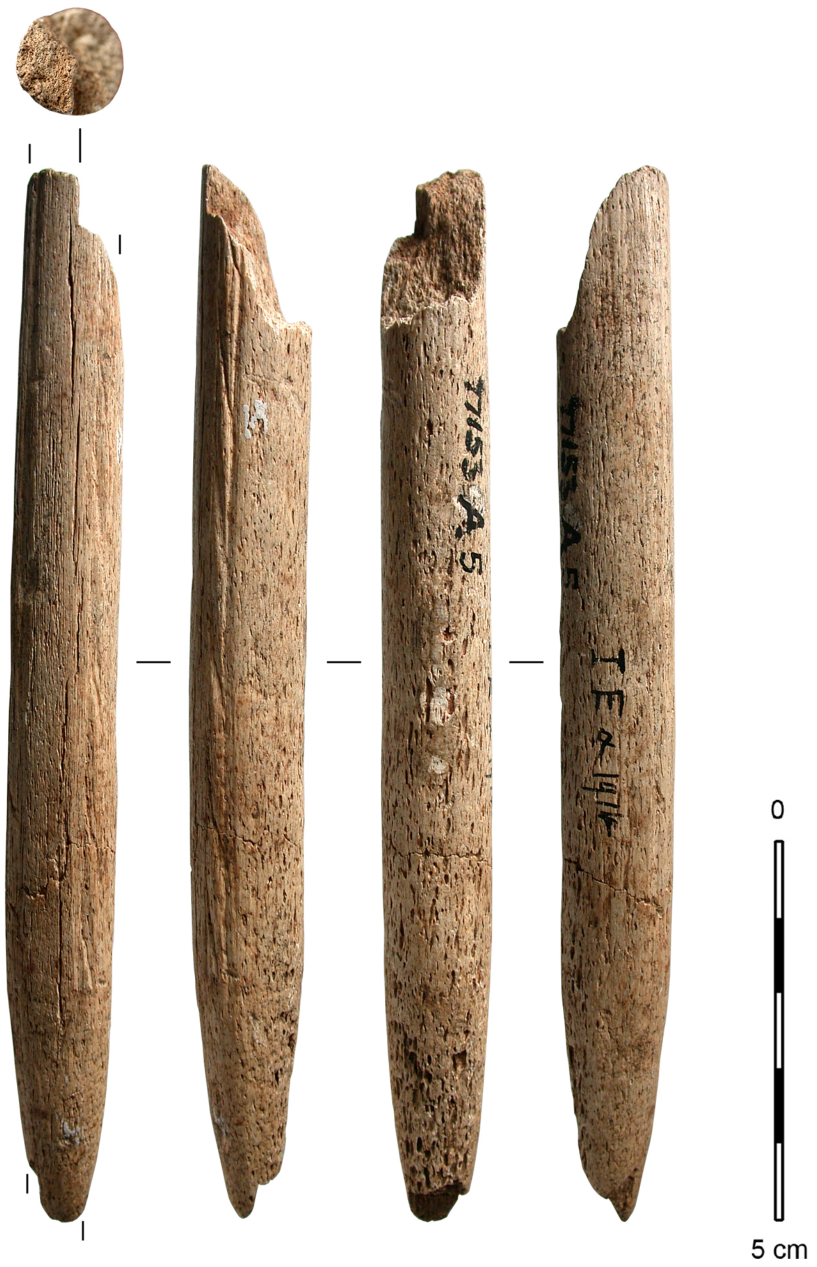

Supplementary Figure 9
Study number: 19
Museum number: MAN Ist SI 1929, C2RMF: J
Site and layer: Isturitz, salle de Saint-Martin, SI/E omega
Typology: object on splinter
ZooMS ID: Cetacean + cow glue contamination
^{14}C date: none

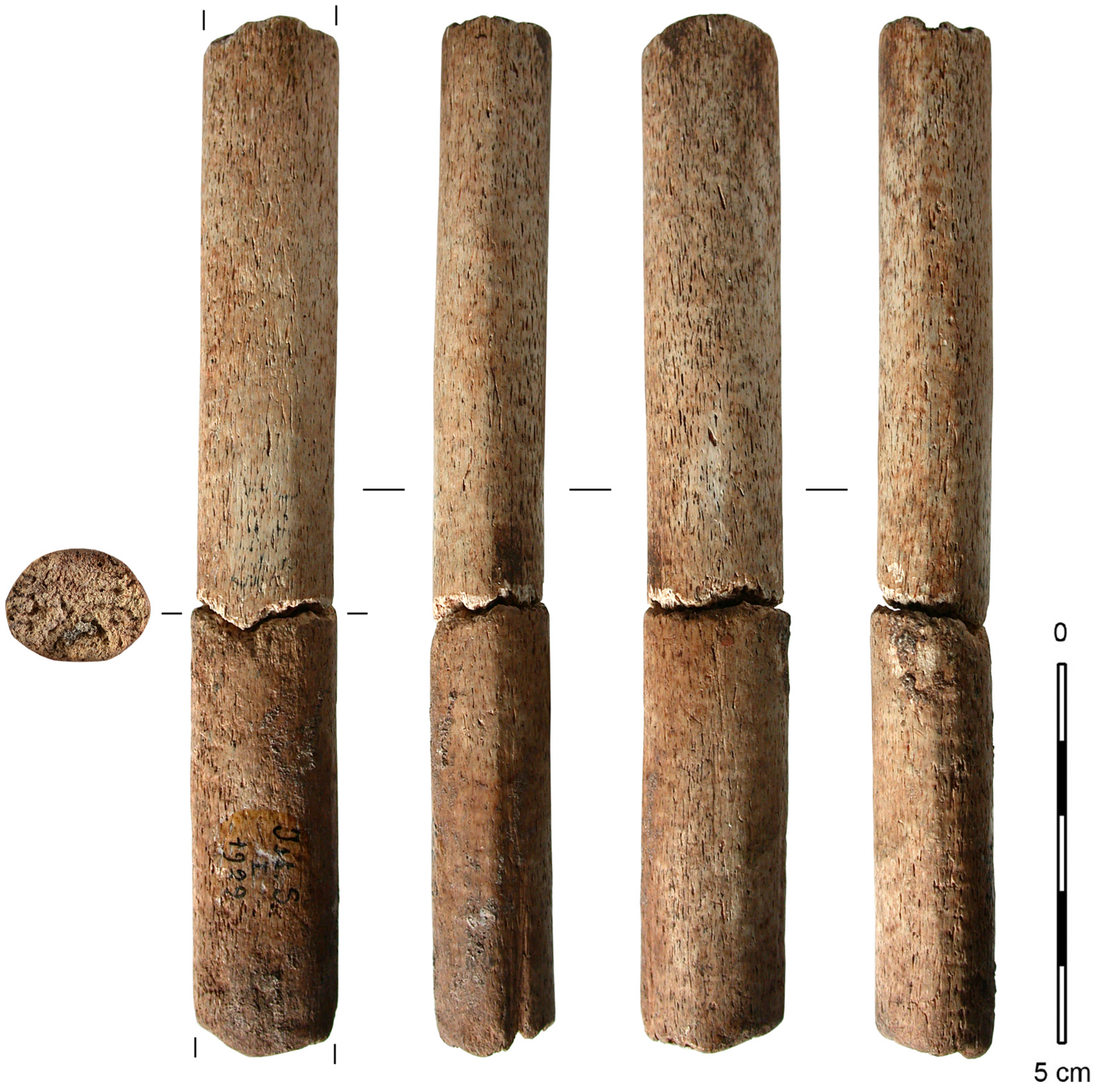

Supplementary Figure 10
Study number: 36
Museum number: MAN 77158B132
Site and layer: Isturitz, Grande Salle, II/E?
Typology: projectile point
ZooMS ID: Right whale / Bowhead whale
^{14}C date: MUSE21047, 14250 ± 60 BP, 17256-16688 cal BP

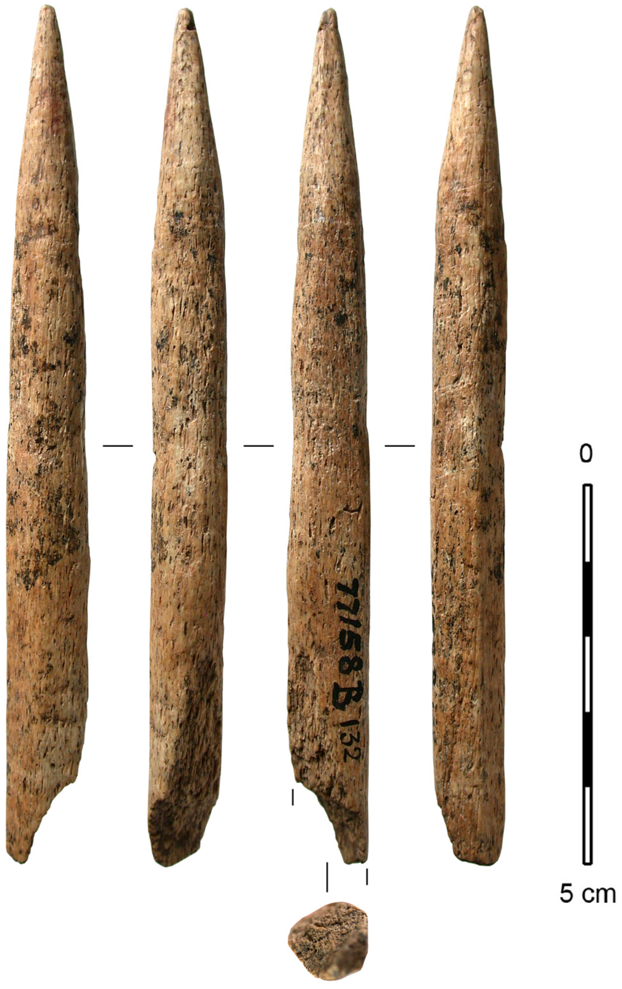

Supplementary Figure 11
Study number: 38
Museum number: MAN Ist II 1934
Site and layer: Isturitz, Grande Salle, II/E
Typology: projectile point with massive base
ZooMS ID: Sperm whale + cow glue contamination
^{14}C date: none

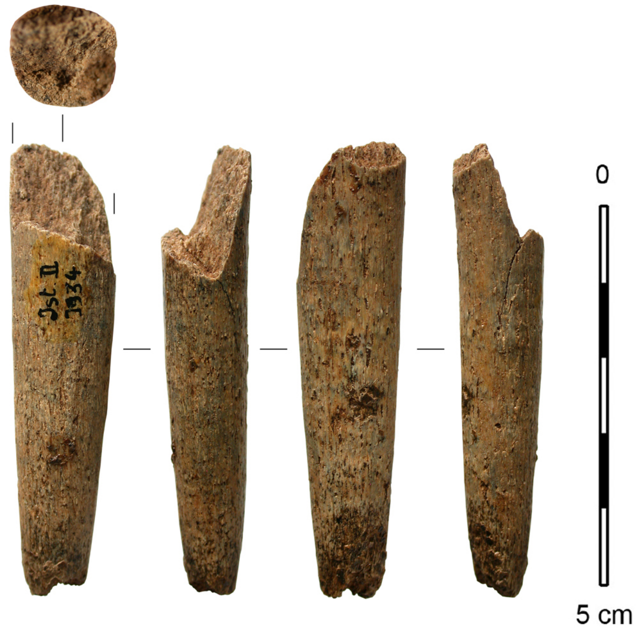

Supplementary Figure 12
Study number: 39
Museum number: MAN Ist II 1937
Site and layer: Isturitz, Grande Salle, II/E
Typology: projectile point with massive base
ZooMS ID: Fin whale + cow glue contamination
^{14}C date: none

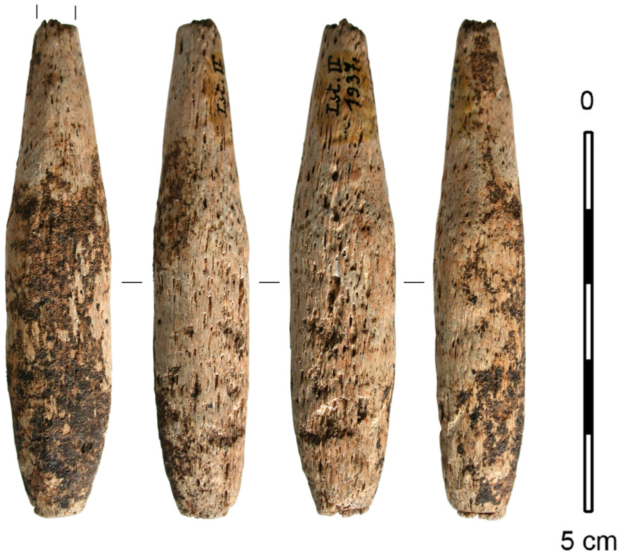

Supplementary Figure 13
Study number: 58
Museum number: Ist GD 2007 déblais paroi est, A
Site and layer: Isturitz, salle de Saint-Martin, GD C2
Typology: projectile point
ZooMS ID: Gray whale
^{14}C date: MUSE21048, failed

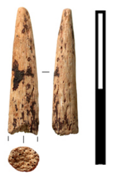

Supplementary Figure 14
Study number: 59
Museum number: Ist GD 2008 B4, B
Site and layer: Isturitz, salle de Saint-Martin, GD C2
Typology: projectile point
ZooMS ID: Fin whale
^{14}C date: MUSE21049, failed

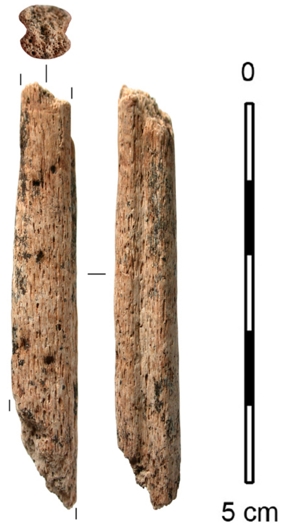

Supplementary Figure 15
Study number: 60
Museum number: Ist GD 2009 B3
Site and layer: Isturitz, salle de Saint-Martin, GD C2
Typology: projectile point
ZooMS ID: Cetacean (Sperm whale?)
^{14}C date: MUSE21050, failed

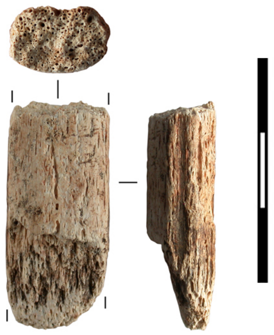

Supplementary Figure 16
Study number: 82
Museum number: Ist GD 2010 B5
Site and layer: Isturitz, salle de Saint-Martin, GD C2
Typology: projectile point
ZooMS ID: Sperm whale
^{14}C date: MUSE21051, failed

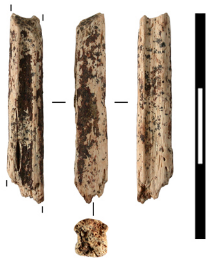

Supplementary Figure 17
Study number: 589
Museum number: MAN 77163G113 + 77162E20, IF1 (on fgt 77162E20)
Site and layer: Isturitz, Grande Salle, I/F1
Typology: foreshaft
ZooMS ID: Fin whale
^{14}C date: MUSE 21058, 13820 ± 60 BP, 16729-16144 cal BP

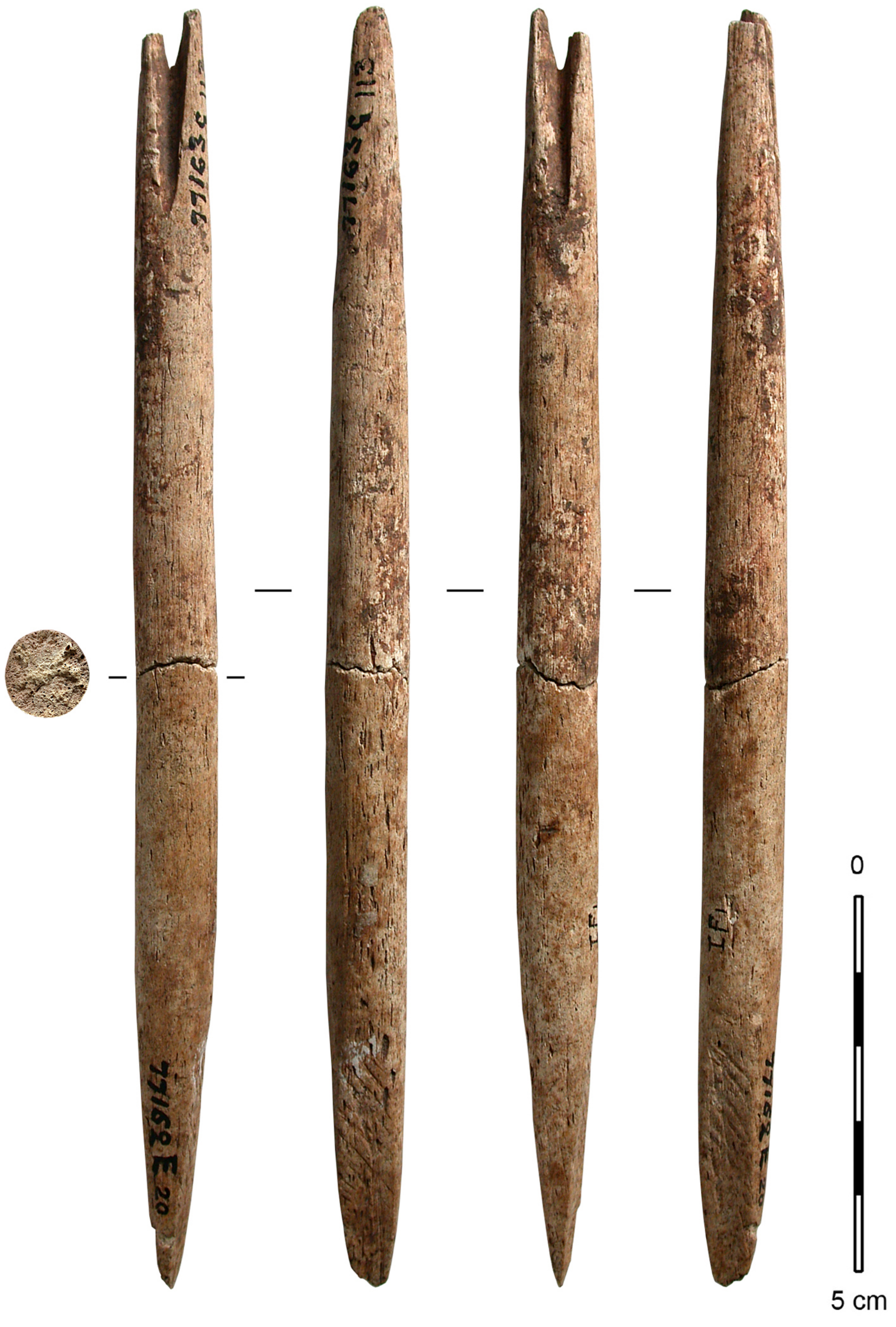

Supplementary Figure 18
Study number: 630
Museum number: MAN Ist II 1931 + Ist II 1931
Site and layer: Isturitz, Grande Salle, II/E
Typology: foreshaft
ZooMS ID: Sperm whale + cow glue contamination
^{14}C date: none
(scale 1:2)

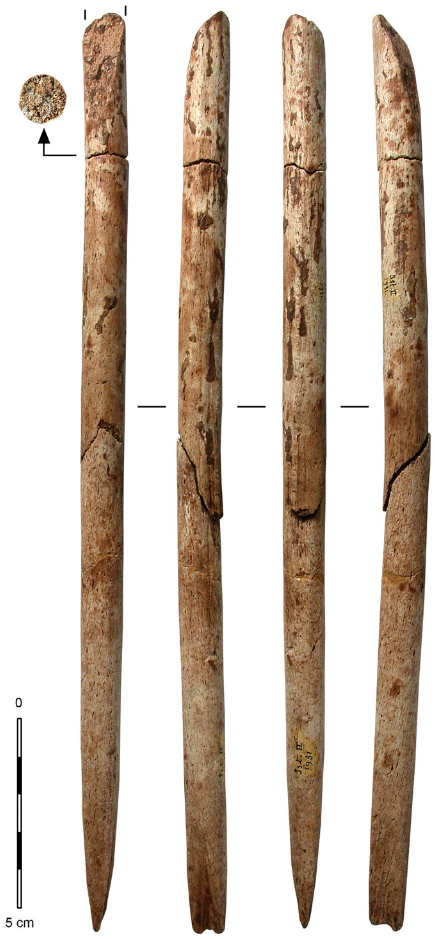

Supplementary Figure 19
Study number: 634
Museum number: MAN 77158B362 + 77158B18
Site and layer: Isturitz, Grande Salle, II/E?
Typology: foreshaft
ZooMS ID: Sperm whale + cow glue contamination
^{14}C date: none

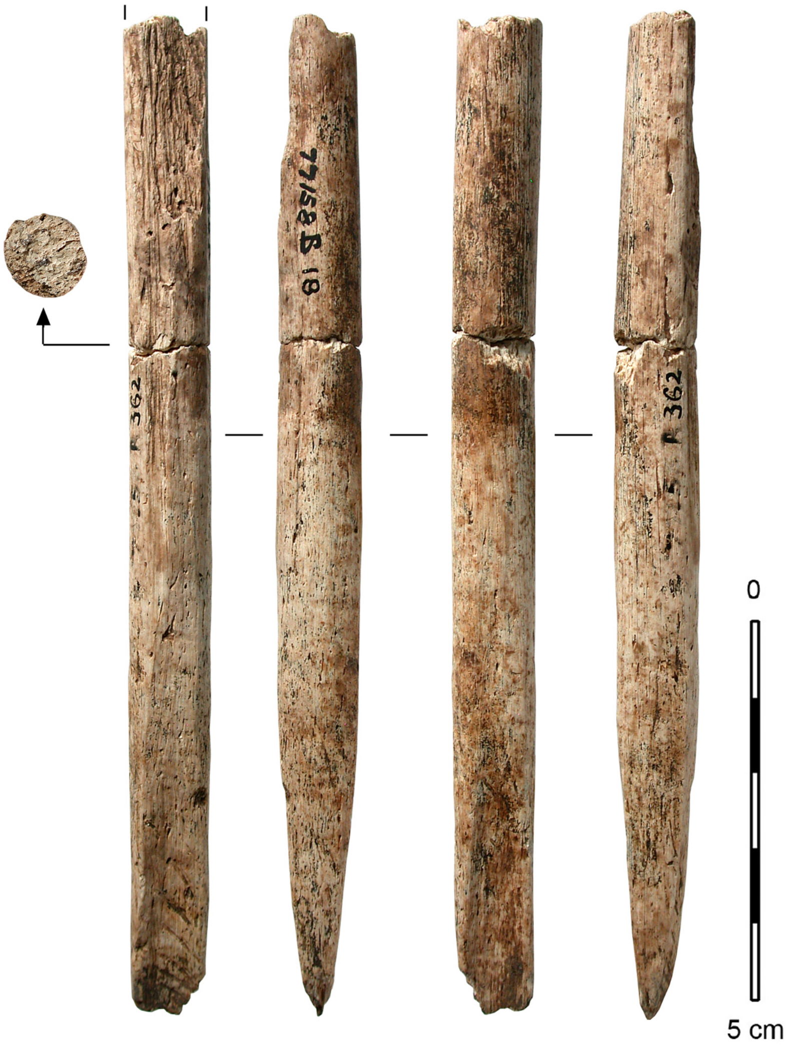

Supplementary Figure 20
Study number: 637
Museum number: MAN 77159D10, IE 1914
Site and layer: Isturitz, Grande Salle, II/E
Typology: foreshaft
ZooMS ID: Sperm whale + cow glue contamination
^{14}C date: none

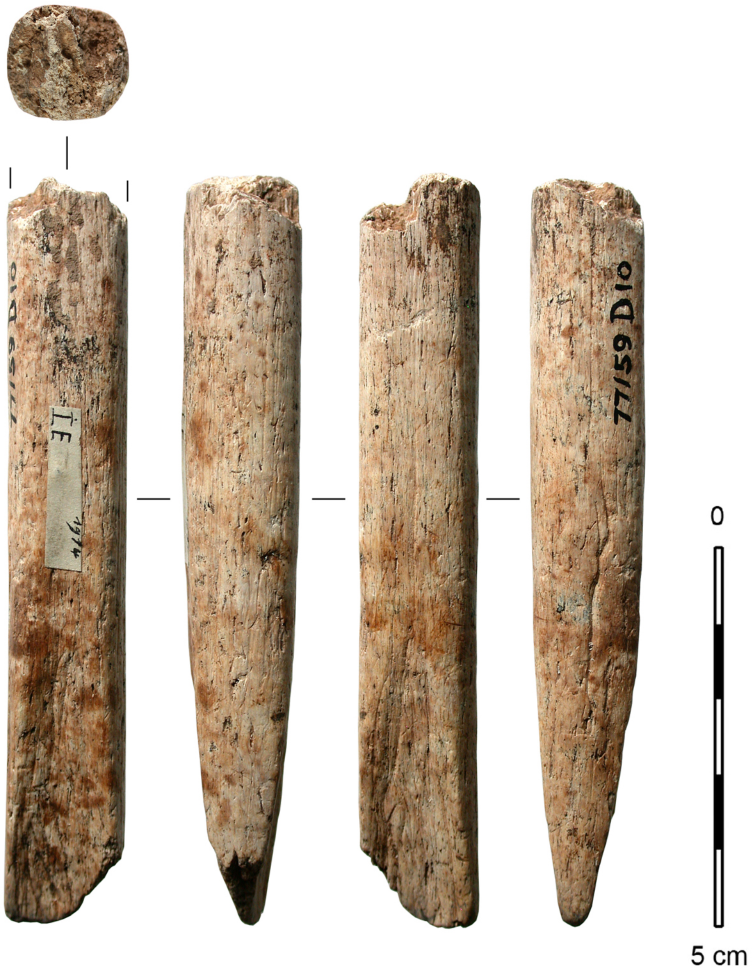

Supplementary Figure 21
Study number: 749
Museum number: MAN Ist II 1930 + Ist I 1930
Site and layer: Isturitz, Grande Salle, I/F1 + II/E
Typology: object on splinter
ZooMS ID: Fin whale + cow glue contamination
^{14}C date: none

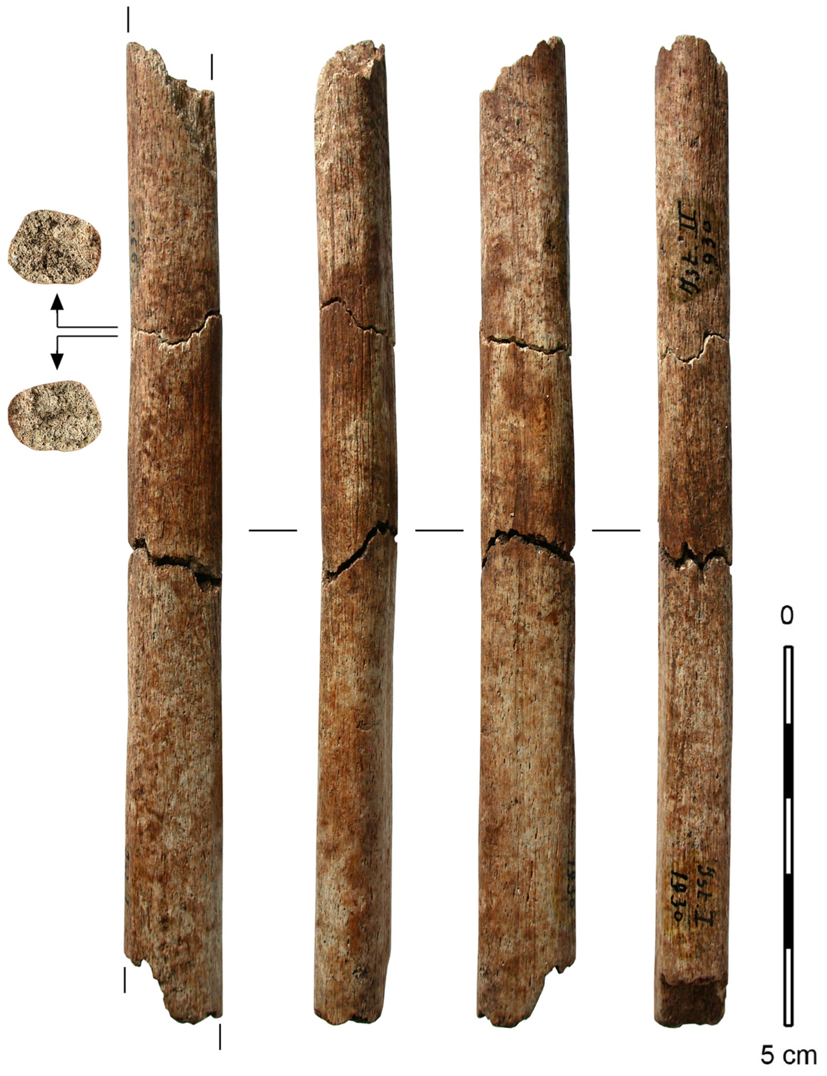

Supplementary Figure 22
Study number: 780
Museum number: MAN Ist I 1930
Site and layer: Isturitz, Grande Salle, I/F1
Typology: wedge
ZooMS ID: Sperm whale + cow glue contamination
^{14}C date: none

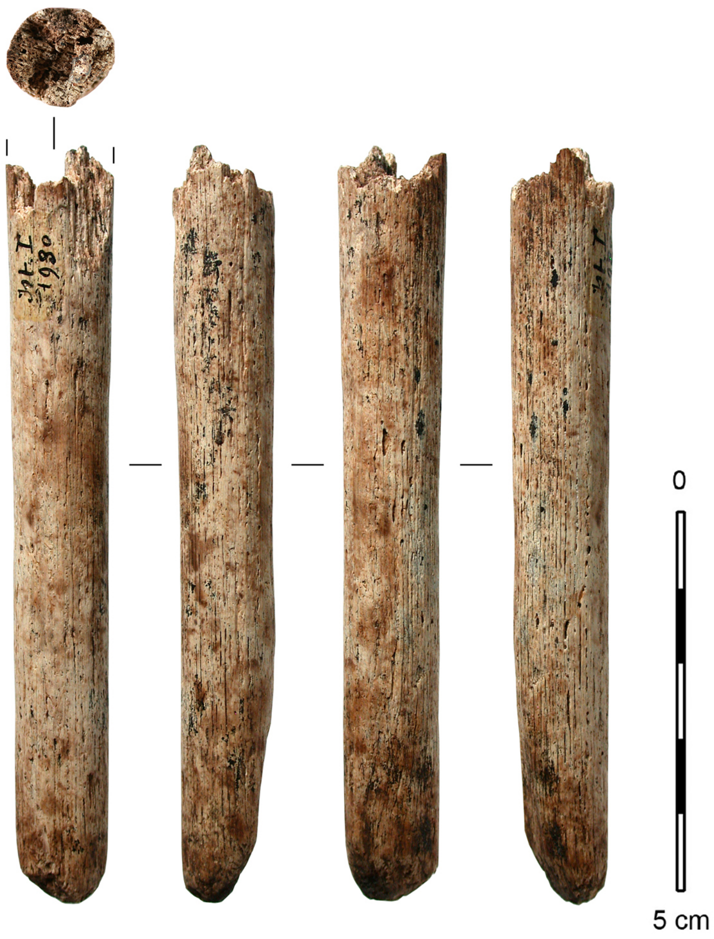

Supplementary Figure 23
Study number: 781
Museum number: MHNT
Site and layer: Isturitz
Typology: projectile point
ZooMS ID: Sperm whale
^{14}C date: MUSE 21054, 14430 ± 60 BP, 17446-16911 cal BP

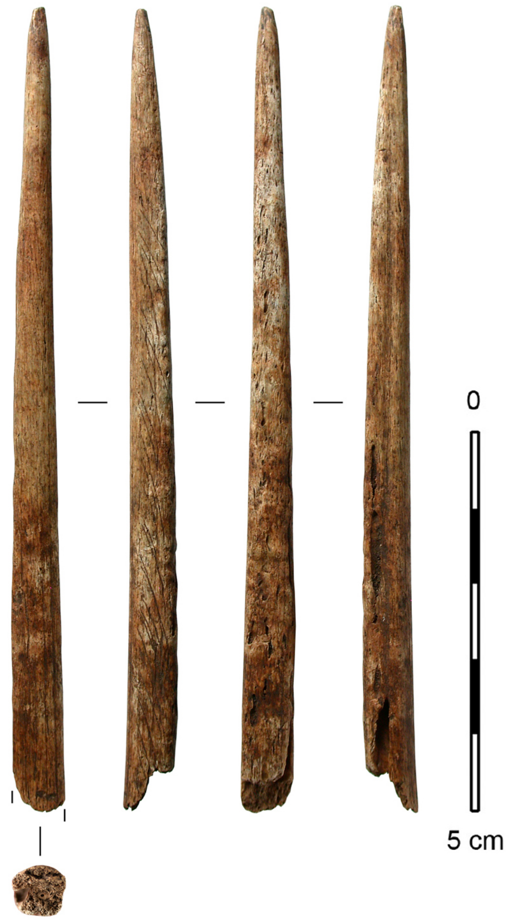

Supplementary Figure 24
Study number: 783
Museum number: MAN 83642 + 83643, F28/29 + K69
Site and layer: Vache 3 & 4
Typology: unidentified
ZooMS ID: Right whale / Bowhead whale
^{14}C date: MUSE20137, 14100 ± 60 BP, 17046-16501 cal BP

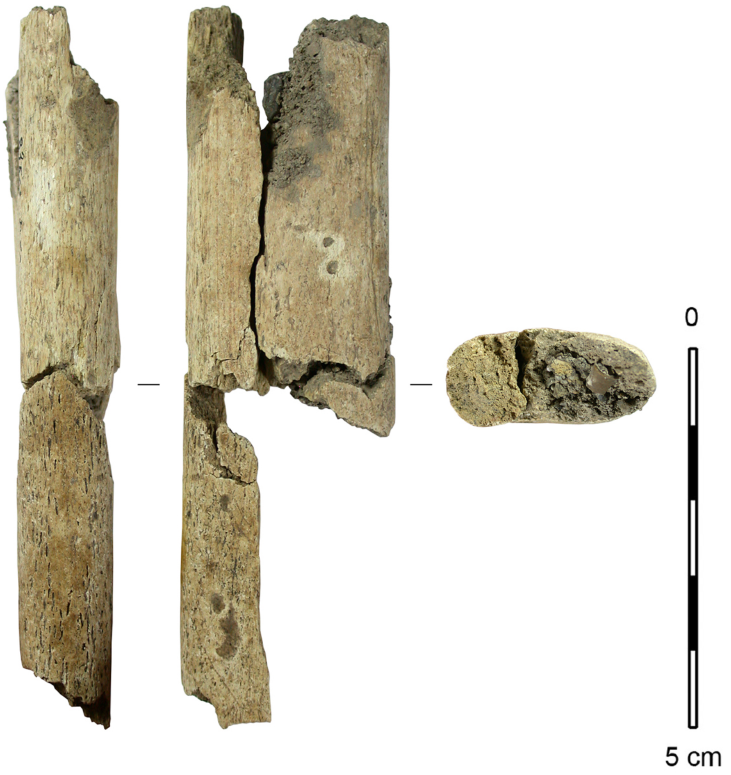

Supplementary Figure 25
Study number: 785
Museum number: MAN 83643, F8
Site and layer: Vache 4
Typology: wedge
ZooMS ID: Right whale / Bowhead whale
^{14}C date: MUSE 20138, 14020 ± 60 BP, 16967-16402 cal BP

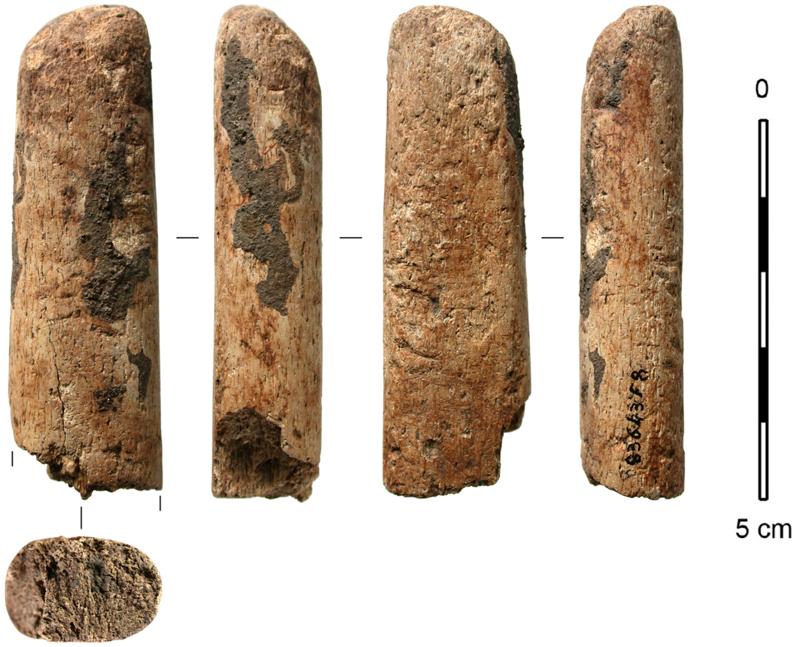

Supplementary Figure 26
Study number: 798
Museum number: MAN 56719, C2RMF: H1
Site and layer: Saint-Michel
Typology: projectile point with massive base
ZooMS ID: Right whale / Bowhead whale
^{14}C date: MUSE20139, 14620 ± 60 BP, 17744-17114 cal BP

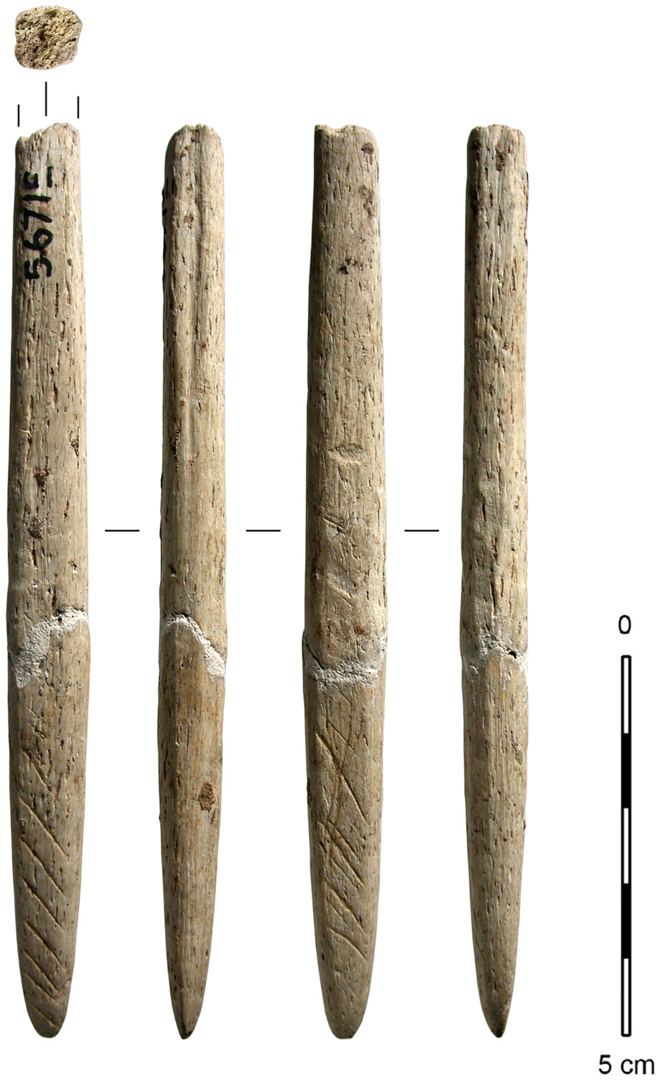

Supplementary Figure 27
Study number: 799
Museum number: MAN 56410, B
Site and layer: Saint-Michel
Typology: unidentified
ZooMS ID: Sperm whale
^{14}C date: MUSE21042, 14450 ± 60 BP, 17477-16930 cal BP

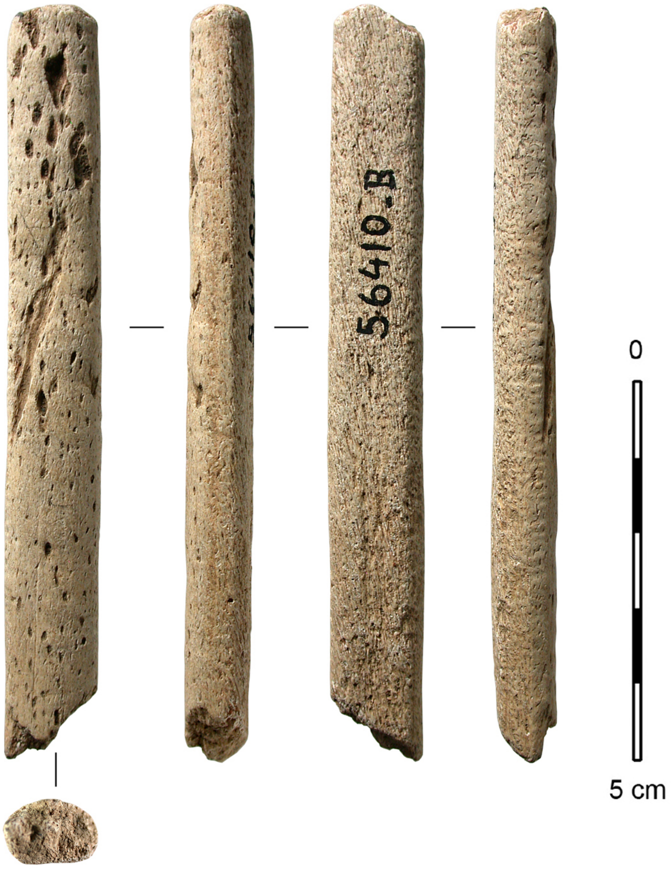

Supplementary Figure 28
Study number: 815
Museum number: MH 55.33.9.1
Site and layer: Tuc d’Audoubert
Typology: foreshaft
ZooMS ID: Sperm whale
^{14}C date: none
(scale 1:2)

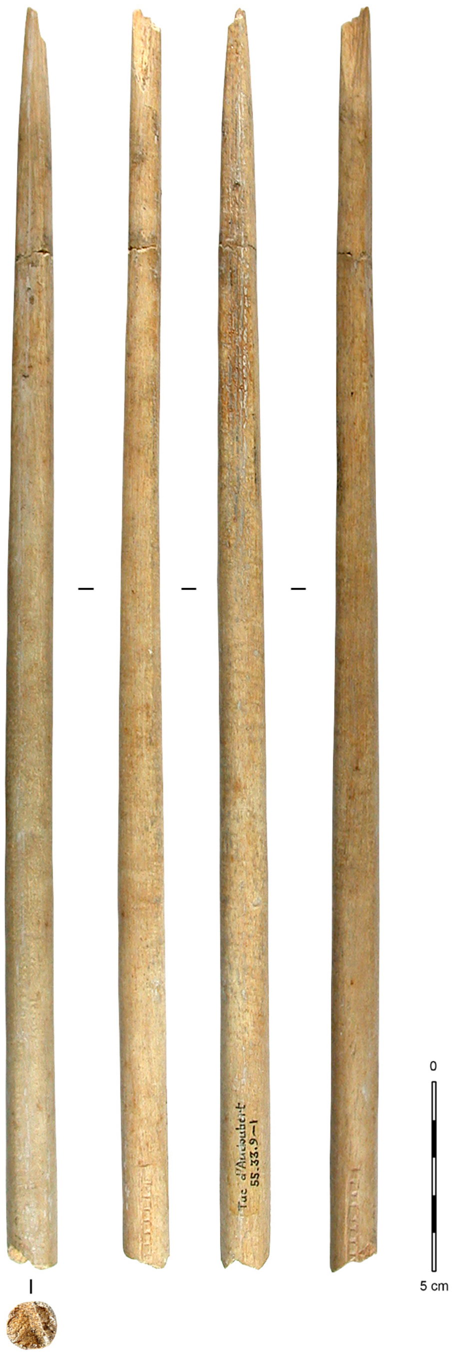

Supplementary Figure 29
Study number: 816
Museum number: MHNT 17
Site and layer: Mas d'Azil
Typology: blank?
ZooMS ID: Sperm whale
^{14}C date: MUSE20131, 14440 ± 90 BP, 17532-16875 cal BP

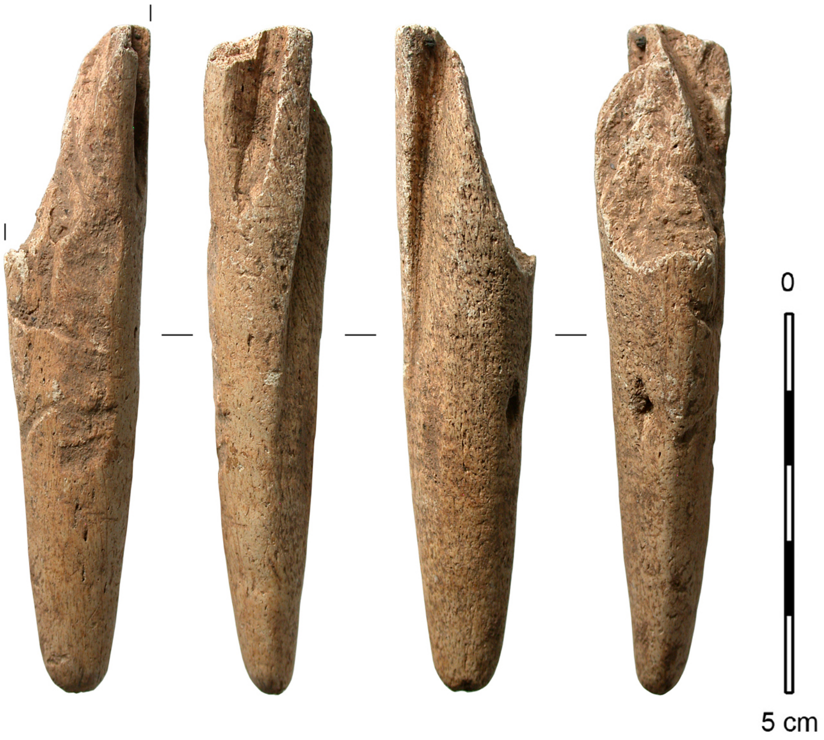

Supplementary Figure 30
Study number: 817
Museum number: MHNT MAZ 99.23.24
Site and layer: Mas d'Azil
Typology: projectile point with massive base
ZooMS ID: Sperm whale
^{14}C date: MUSE21038, 14040 ± 60 BP, 16987-16428 cal BP

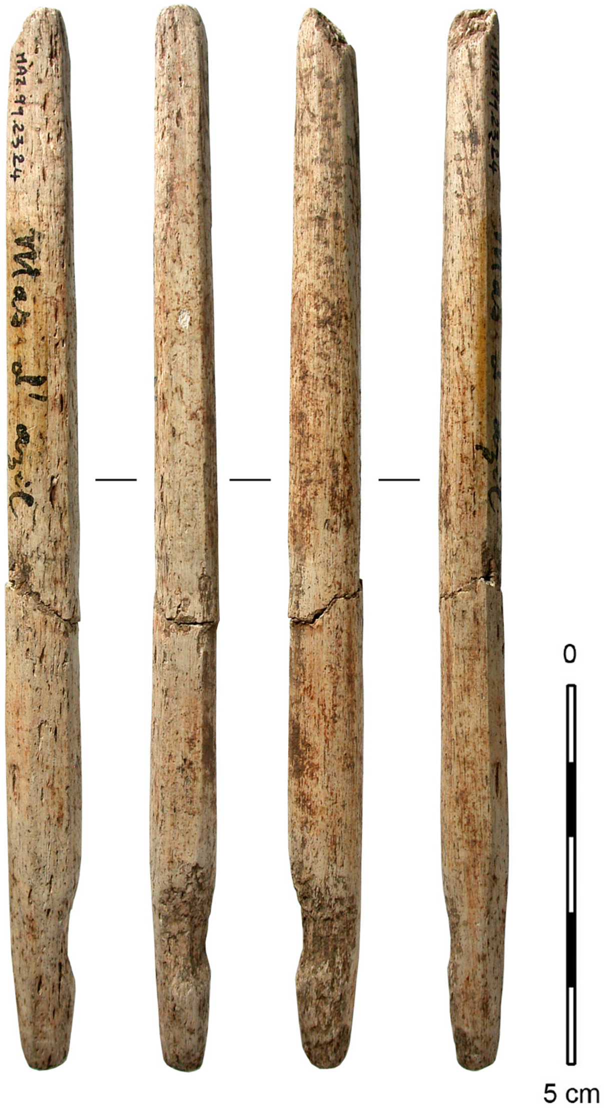

Supplementary Figure 31
Study number: Hum5
Museum number: 3945
Site and layer: Mas d'Azil
Typology: unidentified
ZooMS ID: Sperm whale
^{14}C date: R00215.1, 14240 ± 190 BP, 16388-17462 cal BP

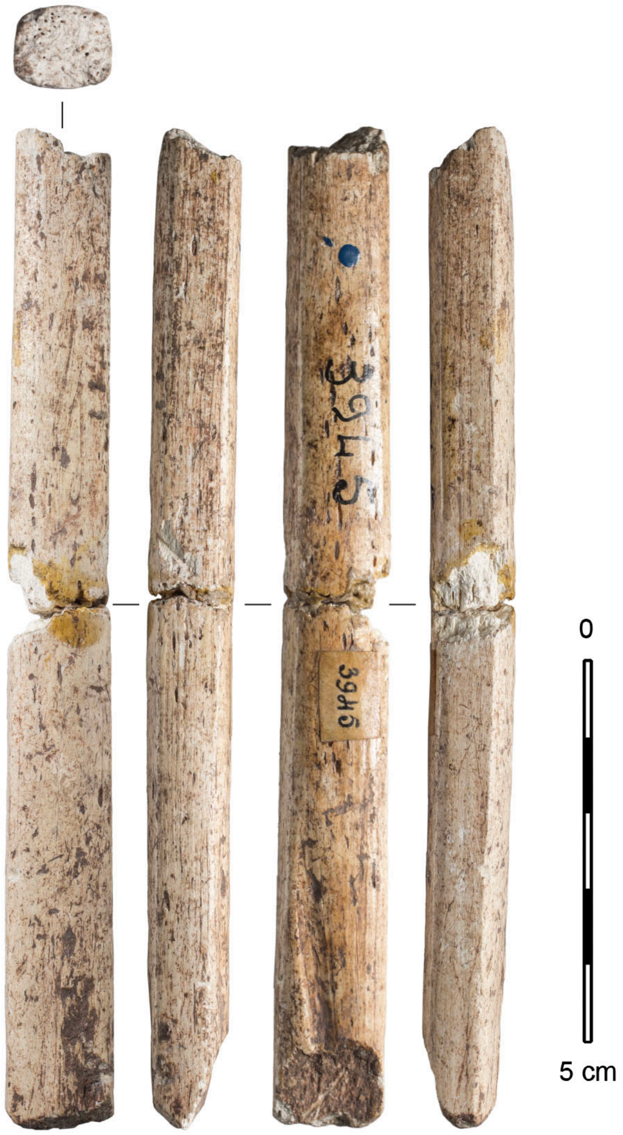

Supplementary Figure 32
Study number: 820
Museum number: MDW Brassempouy, Dubalen collection
Site and layer: Brassempouy, grotte du Pape
Typology: shaping/maintenance waste?
ZooMS ID: Gray whale
^{14}C date: MUSE21039, 13940 ± 60 BP, 16884-16299 cal BP

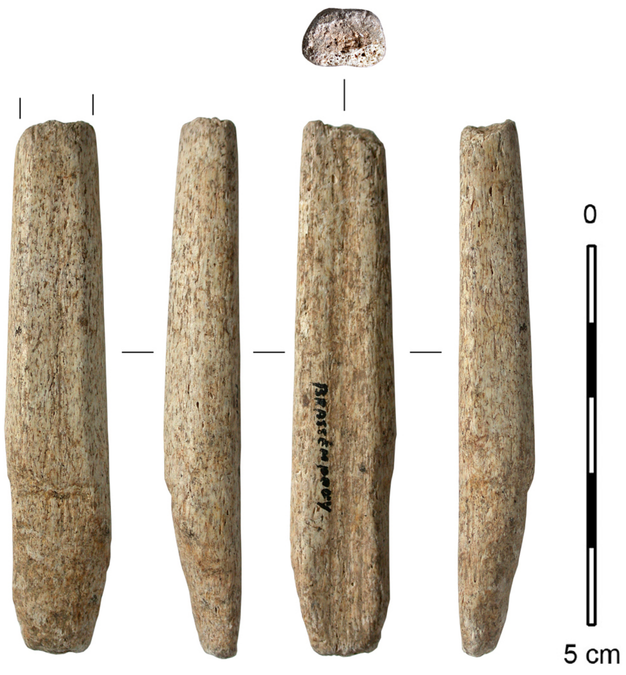

Supplementary Figure 33
Study number: 821
Museum number: MDW Brassempouy, Dubalen collection
Site and layer: Brassempouy, grotte du Pape
Typology: unidentified
ZooMS ID: Sperm whale
^{14}C date: MUSE21040, 13660 ± 60 BP, 16514-15946 cal BP

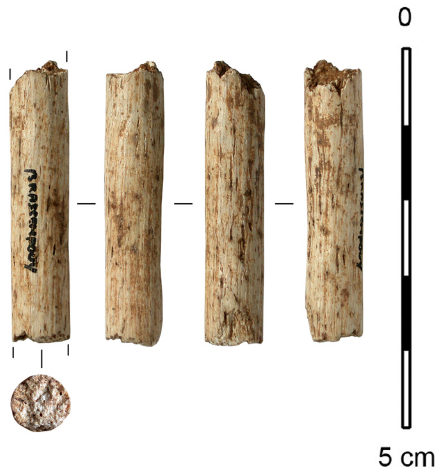

Supplementary Figure 34
Study number: 822
Museum number: MDW Brassempouy, Dubalen collection
Site and layer: Brassempouy, grotte du Pape
Typology: projectile point
ZooMS ID: Fin whale
^{14}C date: MUSE21041, 14150 ± 70 BP, 17119-16536 cal BP

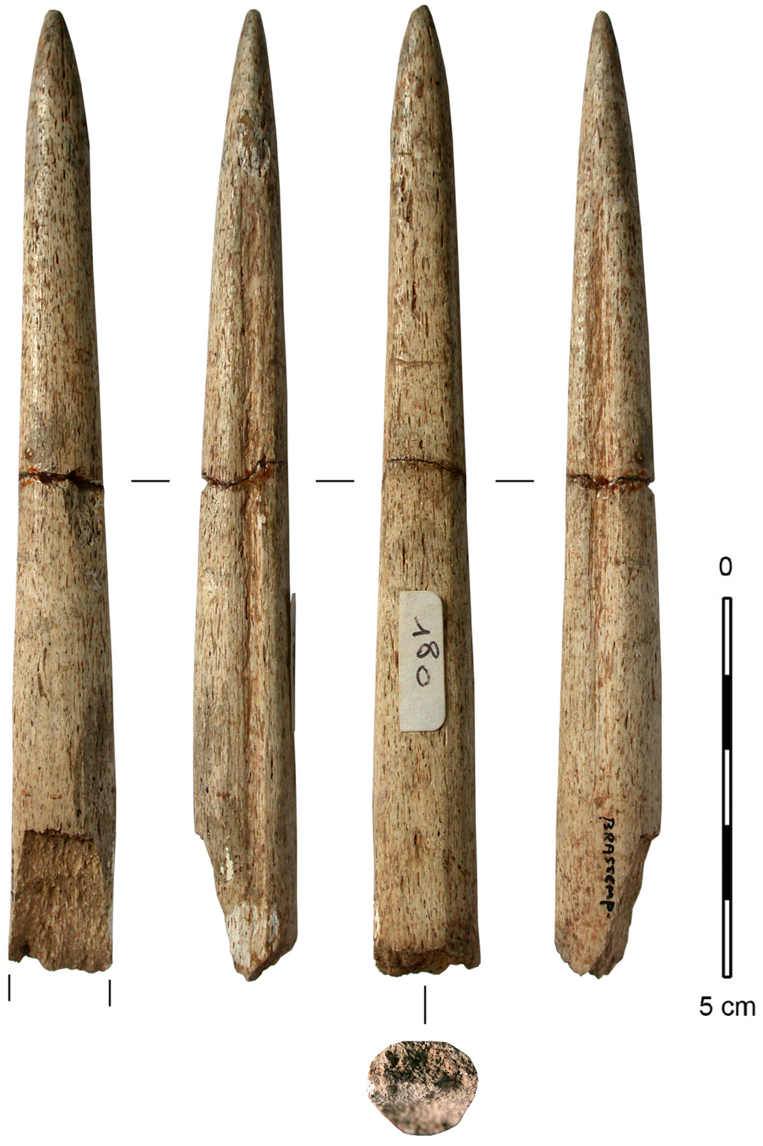

Supplementary Figure 35
Study number: 827
Museum number: MNP
Site and layer: Madeleine, Magd. V
Typology: projectile point with massive base
ZooMS ID: Gray whale
^{14}C date: none

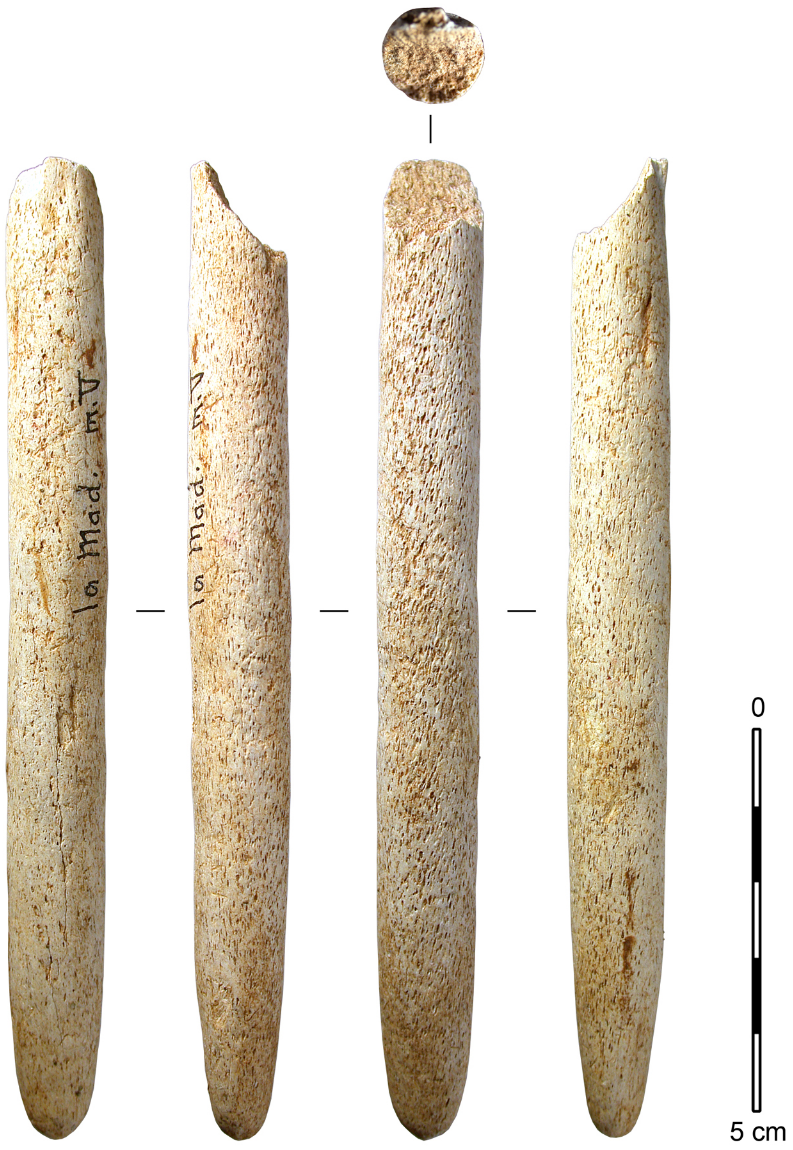

Supplementary Figure 36
Study number: 828
Museum number: CDP 79.1.267, S CV couche 2 (réattr. 3) 224
Site and layer: Duruthy 4
Typology: projectile point
ZooMS ID: Gray whale
^{14}C date: MUSE20134, 14870 ± 90 BP, 18062-17379 cal BP
(scale 1:2)

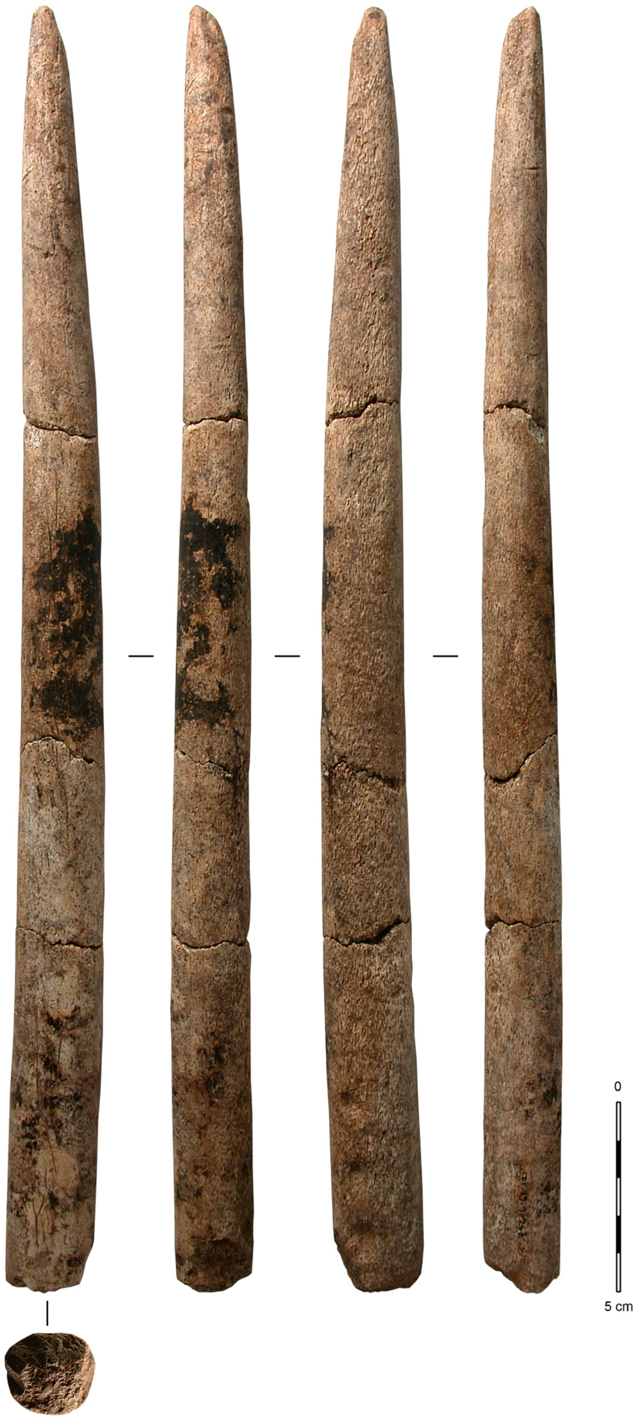

Supplementary Figure 37
Study number: 829
Museum number: CDP 79.1.77, S BV couche 1
Site and layer: Duruthy 3
Typology: unidentified
ZooMS ID: Fin whale
^{14}C date: MUSE20135, 14470 ± 90 BP, 17584-16913 cal BP
(photograph taken after sampling)

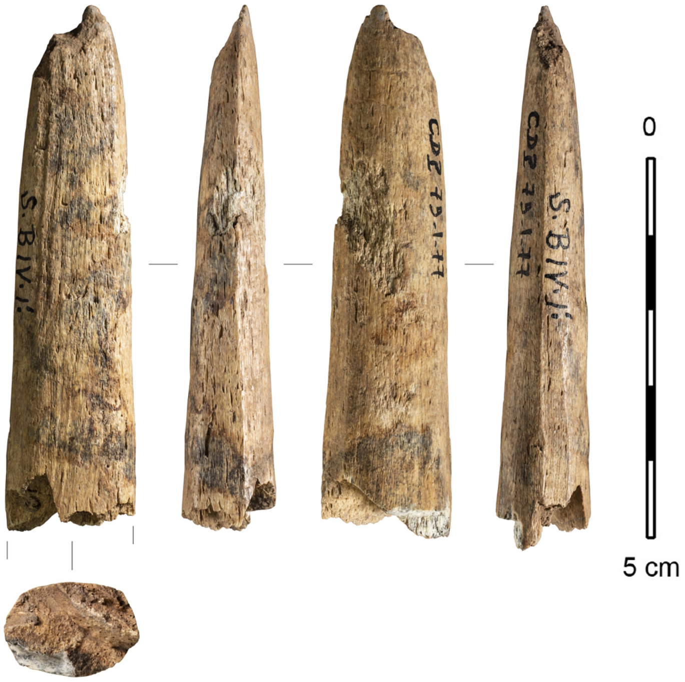

Supplementary Figure 38
Study number: 830
Museum number: CDP 79.1.18469, S BVI couche 1 n°48
Site and layer: Duruthy 3
Typology: unidentified
ZooMS ID: Right whale / Bowhead whale
^{14}C date: MUSE20136, failed
(photograph taken after sampling)

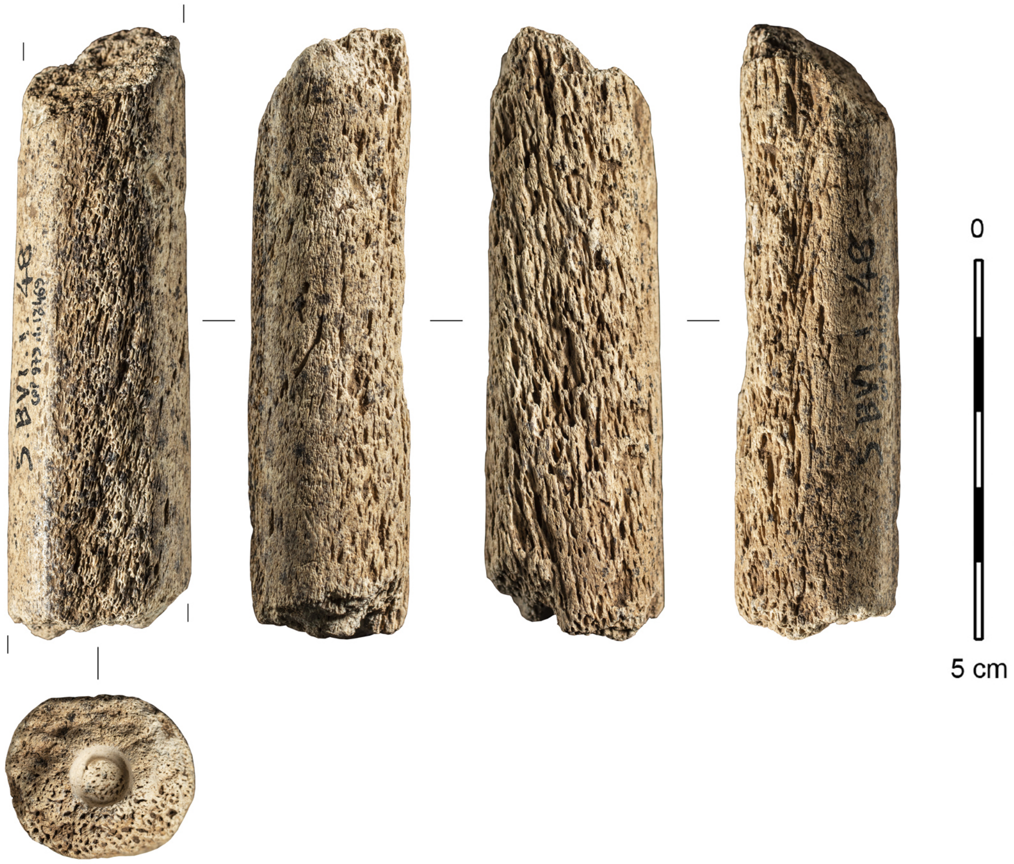

Supplementary Figure 39
Study number: 988
Museum number: CDP box 288-1
Site and layer: Duruthy 4
Typology: projectile point
ZooMS ID: Gray whale
^{14}C date: MUSE21107, 14330 ± 70 BP, 17346-16784 cal BP

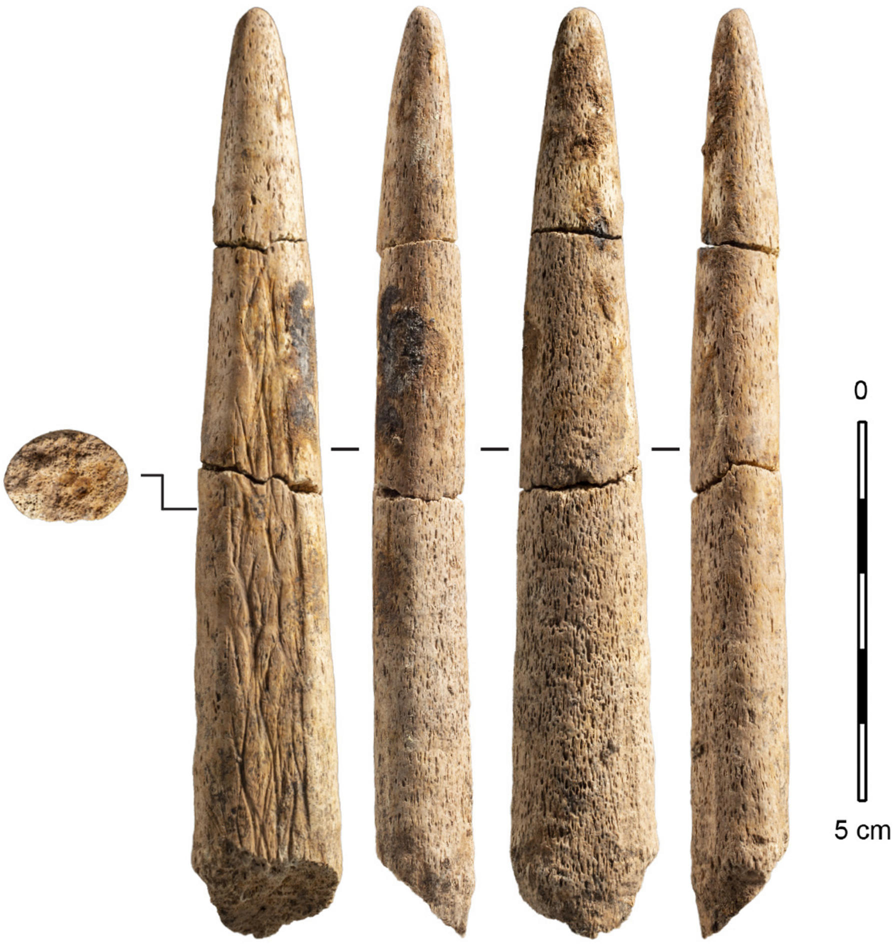

Supplementary Figure 40
Study number: 968
Museum number: ER III 13 (100-120)
Site and layer: Ermittia III
Typology: projectile point with massive base
ZooMS ID: Gray whale with polymer peaks
^{14}C date: MUSE21099, 14420 ± 50 BP, 16915-17416 cal BP

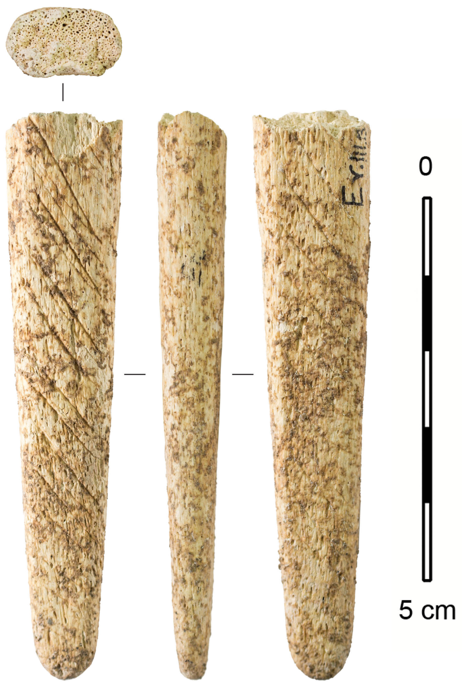

Supplementary Figure 41
Study number: 969
Museum number: ER III 85
Site and layer: Ermittia III
Typology: projectile point
ZooMS ID: Gray whale
^{14}C date: MUSE21100, 14520 ± 60 BP, 17004-17604 cal BP

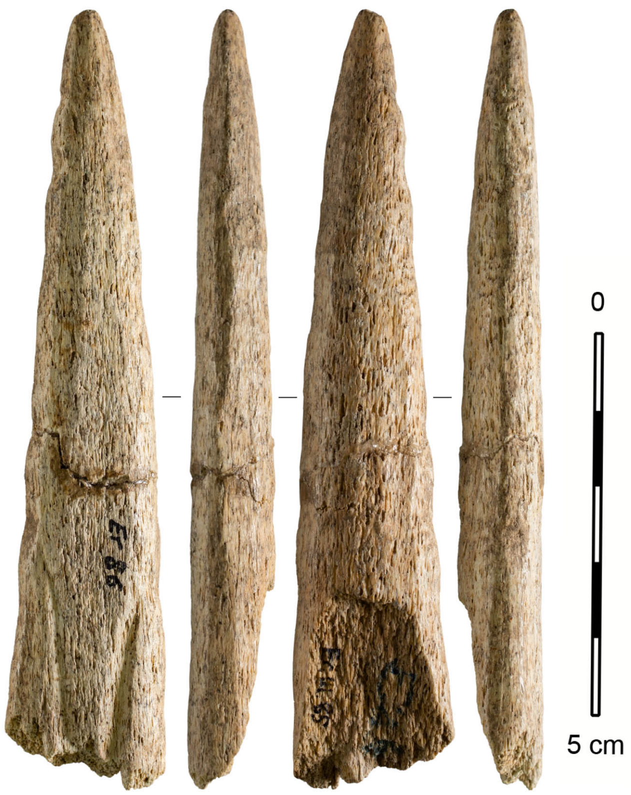

Supplementary Figure 42
Study number: 970
Museum number: UR 9D 350
Site and layer: Urtiaga D
Typology: unidentified
ZooMS ID: Gray whale
^{14}C date: MUSE21101, 14080 ± 50 BP, 16492-17017 cal BP

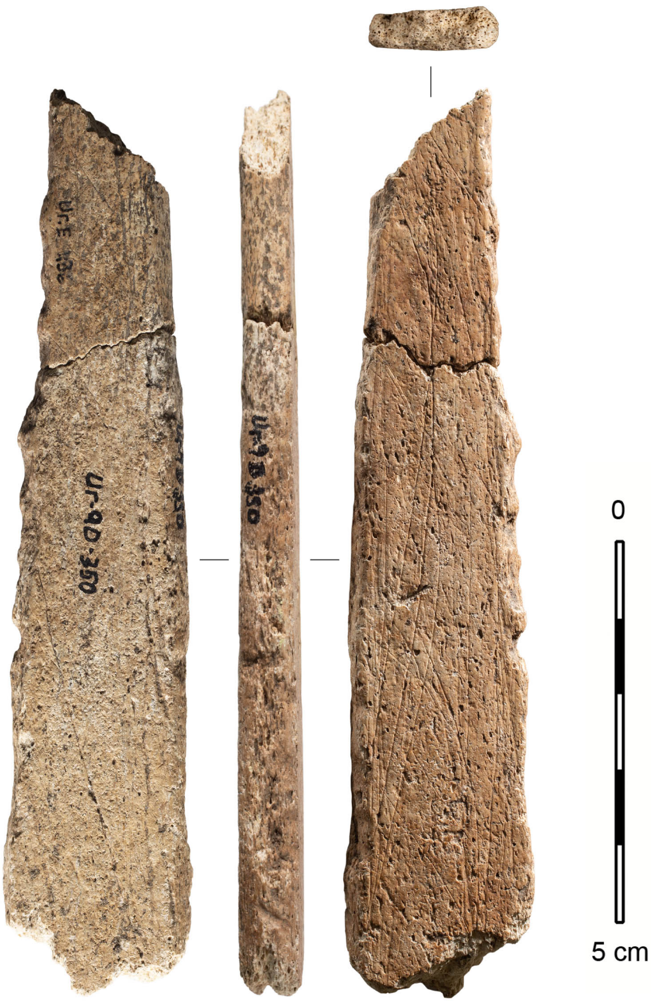

Supplementary Figure 43
Study number: 971
Museum number: ER III 4 (75-97)
Site and layer: Ermittia III
Typology: projectile point
ZooMS ID: Right whale / Bowhead whale
^{14}C date: MUSE21102, 14470 ± 60 BP, 16950-17513 cal BP
(scale 1:2)

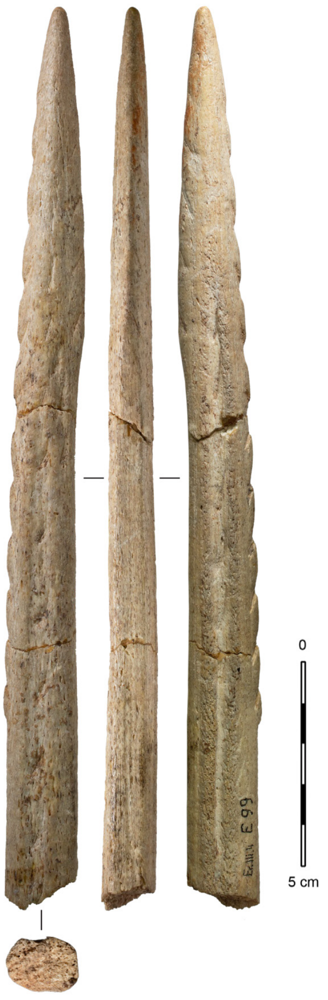

Supplementary Figure 44
Study number: 973
Museum number: JU 890014#3
Site and layer: El Juyo 8
Typology: blank?
ZooMS ID: Fin whale
^{14}C date: MUSE21104, 16290 ± 70 BP, 18992-19563 cal BP

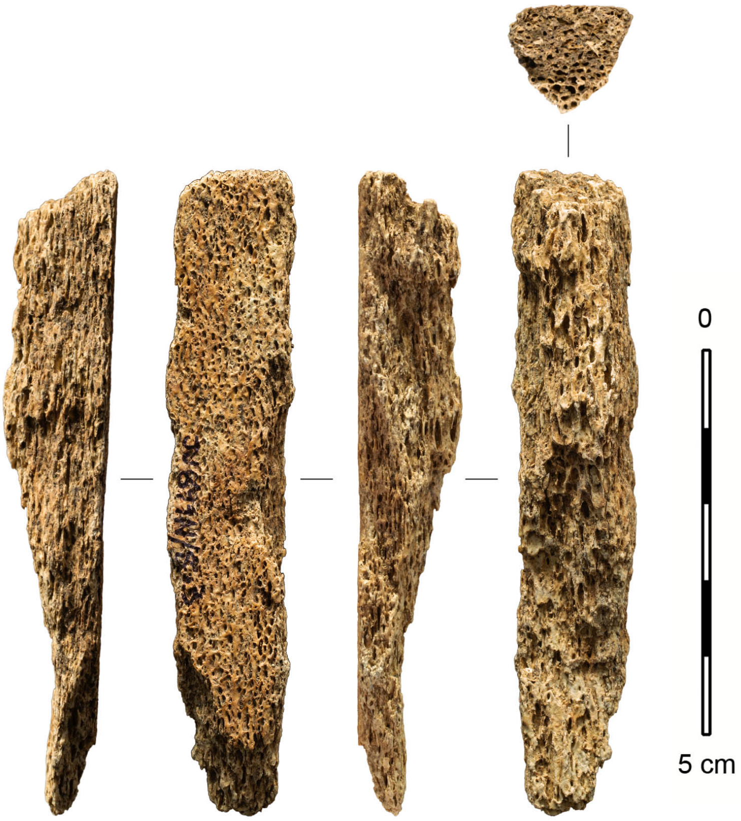

Supplementary Figure 45
Study number: 974
Museum number: Ra CE 11473
Site and layer: Rascaño IVb
Typology: object on splinter
ZooMS ID: Fin whale
^{14}C date: MUSE21105, 16770 ± 70 BP, 19569-20187 cal BP

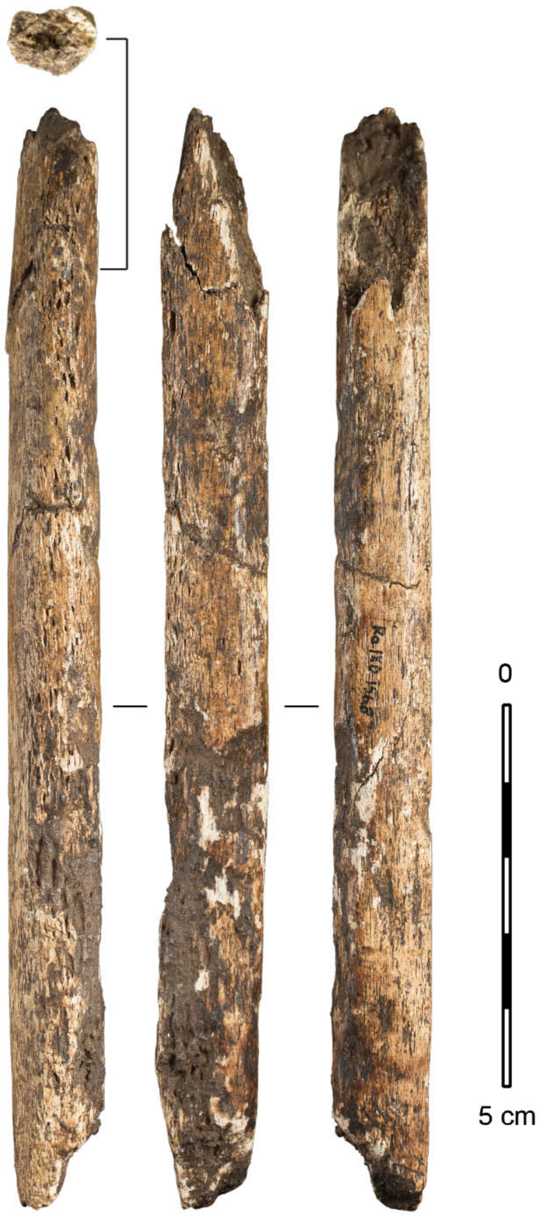

Supplementary Figure 46
Study number: 975
Museum number: 8638
Site and layer: Las Caldas, Sala II, IIB
Typology: object on splinter
ZooMS ID: Sperm whale
^{14}C date: MUSE21106, 13700 ± 50 BP, 16010-16549 cal BP

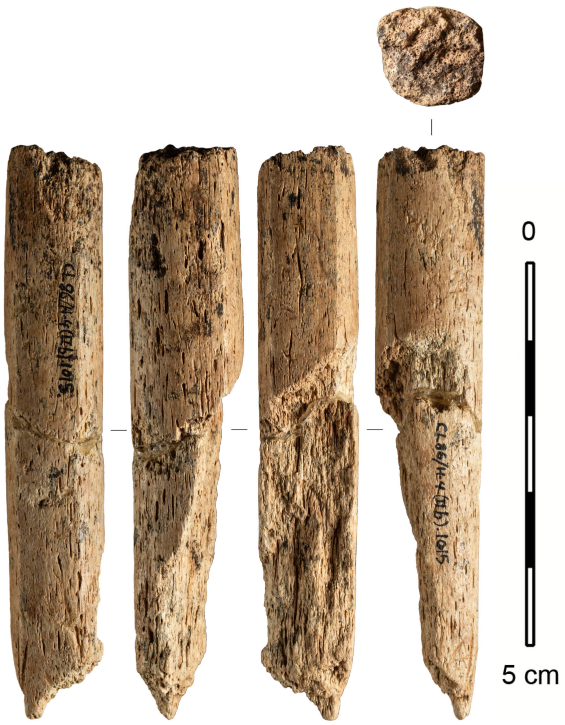

Supplementary Figure 47
Study number: 976
Museum number: 8640
Site and layer: Las Caldas, Sala II, IIIc-IV
Typology: projectile point with double bevel
ZooMS ID: Sperm whale
^{14}C date: none

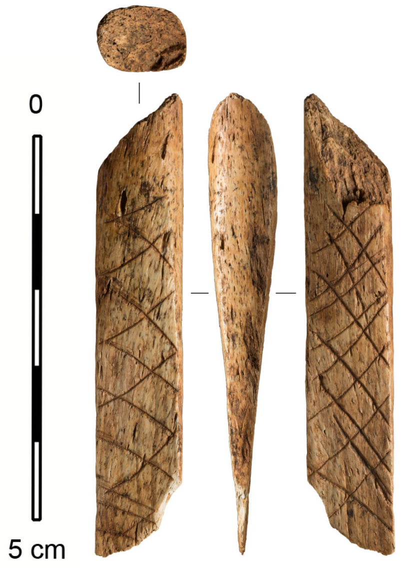

Supplementary Figure 48
Study number: 977
Museum number: 8644
Site and layer: Las Caldas, Sala II, V
Typology: object on splinter
ZooMS ID: Sperm whale
^{14}C date: none

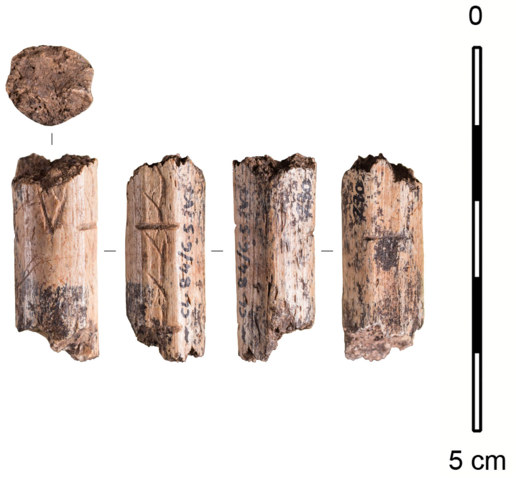

Supplementary Figure 49
Study number: 978
Museum number: 8645
Site and layer: Las Caldas, Sala II, VIIIb-IX
Typology: projectile point
ZooMS ID: Cetacean (poor spectra)
^{14}C date: none

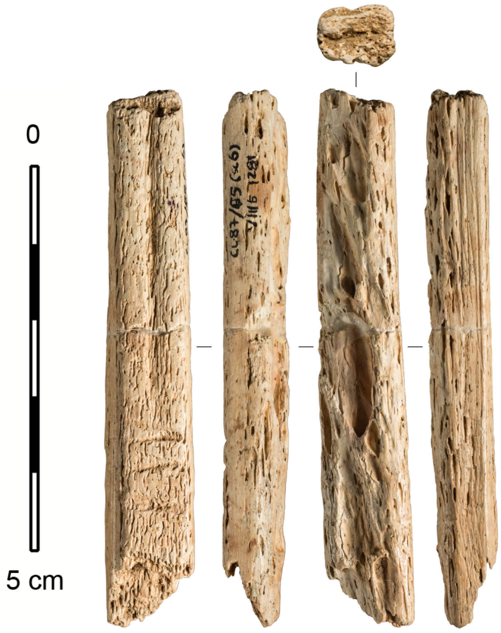

Supplementary Figure 50
Study number: 979
Museum number: 8646
Site and layer: Las Caldas, Sala II, VIII
Typology: projectile point
ZooMS ID: Dall's/Harbour porpoise (Phocoenidae)
^{14}C date: none

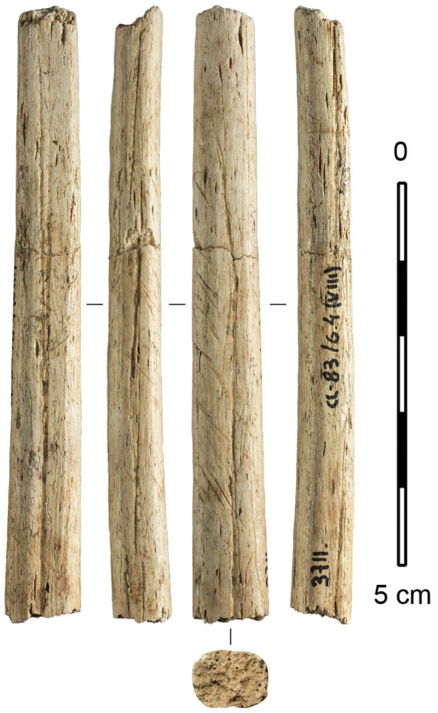

Supplementary Figure 51
Study number: 980
Museum number: 8647
Site and layer: Las Caldas, Sala II, VIb
Typology: projectile point
ZooMS ID: Blue whale
^{14}C date: none

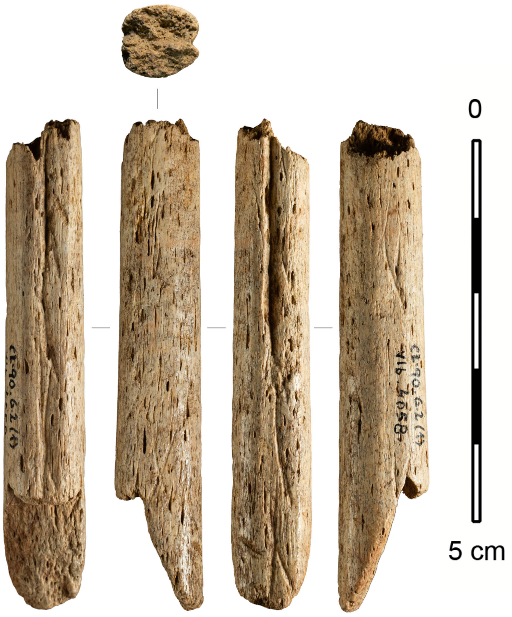

Supplementary Figure 52
Study number: 981
Museum number: 8648
Site and layer: Las Caldas, Sala II, VIII
Typology: projectile point
ZooMS ID: Sperm whale
^{14}C date: none

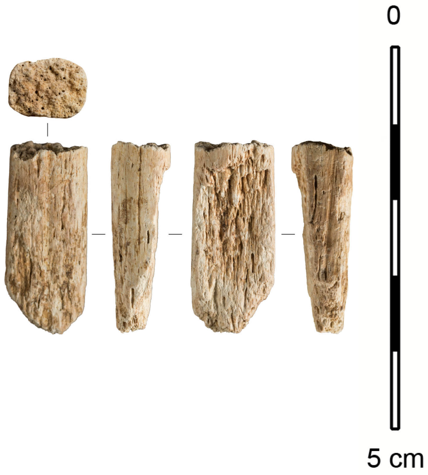

Supplementary Figure 53
Study number: 982
Museum number: 8650
Site and layer: Las Caldas, Sala II, VI
Typology: foreshaft?
ZooMS ID: Sperm whale
^{14}C date: none

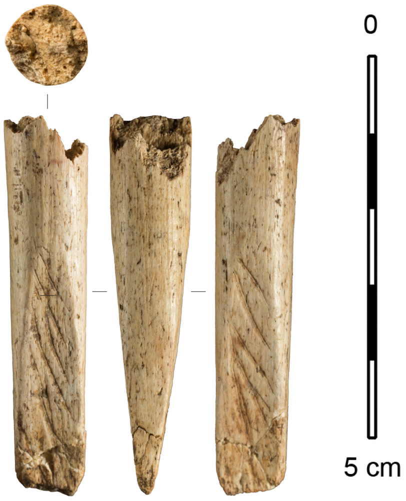

Supplementary Figure 54
Study number: 983
Museum number: 8658
Site and layer: Tito Bustillo, Área de Estancia, III
Typology: projectile point
ZooMS ID: Sperm whale
^{14}C date: none

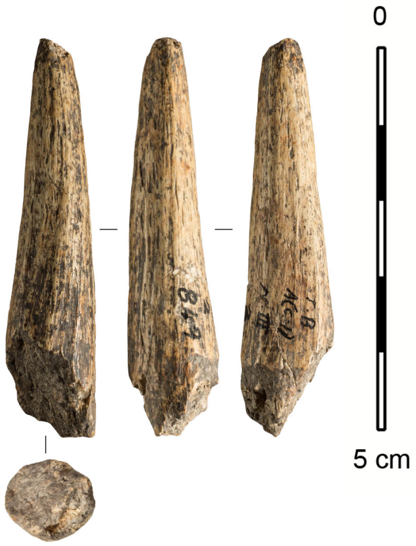

Supplementary Figure 55
Study number: 984
Museum number: 8666
Site and layer: Las Caldas, Sala II, VIII
Typology: unidentified
ZooMS ID: Right whale/Bowhead whale
^{14}C date: none

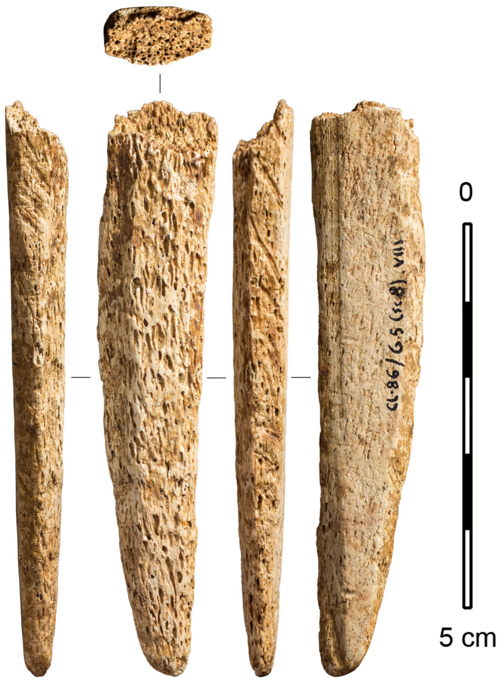

Supplementary Figure 56
Study number: 985
Museum number: 8699
Site and layer: La Viña IV
Typology: projectile point
ZooMS ID: Gray whale
^{14}C date: none

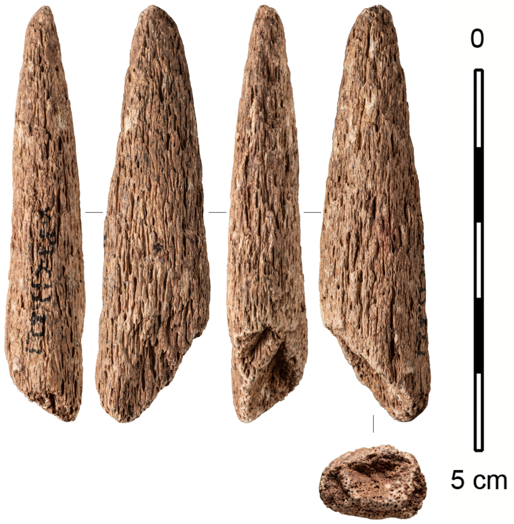

Supplementary Figure 57
Study number: 986
Museum number: 8701
Site and layer: La Viña IV
Typology: object on splinter
ZooMS ID: Gray whale
^{14}C date: none

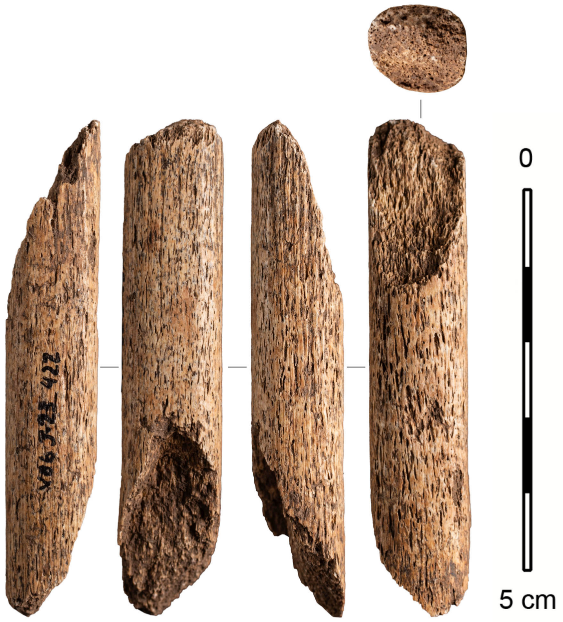

Supplementary Figure 58
Study number: 987
Museum number: 8703
Site and layer: La Viña IV
Typology: projectile point?
ZooMS ID: Sperm whale
^{14}C date: none

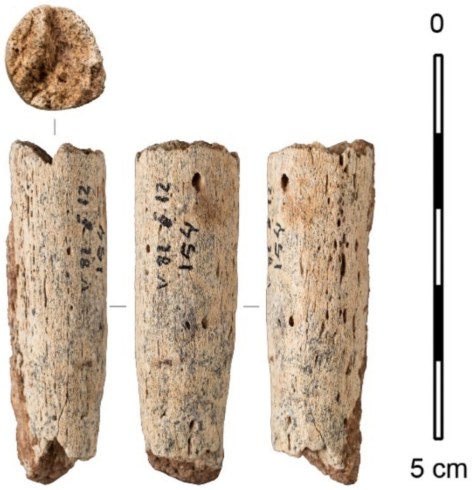

Supplementary Figure 59
Study number: Hum1
Museum number: TB-74
Site and layer: Tito Bustillo, Área de Estancia, 1B
Typology: blank?
ZooMS ID: Sperm whale
^{14}C date: none

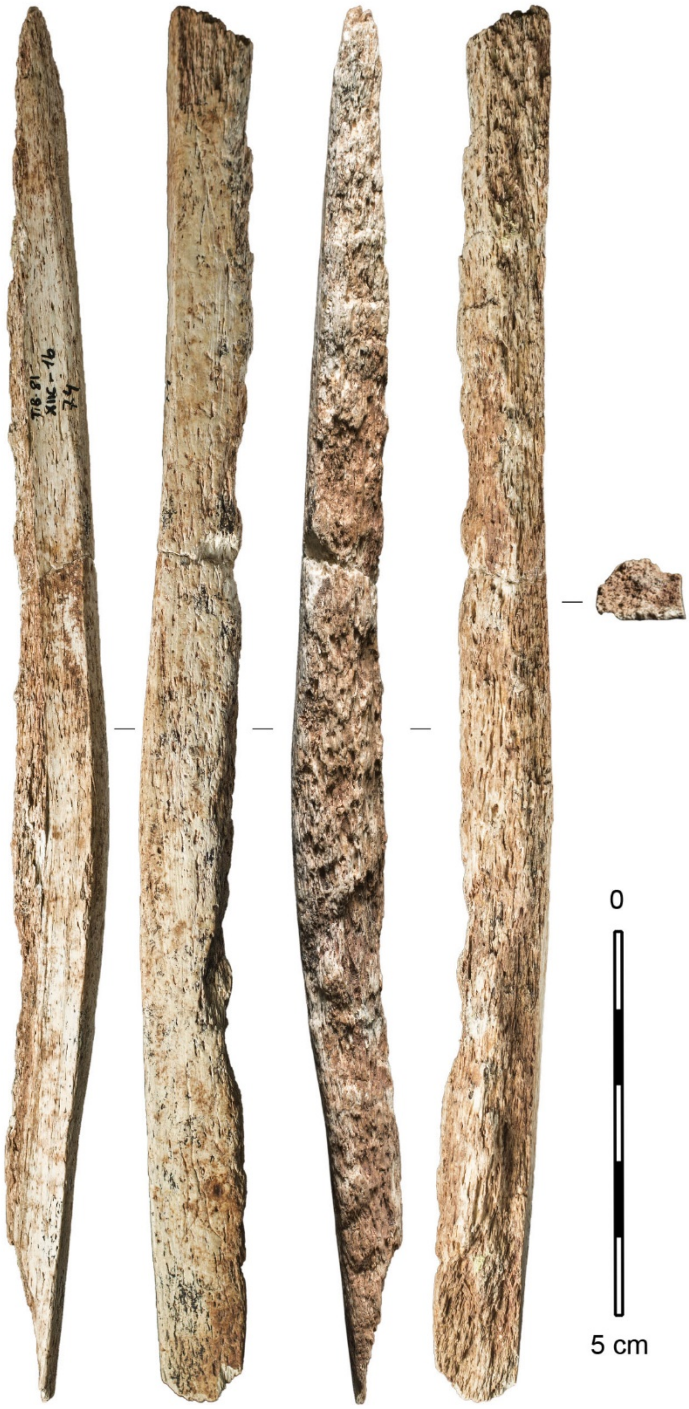

Supplementary Figure 60
Study number: Hum4
Museum number: 14-VII-98
Site and layer: Iruroin
Typology: object on splinter
ZooMS ID: Sperm whale
^{14}C date: UCIAMS289875, 13880 ± 50 BP, 16219-16780 cal BP

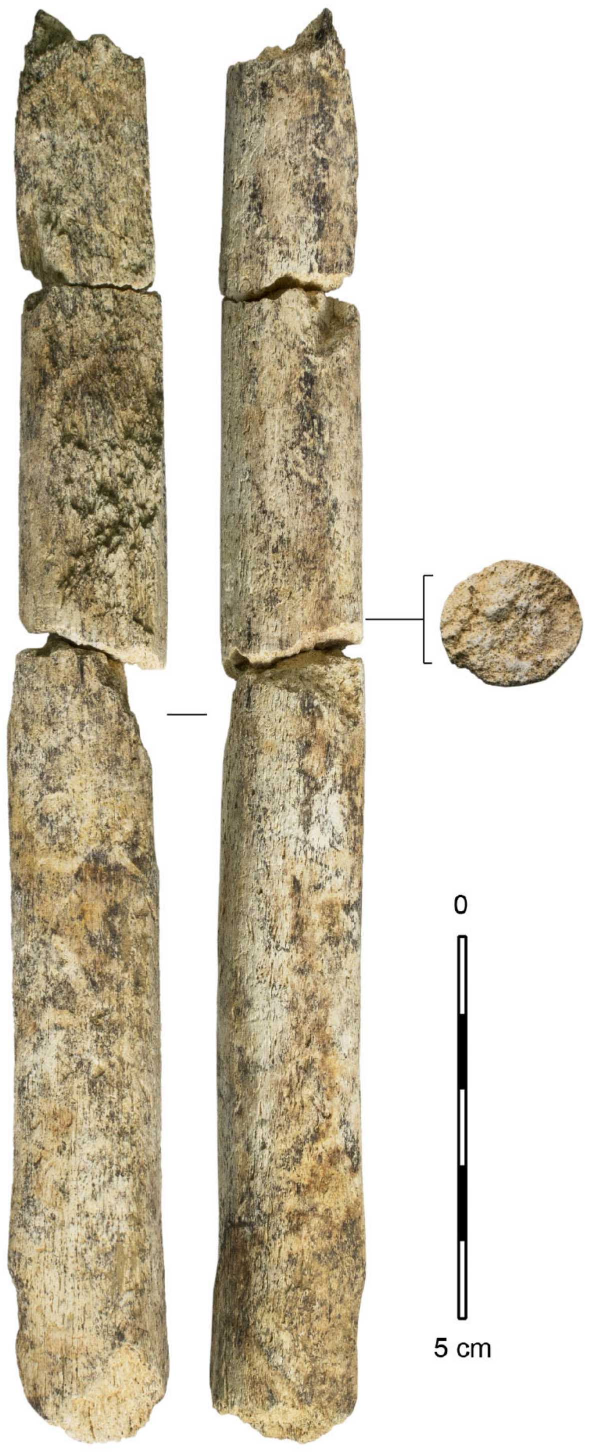

Supplementary Figure 61
Study number: Hum8
Museum number: 6889
Site and layer: La Paloma 6
Typology: projectile point
ZooMS ID: Sperm whale
^{14}C date: UCIAMS289876, 14420 ± 60 BP, 16887-17415 cal BP

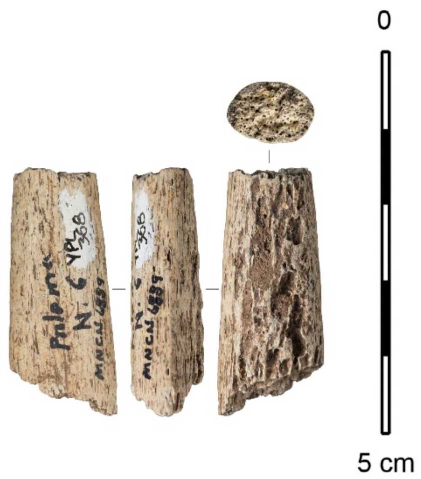

Supplementary Figure 62
Study number: Hum9
Museum number: 6761
Site and layer: La Paloma 4
Typology: projectile point
ZooMS ID: Fin whale
^{14}C date: R00217.1, 14284 ± 46 BP, 16730-17265 cal BP

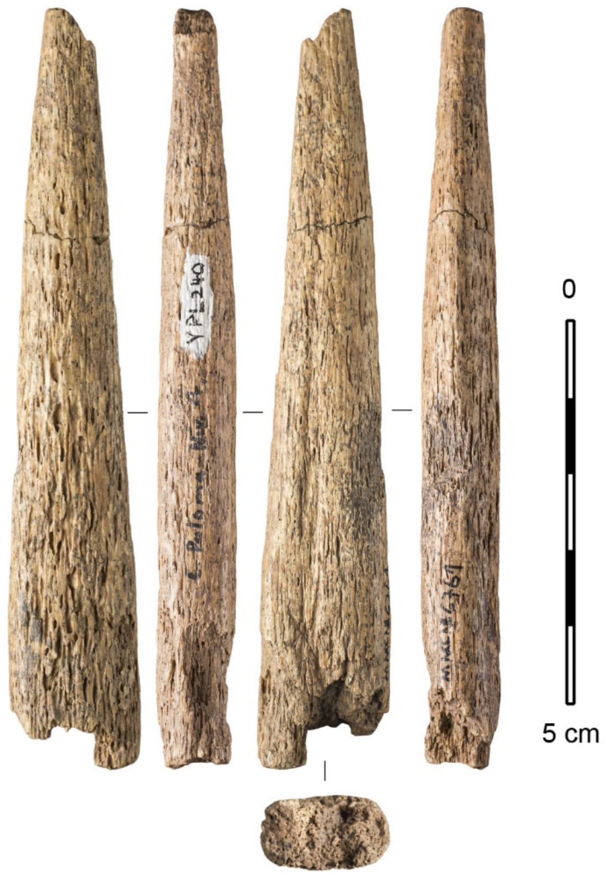

Supplementary Figure 63
Study number: Hum14
Museum number: MNP-2012-3-1-12
Site and layer: Bourrouilla, déblais fouilles clandestines
Typology: unidentified
ZooMS ID: Fin whale
^{14}C date: R00222.1, 14046 ± 110 BP, 16340-17056 cal BP

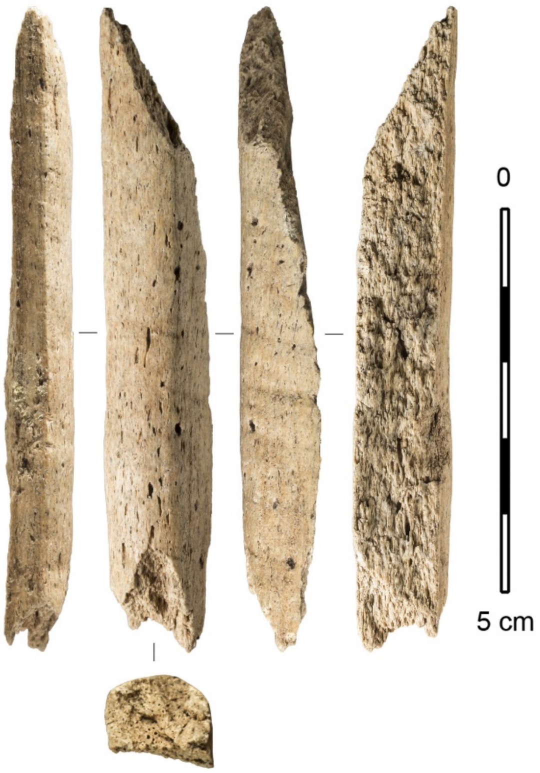

Supplementary Figure 64
Study number: Hum18
Museum number: PBII-75/16/2/76
Site and layer: El Pendo II
Typology: projectile point
ZooMS ID: Sperm whale
^{14}C date: R00225.1, 15549 ± 56 BP, 18222-18703 cal BP

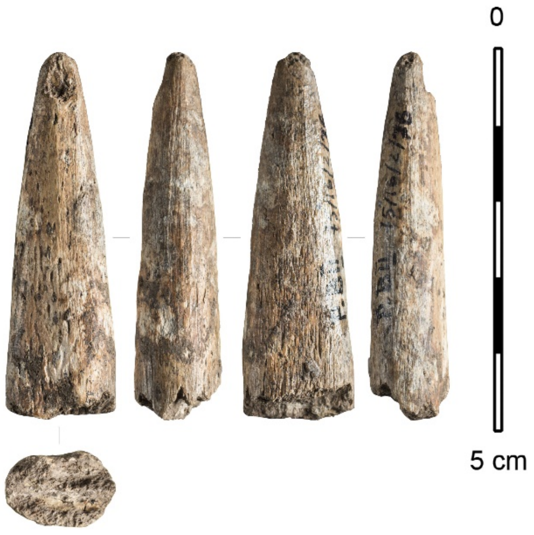

Supplementary Figure 65
Study number: SC A6 127 68
Museum number: SC A6 127 68
Site and layer: Santa Catalina III
Typology: unworked
ZooMS ID: Right whale / Bowhead whale
^{14}C dates:
MUSE21034, 13800 ± 60 BP, 16701-16119 cal BP
MUSE20050, 13770 ± 60 BP, 16657-16081 cal BP
MUSE20051, 13670 ± 60 BP, 16527-15959 cal BP
(scale 1:4)

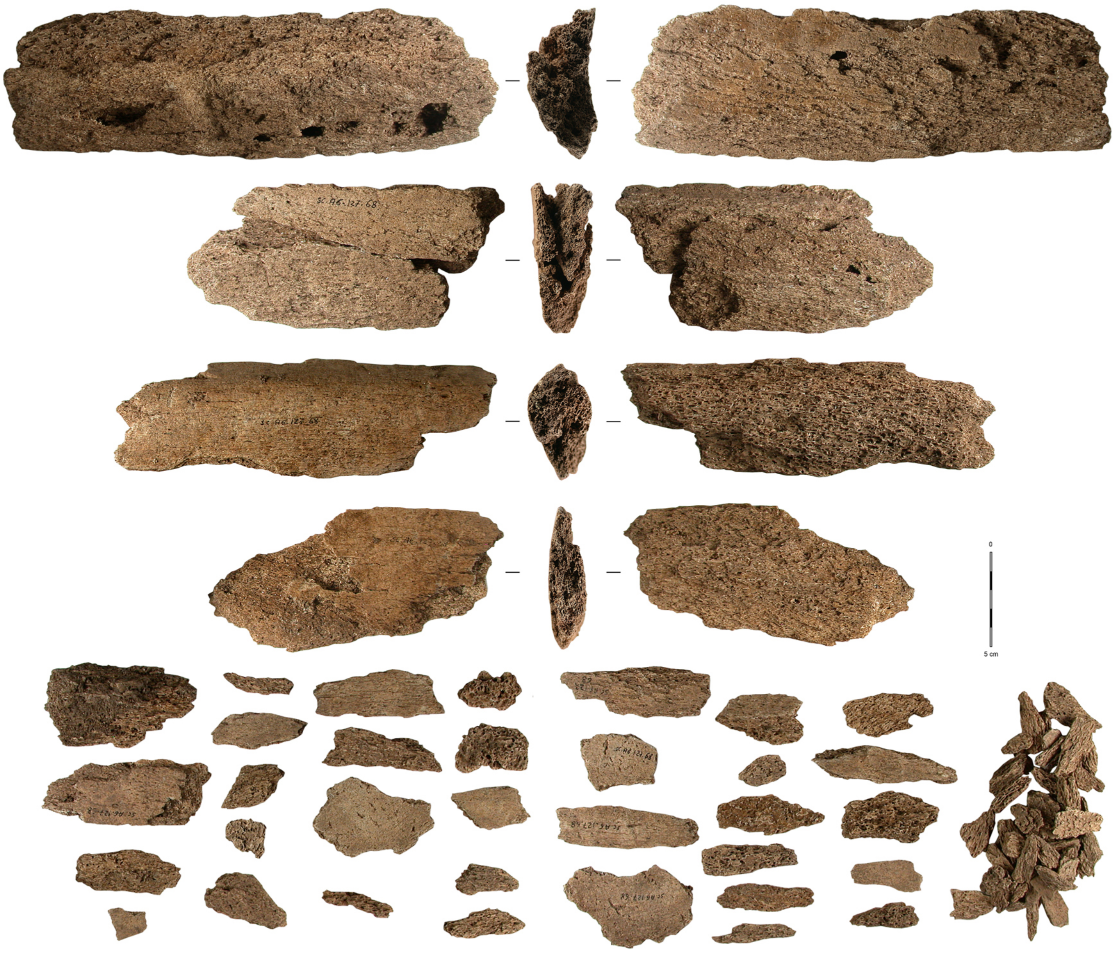

Supplementary Figure 66
Study number: SC A6 130 15
Museum number: SC A6 130 15
Site and layer: Santa Catalina III
Typology: unworked
ZooMS ID: Fin whale
^{14}C date: MUSE20065, 12790 ± 70 BP, 15385-14803 cal BP
(photograph taken after sampling)

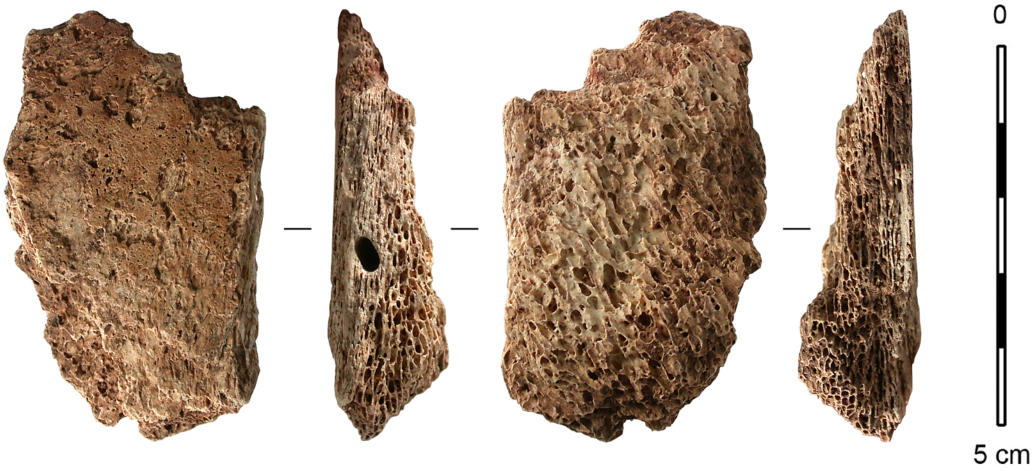

Supplementary Figure 67
Study number: SC A6 L31G 2971
Museum number: SC A6 L31G 2971
Site and layer: Santa Catalina III
Typology: unworked
ZooMS ID: Right whale / Bowhead whale
^{14}C date: MUSE20049, 13720 ± 90 BP, 16643-15972 cal BP

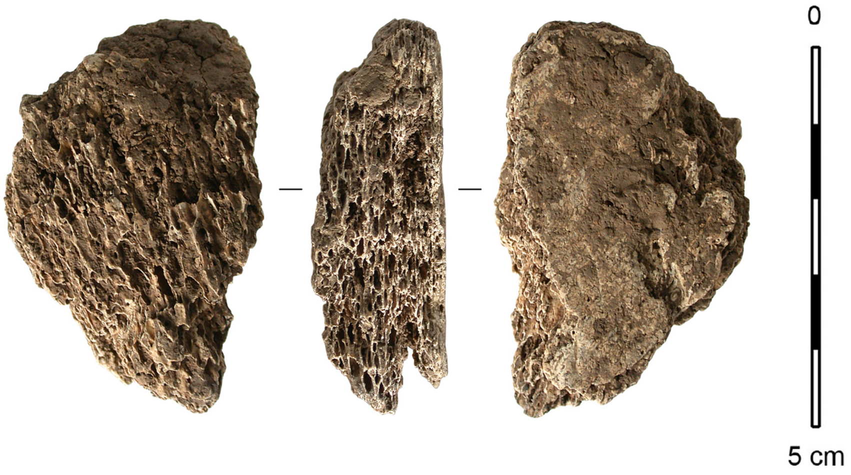

Supplementary Figure 68
Study number: SC A8 118 63
Museum number: SC A8 118 63
Site and layer: Santa Catalina III
Typology: unworked
ZooMS ID: Fin whale
^{14}C date: MUSE20037, 13040 ± 50 BP, 15672-15153 cal BP

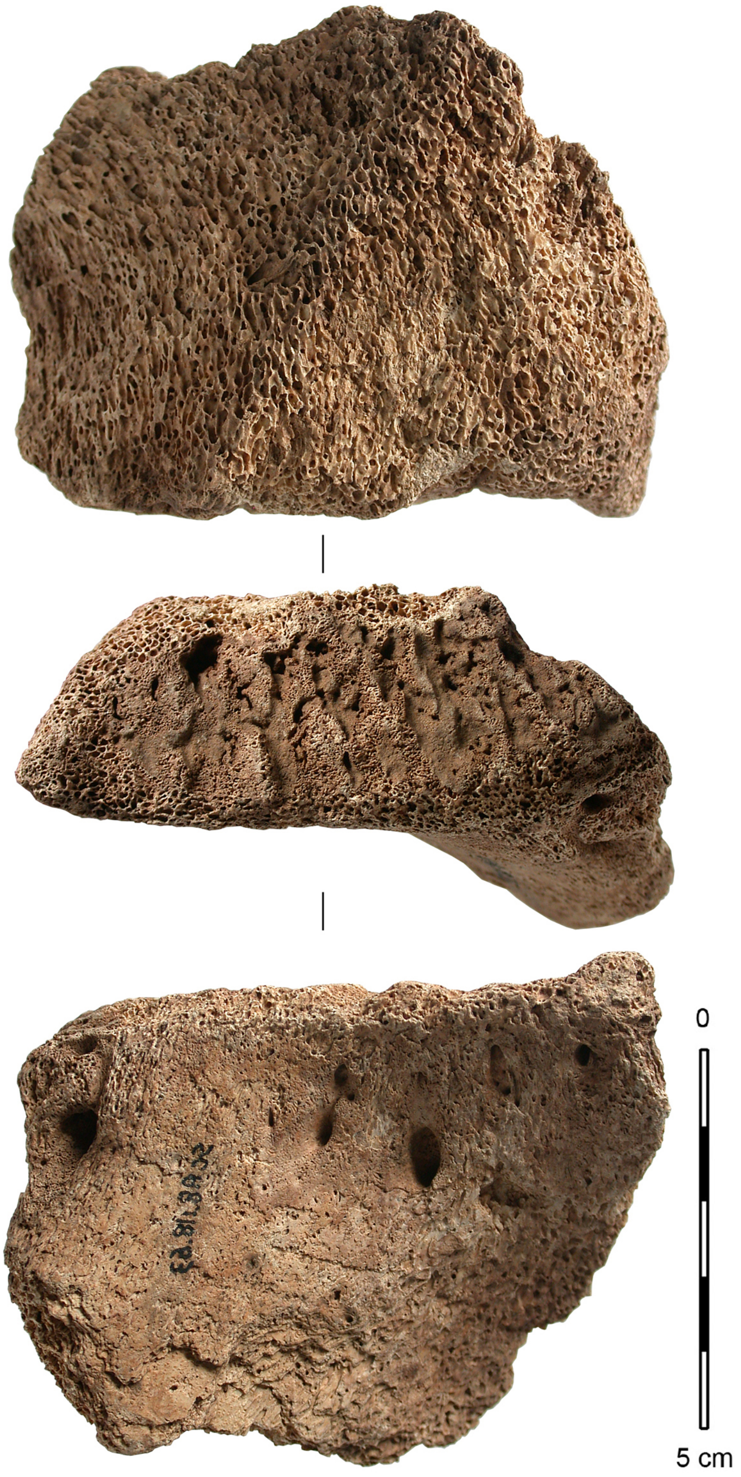

Supplementary Figure 69
Study number: SC A8 83 486
Museum number: SC A8 83 486
Site and layer: Santa Catalina II
Typology: unworked
ZooMS ID: Fin whale
^{14}C date: MUSE20034, 12700 ± 70 BP, 15259-14609 cal BP

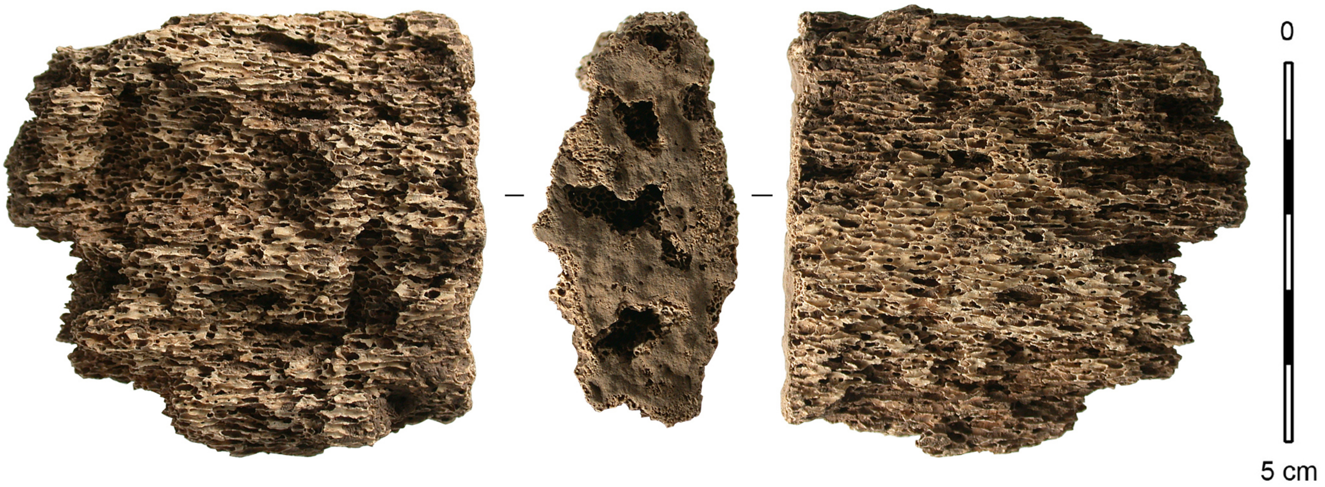

Supplementary Figure 70
Study number: SC B4 87 76
Museum number: SC B4 87 76
Site and layer: Santa Catalina II
Typology: unworked
ZooMS ID: Fin whale
^{14}C date: MUSE20035, 12990 ± 50 BP, 15611-15096 cal BP

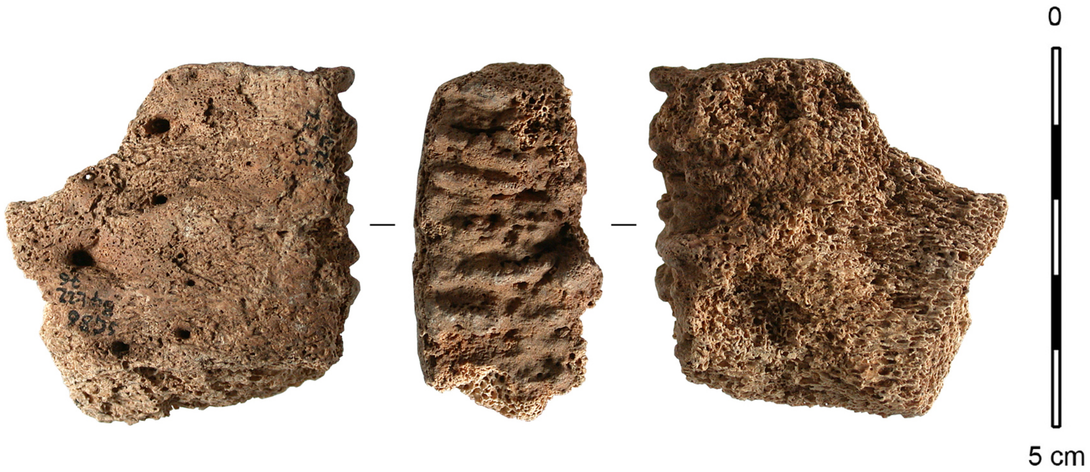

Supplementary Figure 71
Study number: SC B4 87 77
Museum number: SC B4 87 77
Site and layer: Santa Catalina II
Typology: unworked
ZooMS ID: Fin whale
^{14}C date: MUSE20066, 12670 ± 60 BP, 15204-14850 cal BP
(photograph taken after sampling)

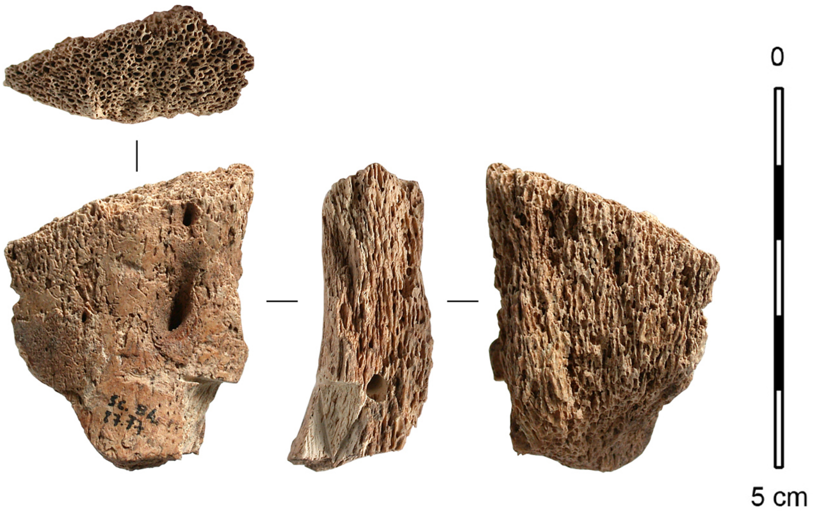

Supplementary Figure 72
Study number: SC B6 109 1085
Museum number: SC B6 109 1085
Site and layer: Santa Catalina III
Typology: unworked
ZooMS ID: Fin whale
^{14}C date: MUSE20038, 13010 ± 50 BP, 15634-15119 cal BP

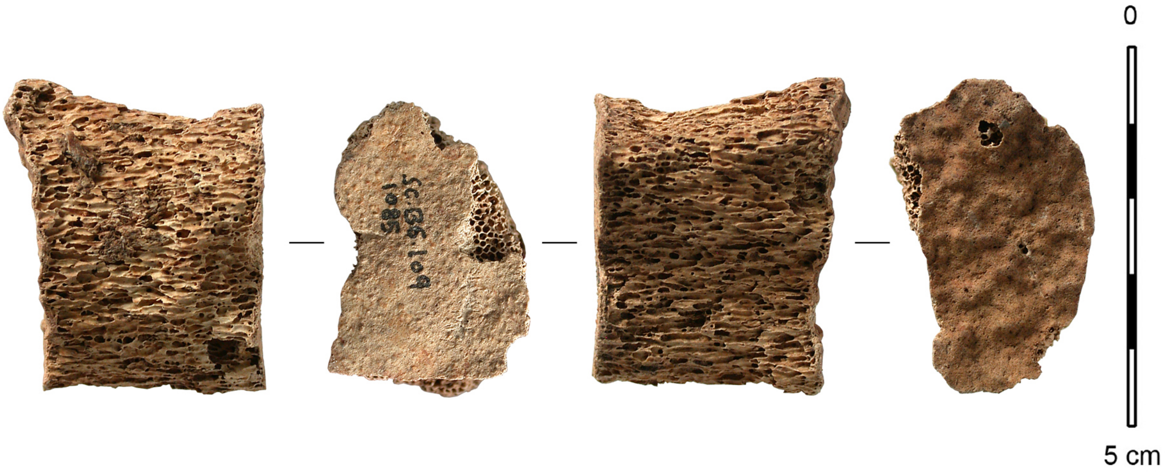

Supplementary Figure 73
Study number: SC B6 135 24
Museum number: SC B6 135 24
Site and layer: Santa Catalina III
Typology: unworked
ZooMS ID: Fin whale
^{14}C date: MUSE20039, 13060 ± 50 BP, 15699-15177 cal BP

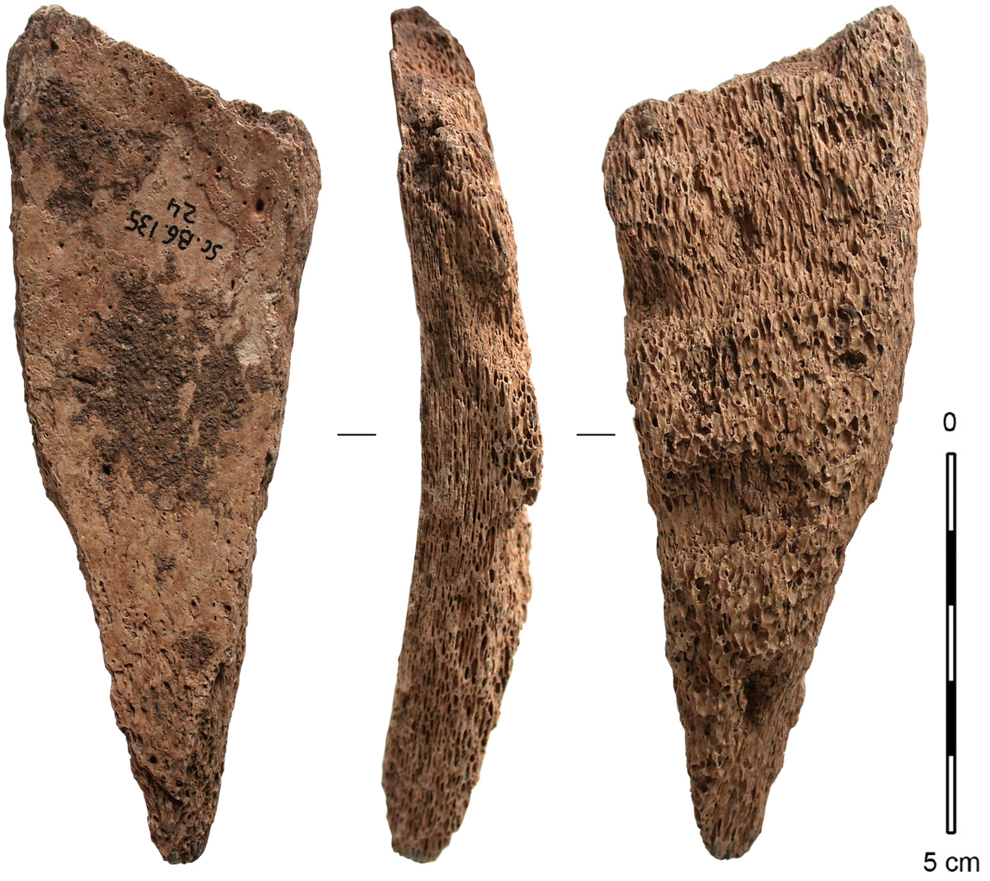

Supplementary Figure 74
Study number: SC B6 151 48
Museum number: SC B6 151 48
Site and layer: Santa Catalina III
Typology: unworked
ZooMS ID: Fin whale
^{14}C date: MUSE20067, 12990 ± 60 BP, 15624-15082 cal BP

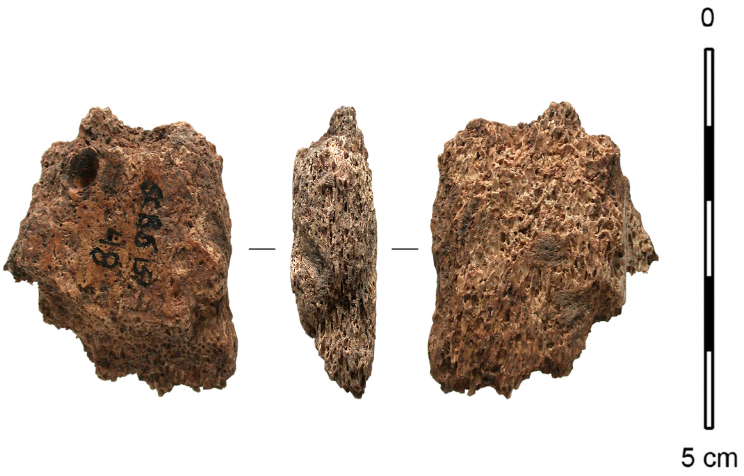

Supplementary Figure 75
Study number: SC B6 152 44
Museum number: SC B6 152 44
Site and layer: Santa Catalina III
Typology: unworked
ZooMS ID: Fin whale
^{14}C date: MUSE20068, 13170 ± 60 BP, 15868-15293 cal BP
(photograph taken after sampling)

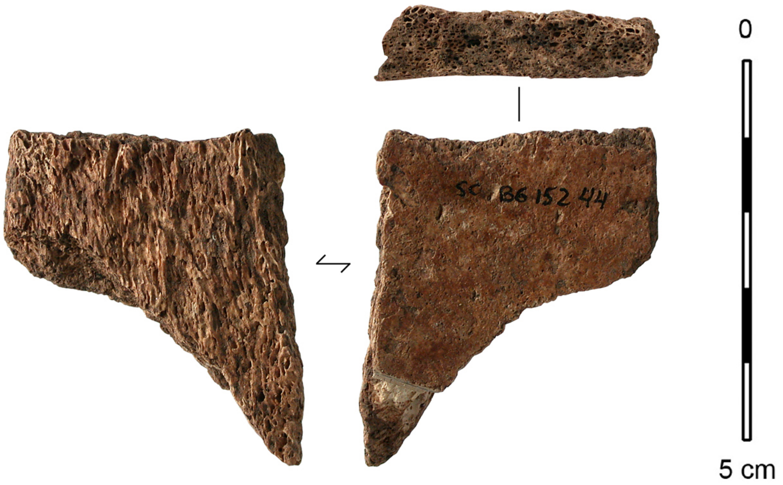

Supplementary Figure 76
Study number: SC B6 83 67
Museum number: SC B6 83 67
Site and layer: Santa Catalina II
Typology: unworked
ZooMS ID: Fin whale
^{14}C date: MUSE20036, 12970 ± 50 BP, 15588-15072 cal BP

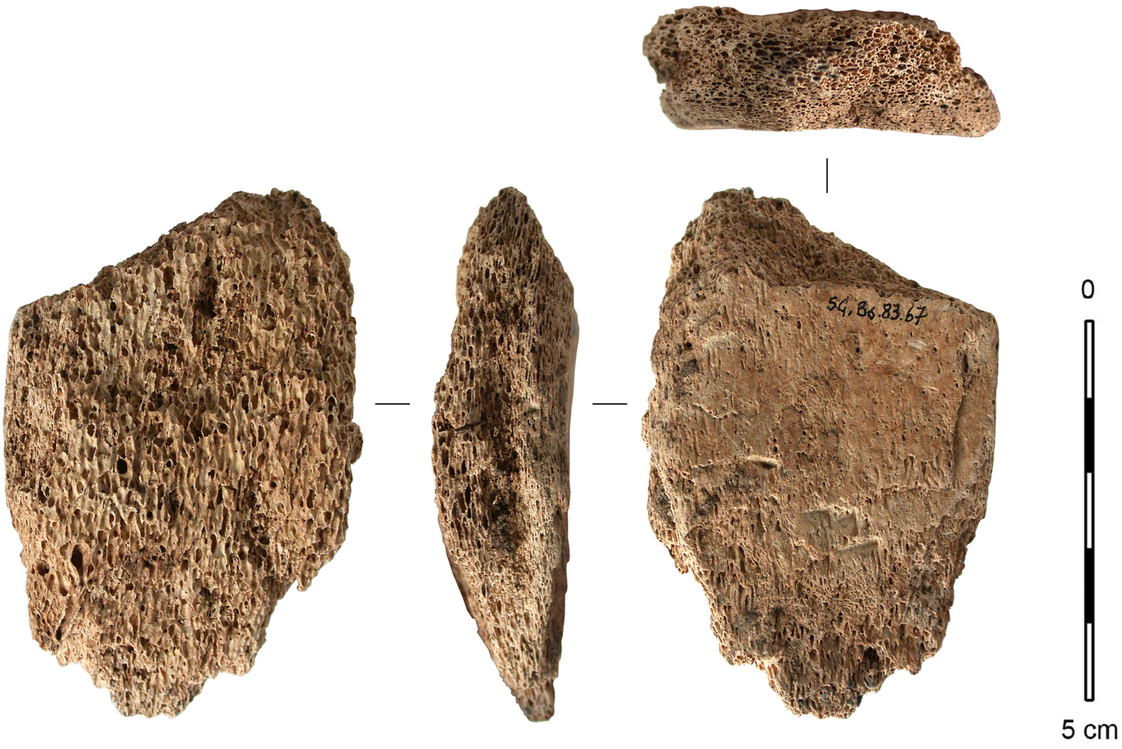

Supplementary Figure 77
Study number: SC B8 124 1180
Museum number: SC B8 124 1180
Site and layer: Santa Catalina **III**
Typology: unworked
ZooMS ID: Fin whale
^{14}C date: MUSE20041, 13090 ± 50 BP, 15740-15212 cal BP

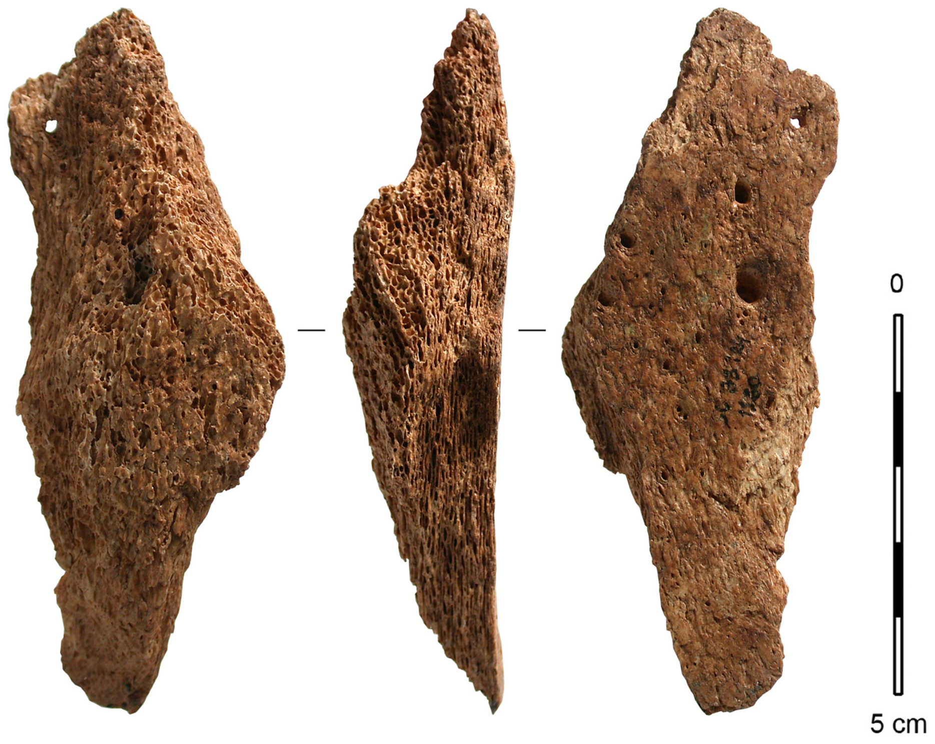

Supplementary Figure 78
Study number: SC B8 143 1392
Museum number: SC B8 143 1392
Site and layer: Santa Catalina III
Typology: unworked
ZooMS ID: Fin whale / Right whale / Bowhead whale
^{14}C date: MUSE20043, 13040 ± 50 BP, 15672-15153 cal BP

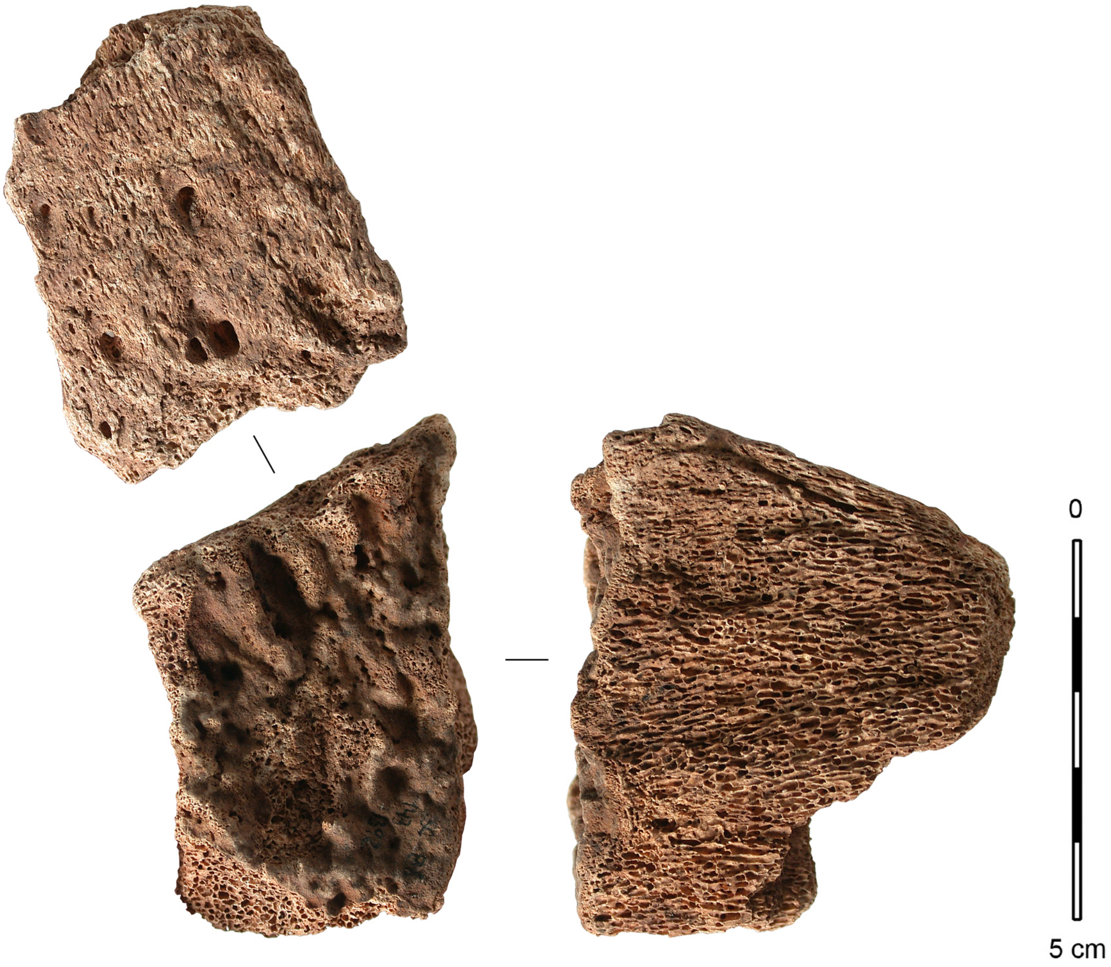

Supplementary Figure 79
Study number: SC B8 144 439
Museum number: SC B8 144 439
Site and layer: Santa Catalina **III**
Typology: unworked
ZooMS ID: Fin whale
^{14}C date: MUSE20045, 13090 ± 50 BP, 15740-15212 cal BP

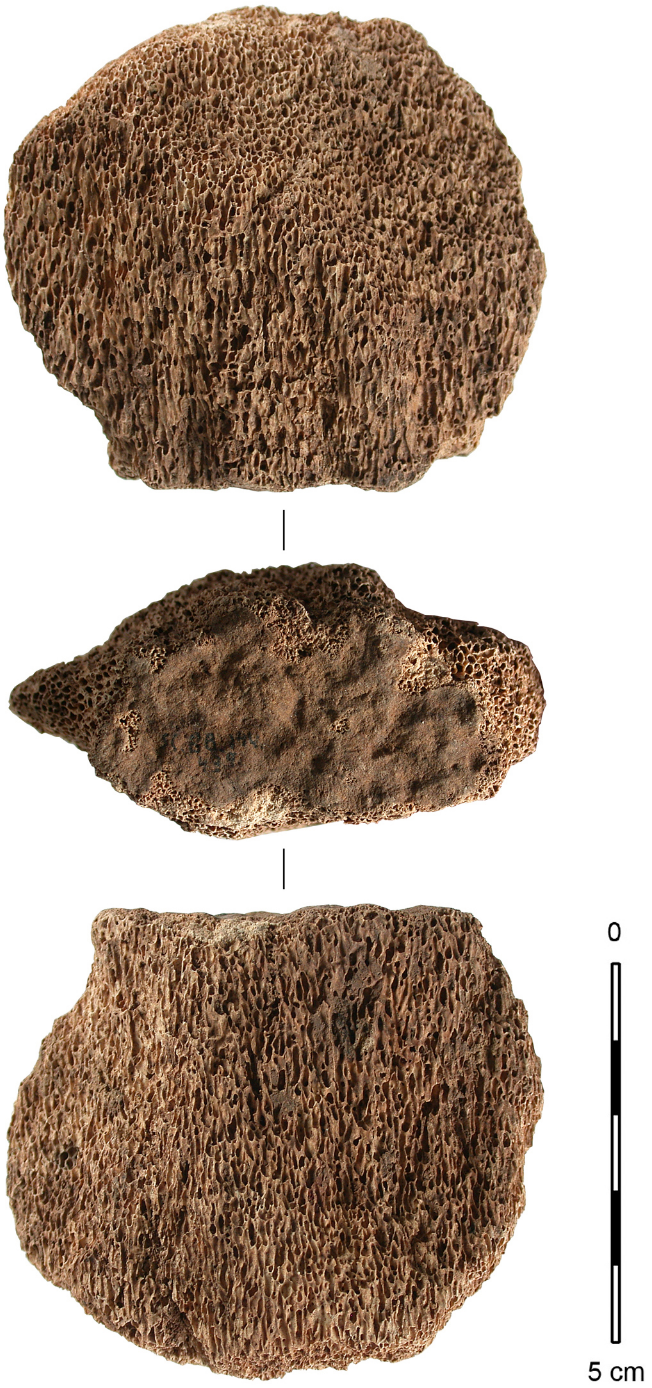

Supplementary Figure 80
Study number: SC B8 151 477
Museum number: SC B8 151 477
Site and layer: Santa Catalina III
Typology: unworked
ZooMS ID: Fin whale
^{14}C date: MUSE20047, 13080 ± 50 BP, 15727-15201 cal BP

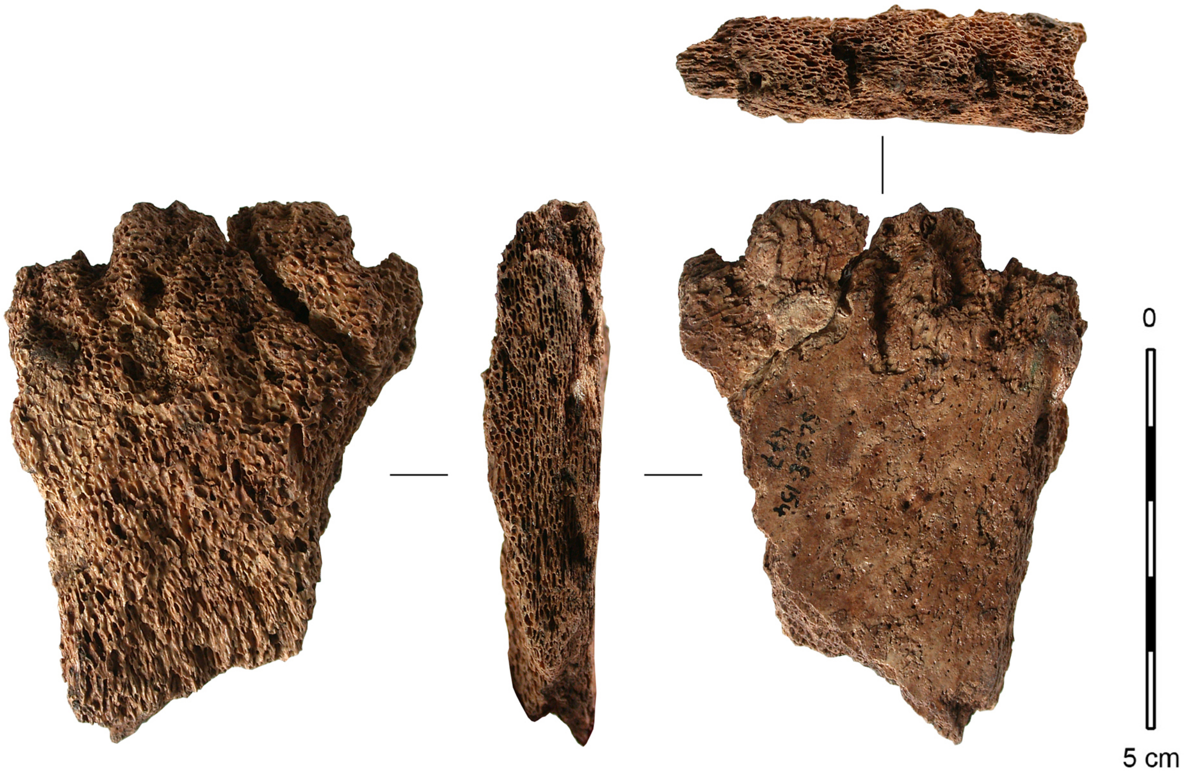

Supplementary Figure 81
Study number: SC B8 153 130
Museum number: SC B8 153 130
Site and layer: Santa Catalina III
Typology: unworked
ZooMS ID: Fin whale
^{14}C date: MUSE20069, 13010 ± 60 BP, 15648-15106 cal BP
(photograph taken after sampling)

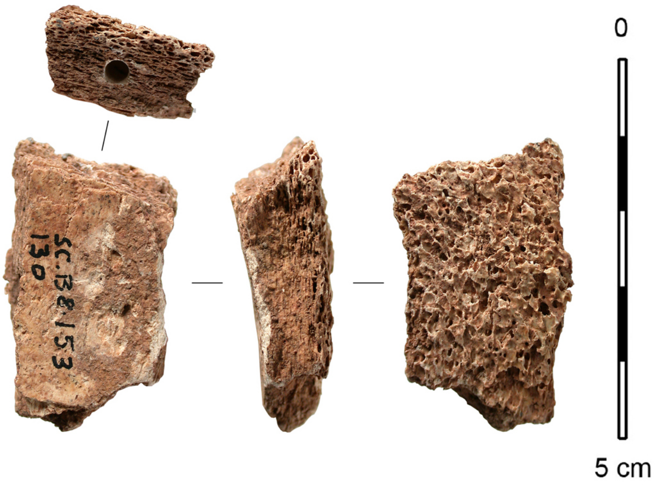

Supplementary Figure 82
Study number: SC B8 153 147
Museum number: SC B8 153 147
Site and layer: Santa Catalina **III**
Typology: unworked
ZooMS ID: Fin whale
^{14}C date: MUSE20033, 13070 ± 60 BP, 15726-15175 cal BP
(photograph taken after sampling)

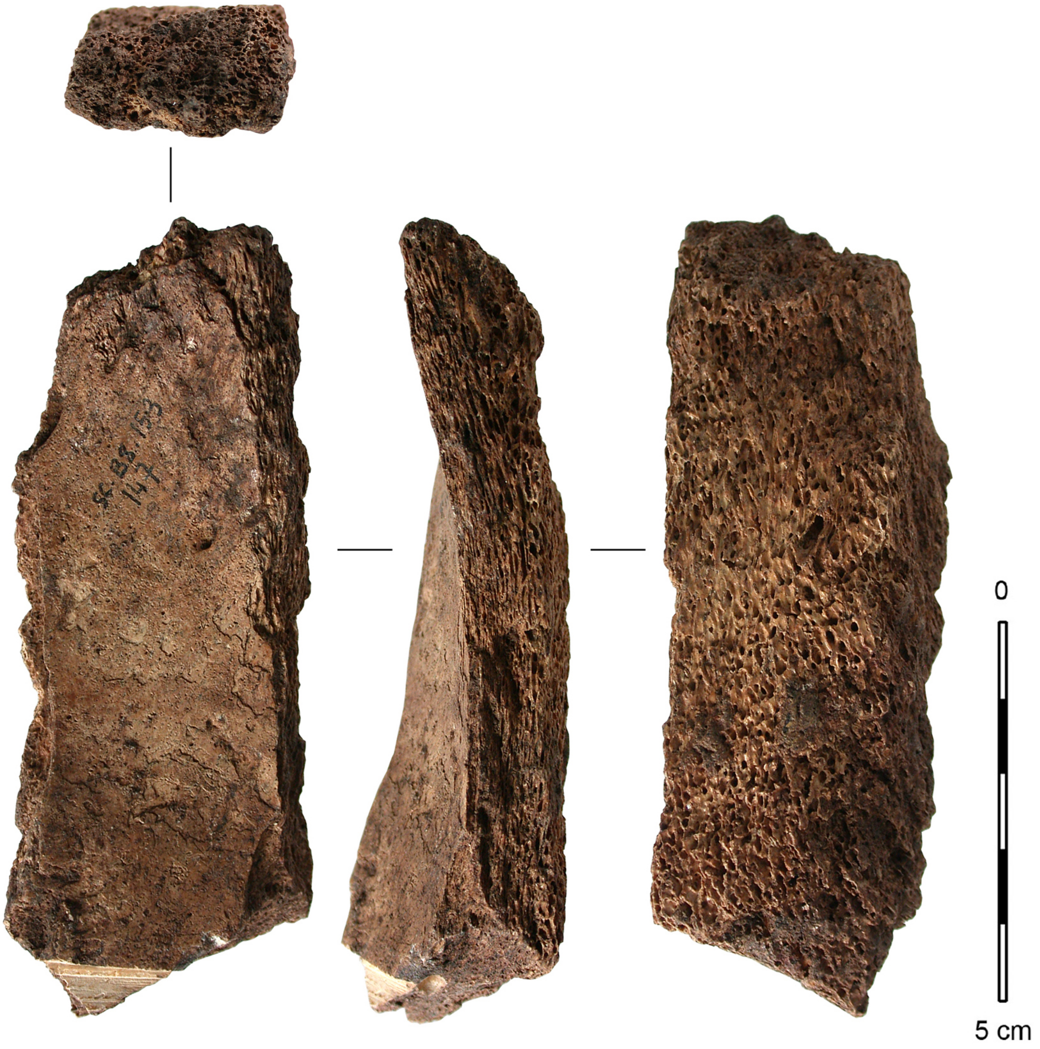

Supplementary Figure 83
Study number: SC B8 154 129
Museum number: SC B8 154 129
Site and layer: Santa Catalina III
Typology: unworked
ZooMS ID: Fin whale
^{14}C date: MUSE20071, 13130 ± 60 BP, 15811-15246 cal BP
(photograph taken after sampling)

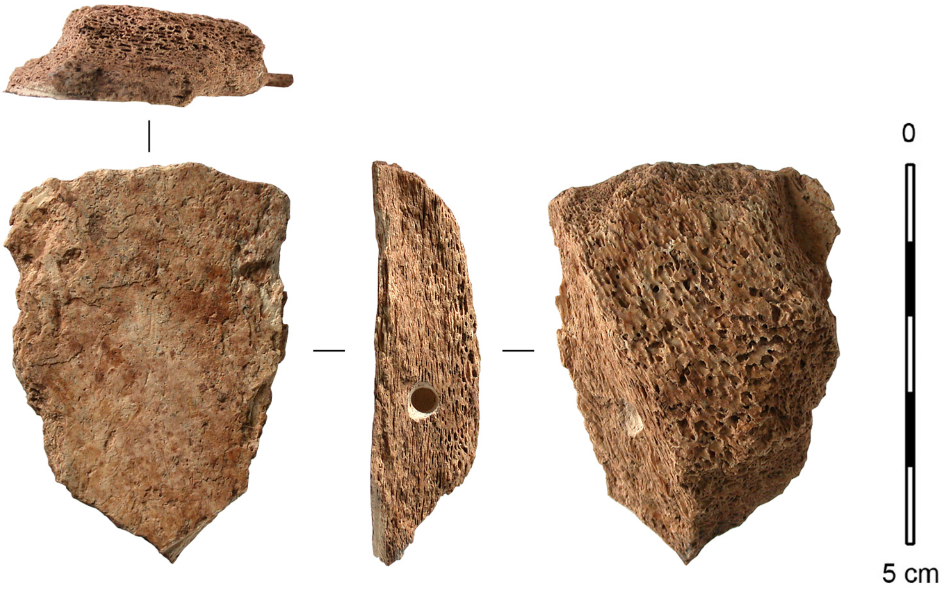

Supplementary Figure 84
Study number: SC B8 154 150
Museum number: SC B8 154 150
Site and layer: Santa Catalina III
Typology: unworked
ZooMS ID: Fin whale / Gray whale / Humpback whale
^{14}C date: MUSE20048, failed

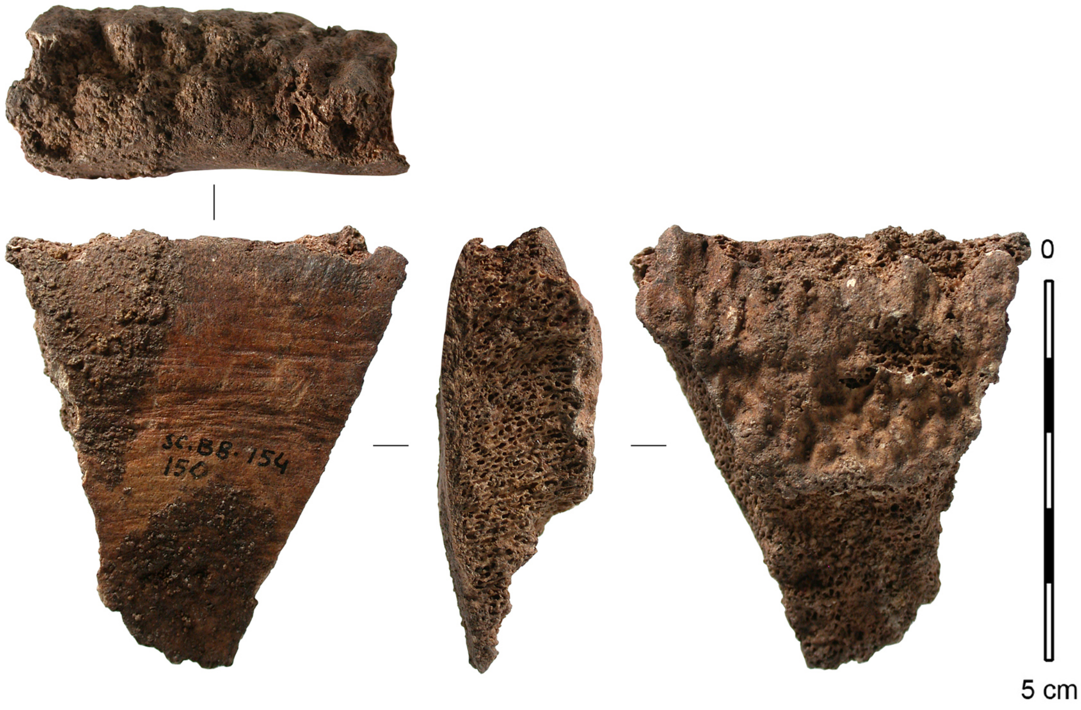

Supplementary Figure 85
Study number: SC B8 L31G 3932
Museum number: SC B8 L31G 3932
Site and layer: Santa Catalina III
Typology: unworked
ZooMS ID: Fin whale
^{14}C date: none
(scale 1:2)

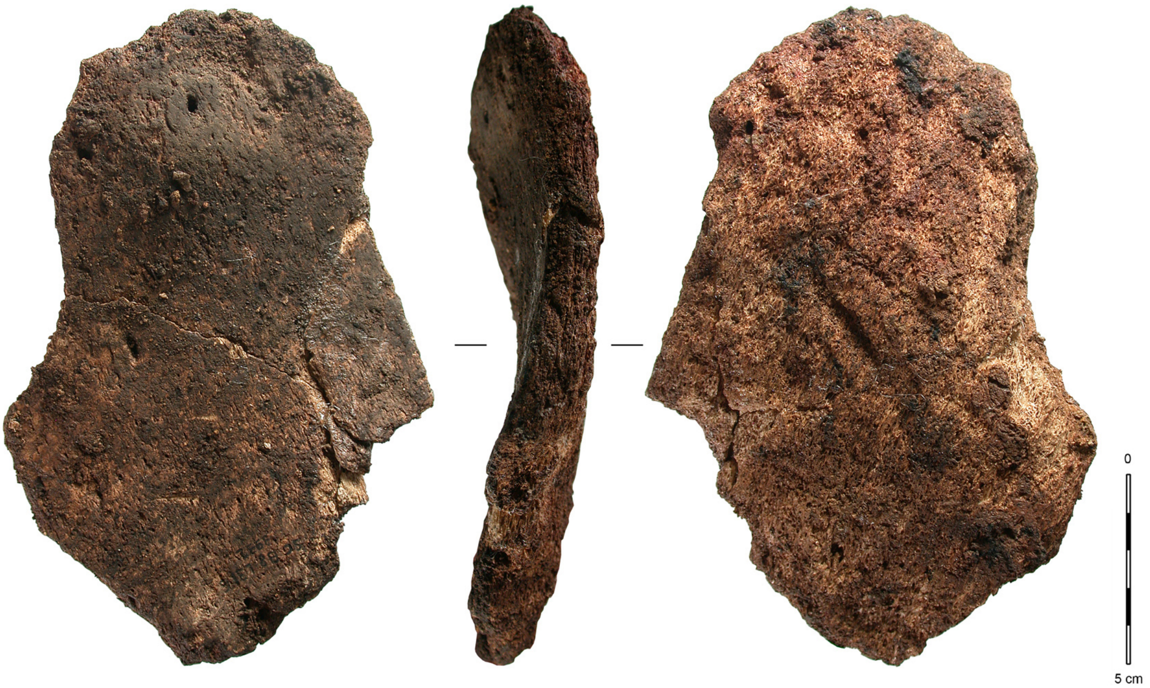

Supplementary Figure 86
Study number: SC B8 L31H 2251
Museum number: SC B8 L31H 2251
Site and layer: Santa Catalina III
Typology: unworked
ZooMS ID: Fin whale
^{14}C date: none
(photograph taken after sampling)

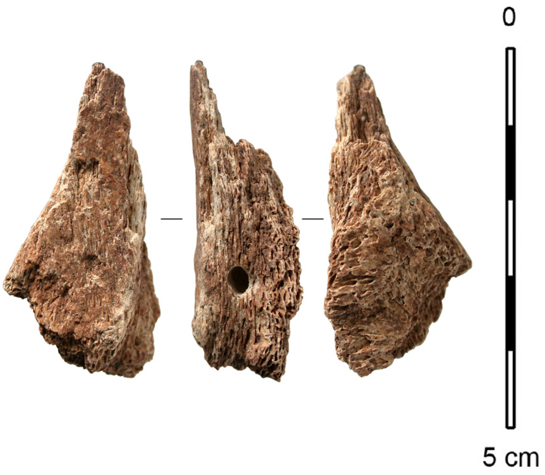

Supplementary Figure 87
Study number: SC C4 L29 229
Museum number: SC C4 L29 229
Site and layer: Santa Catalina II
Typology: blank?
ZooMS ID: Right whale / Bowhead whale
^{14}C date: none

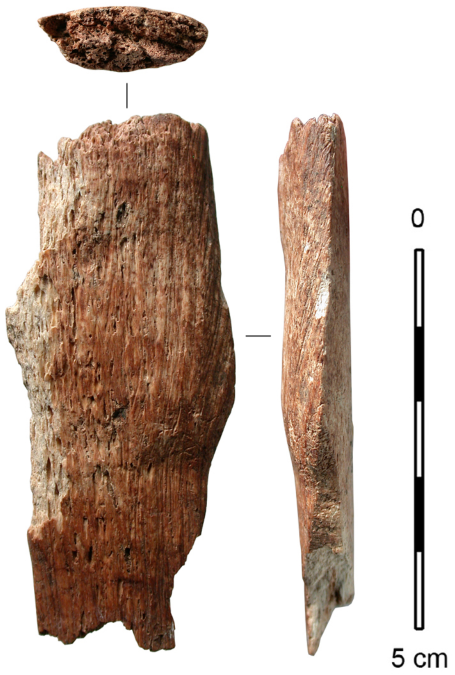

Supplementary Figure 88
Study number: SC C6 159 1
Museum number: SC C6 159 1
Site and layer: Santa Catalina III
Typology: unworked
ZooMS ID: Fin whale
^{14}C date: MUSE20042, 13060 ± 50 BP, 15699-15177 cal BP

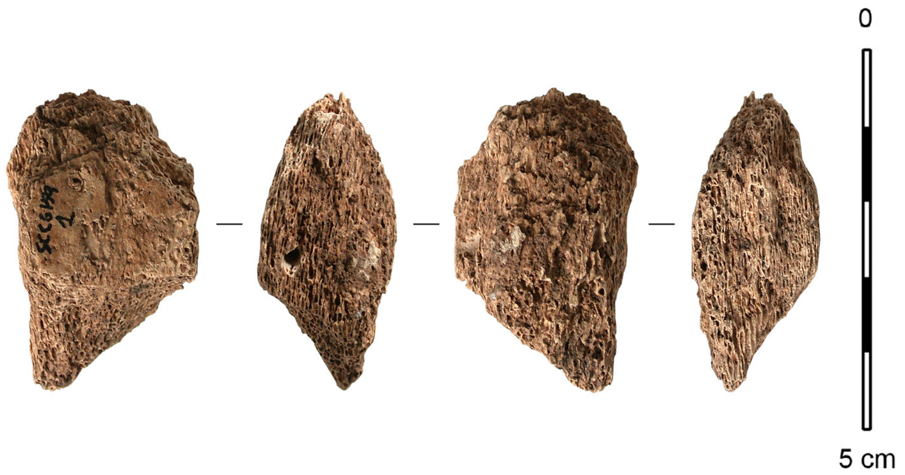

Supplementary Figure 89
Study number: SC C8 149 354
Museum number: SC C8 149 354
Site and layer: Santa Catalina **III**
Typology: unworked
ZooMS ID: Fin whale
^{14}C date: MUSE20074, 13060 ± 50 BP, 15699-15151 cal BP
(photograph taken after sampling)

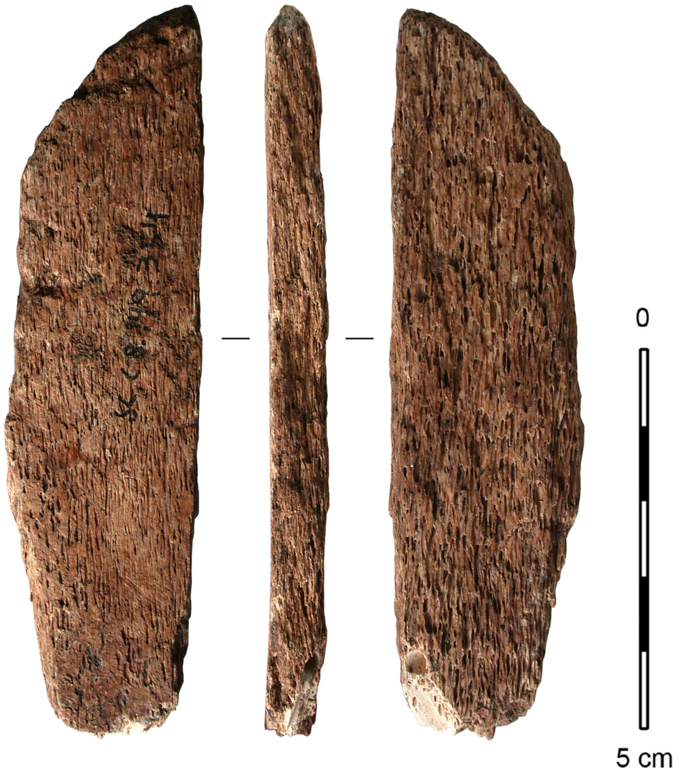

Supplementary Figure 90
Study number: SC C8 158 130
Museum number: SC C8 158 130
Site and layer: Santa Catalina **III**
Typology: unworked
ZooMS ID: Fin whale
^{14}C date: MUSE20128, 12810 ± 60 BP, 15390-14844 cal BP
(photograph taken after sampling)

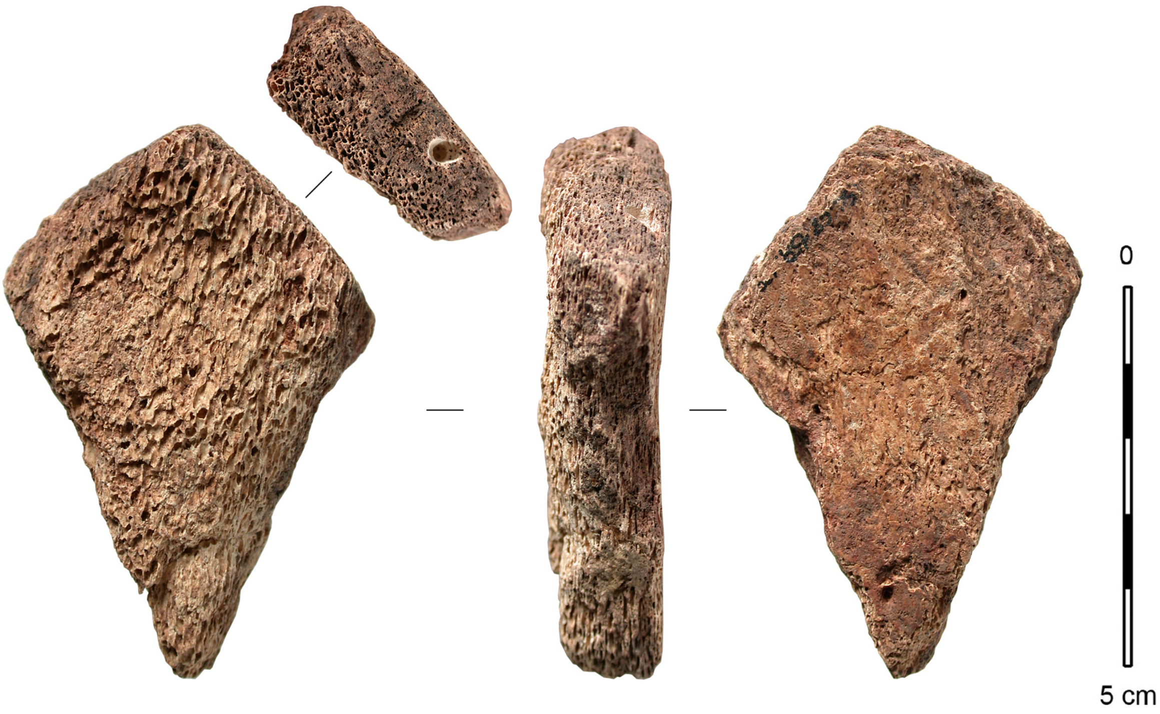

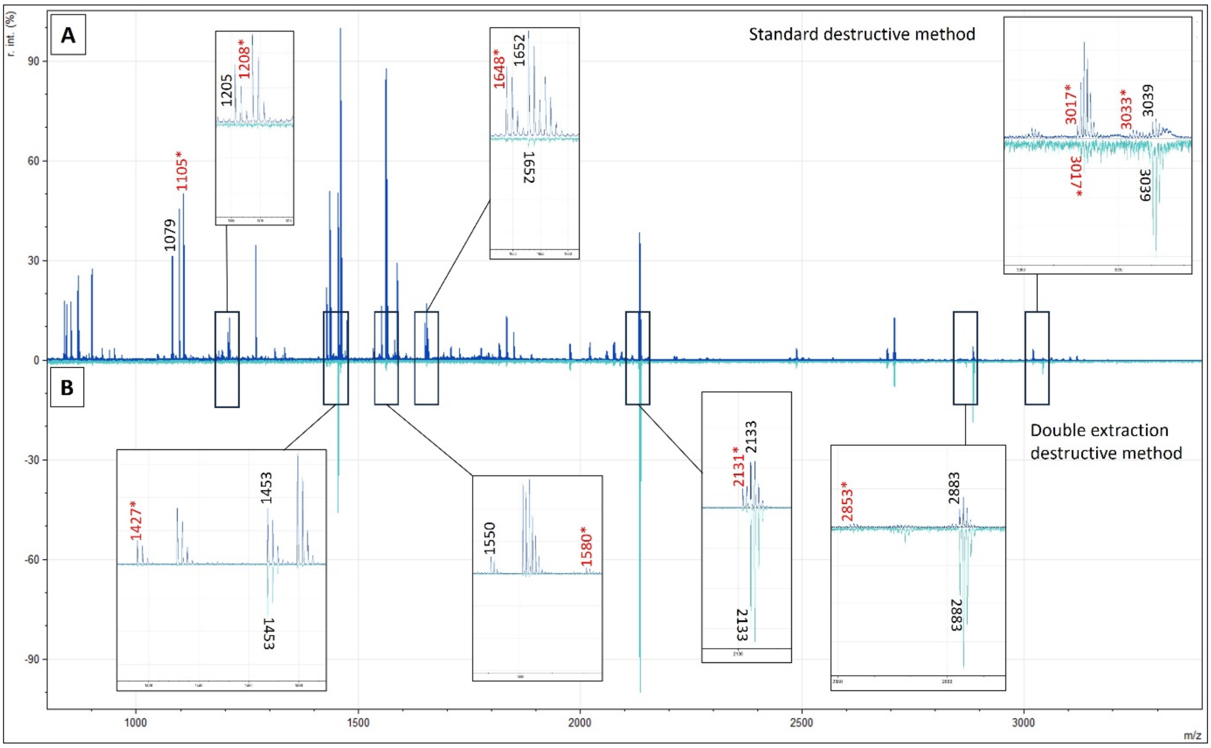

Supplementary Figure 91. MALDI-TOF-MS spectra for a cetacean bone artifact (a wedge, identifier 780, Supplementary Figure 22) from the site of Isturitz excavated by St-Périer. Top spectrum (A) shows the mixed whale and cattle markers obtained when the standard destructive extraction method was performed; lower spectrum (B) shows the whale markers and reduced cattle signal following the double extraction destructive method. Inset images provide detailed views of key peptide markers used for both cetaceans (in this case a sperm whale, black text) and cattle (red text with *). Spectrum intensity has been normalised for comparative purposes.

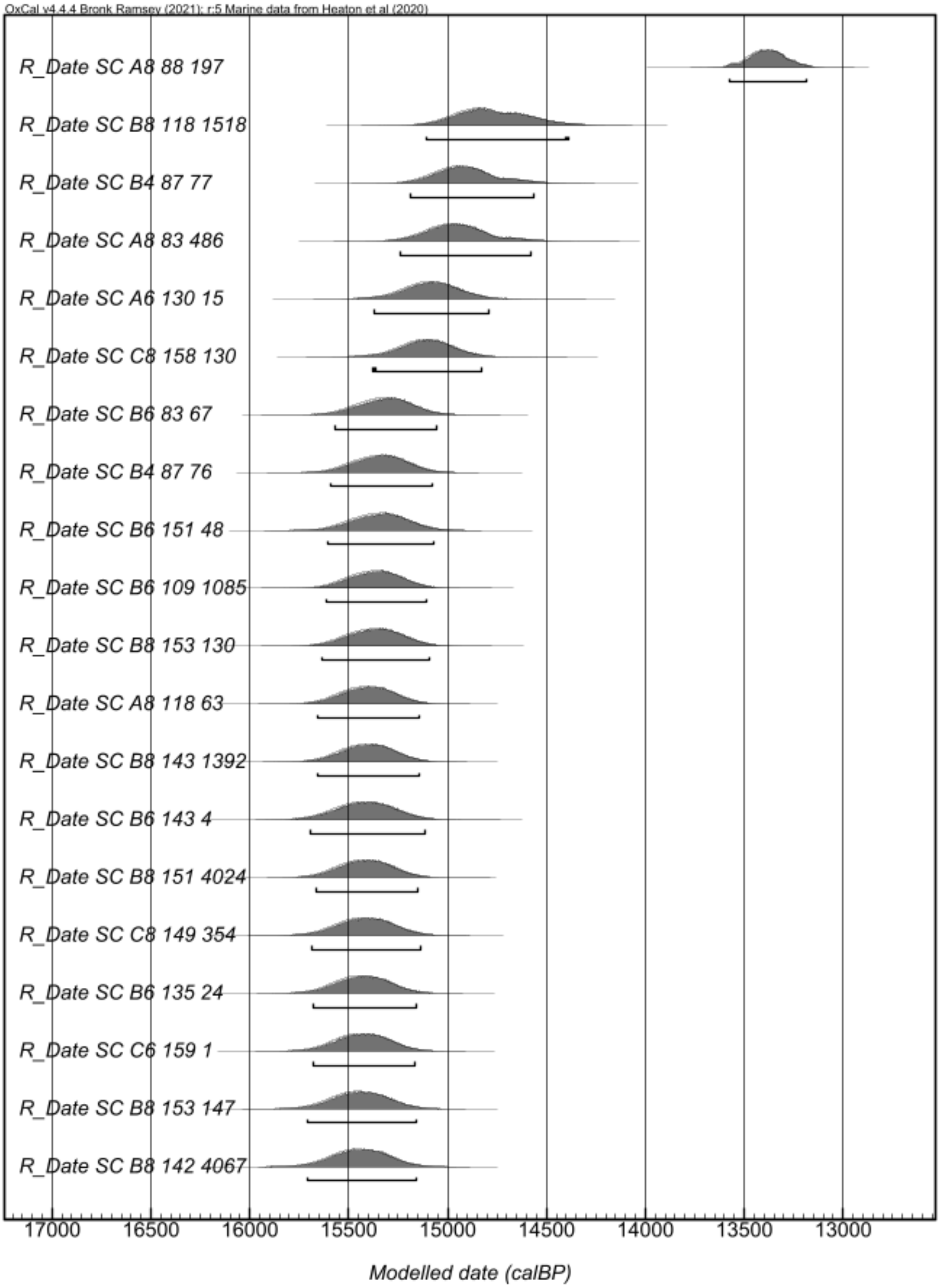

Supplementary Figure 92. Calibrated and reservoir corrected ^{14}C ages of the whale samples in OxCal 4.4 (Bronk Ramsey 2009), from youngest to oldest. Samples from the site of Santa Catalina (Part I). See Supplementary Data 1 for source data and more information on each sample, including species. See Supplementary Code 1 file for source code.

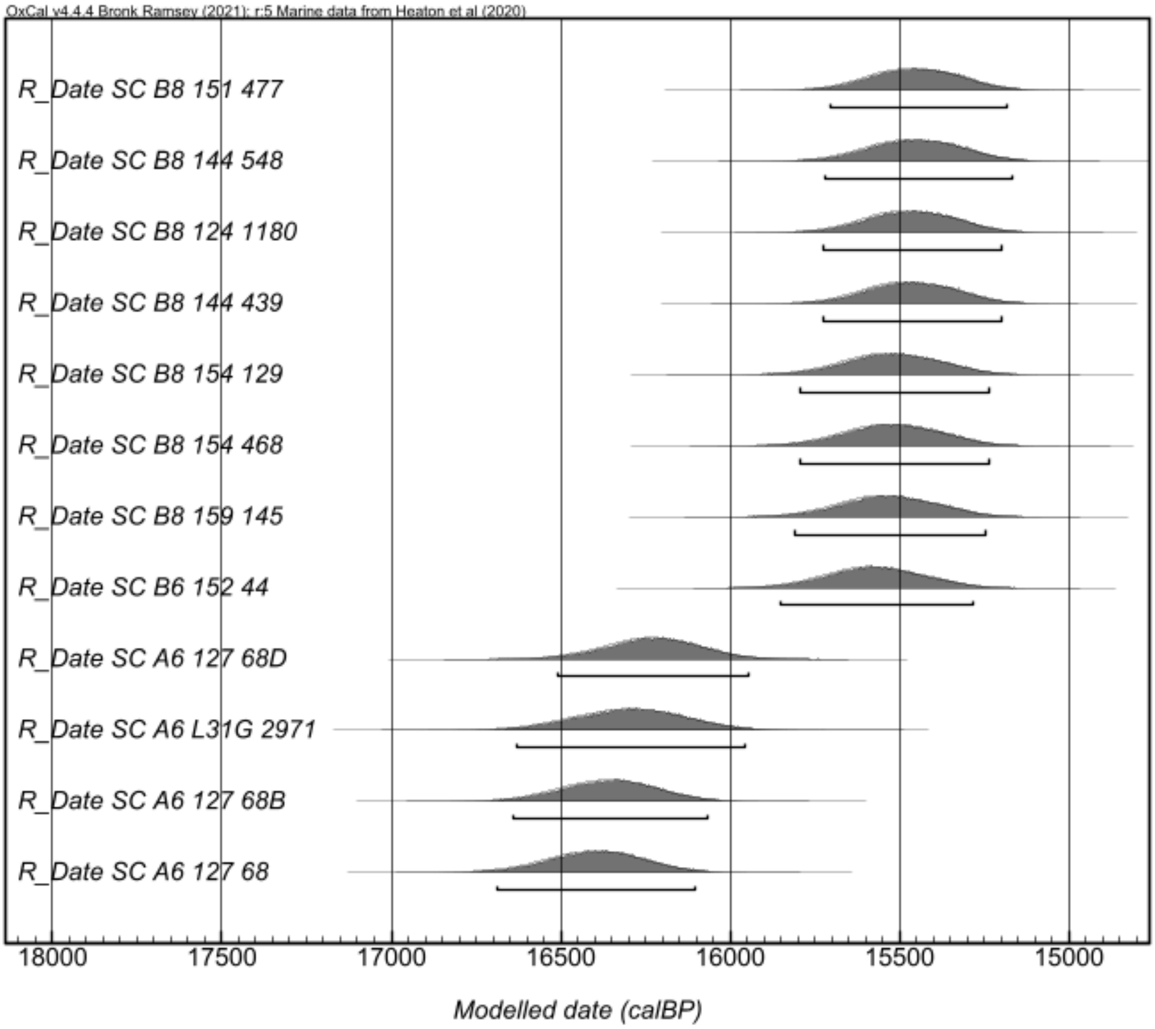

Supplementary Figure 93. Calibrated and reservoir corrected ^{14}C ages of the whale samples in OxCal 4.4 (Bronk Ramsey 2009), from youngest to oldest. Samples from the site of Santa Catalina (Part II). See Supplementary Data 1 for source data and more information on each sample, including species. See Supplementary Code 1 file for source code.

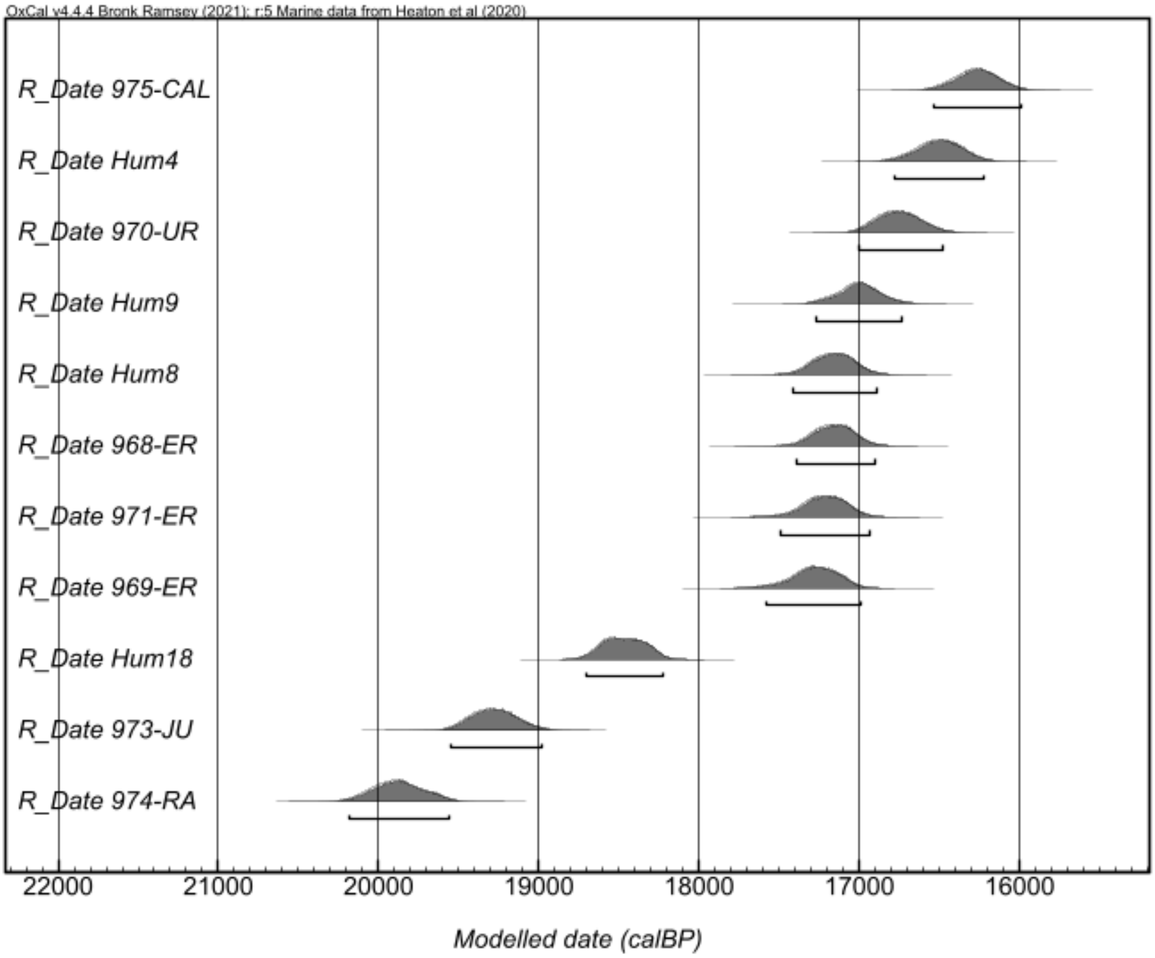

Supplementary Figure 94. Calibrated and reservoir corrected ^{14}C ages of the whale samples in OxCal 4.4 (Bronk Ramsey 2009), from youngest to oldest. Samples from the Cantabrian sites. See Supplementary Data 1 for source data and more information on each sample, including species. See Supplementary Code 1 file for source code.

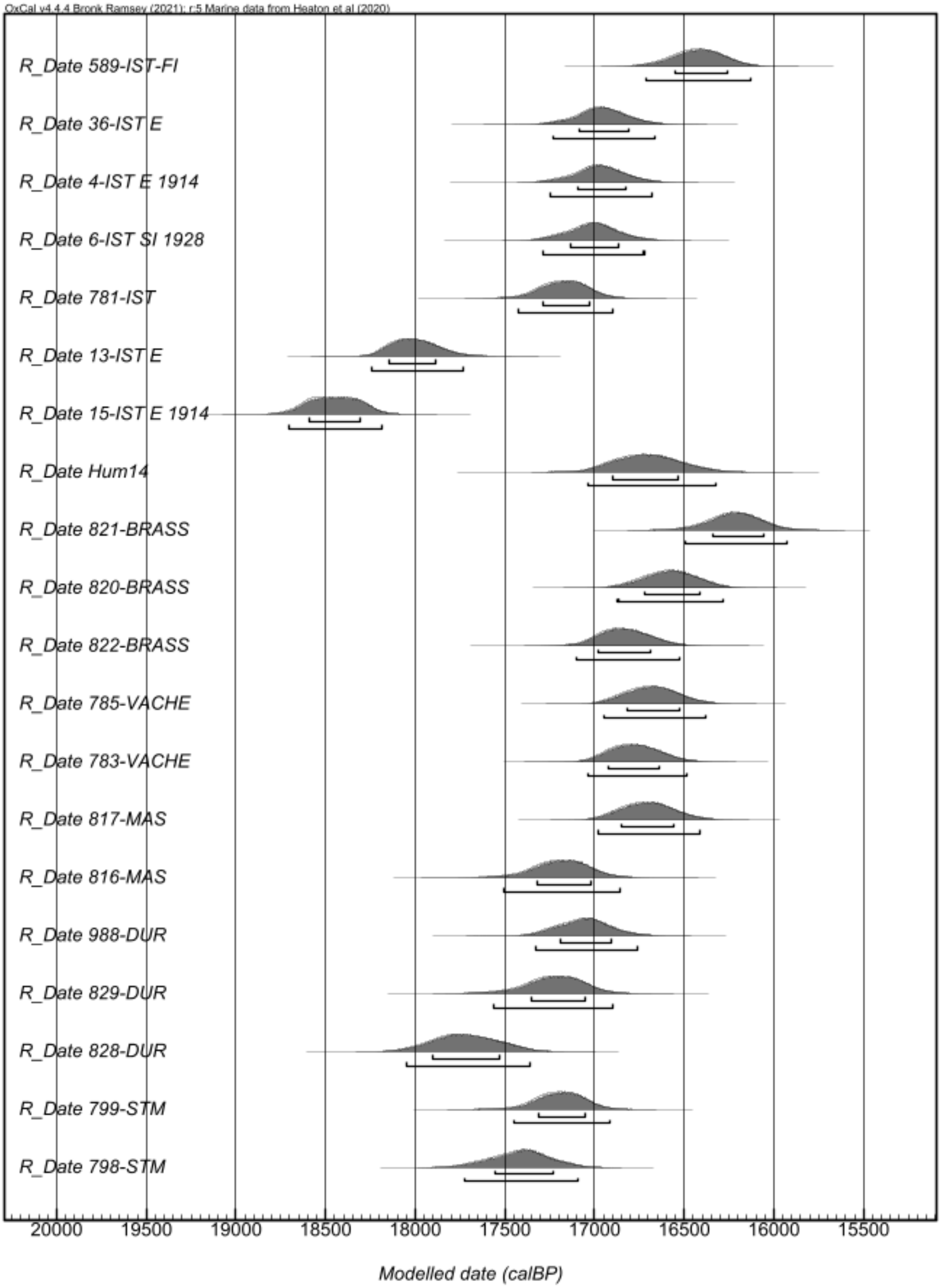

Supplementary Figure 95. Calibrated and reservoir corrected ^{14}C ages of the whale samples in OxCal 4.4 (Bronk Ramsey 2009), from youngest to oldest. Samples from the French sites. See Supplementary Data 1 for source data and more information on each sample, including species. See Supplementary Code 1 file for source code.

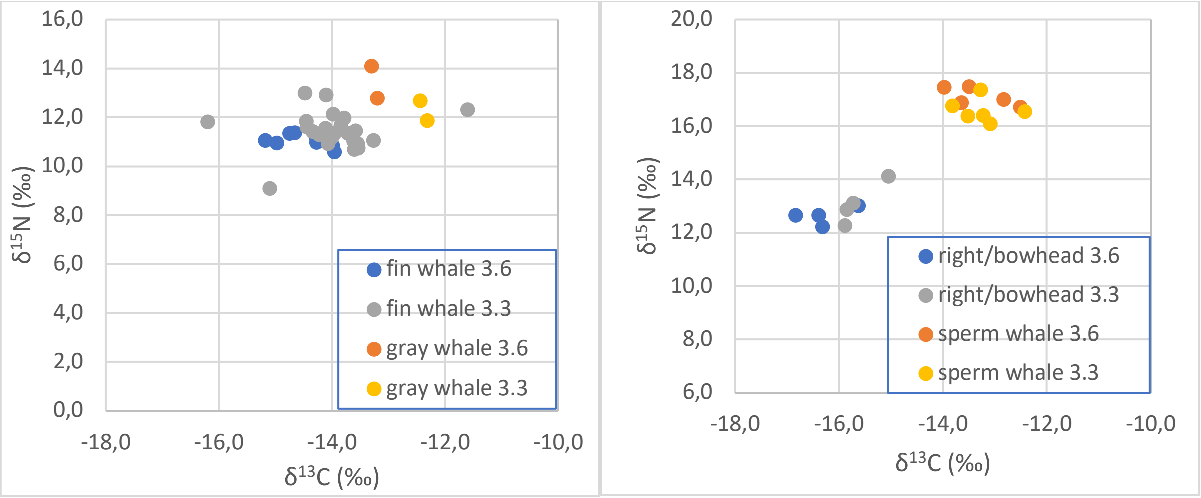

Supplementary Figure 96. $\delta^{13}C$ and $\delta^{15}N$ ratios of Paleolithic whales (this study), showing the differences between the different atomic CN ratios per species. Number of specimens: fin whale = 31; gray whale = 4; right/bowhead whale = 8; sperm whale = 11. See Supplementary Data 4, tab "PaleoCet material", for source data.

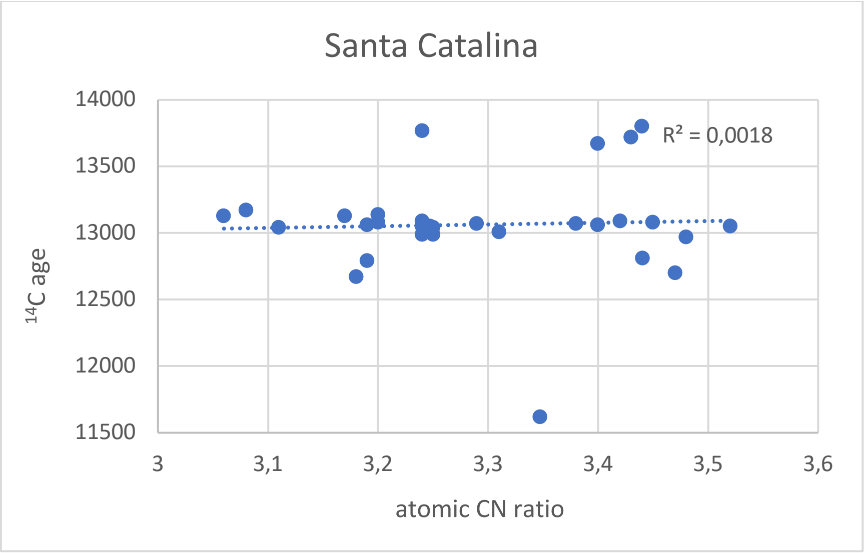

Supplementary Figure 97. ^{14}C ages plotted against the atomic CN ratio of material from the site of Santa Catalina. A correlation between the two might indicate contamination, which is not the case. Number of specimens = 30. See Supplementary Data 4, tab "PaleoCet material", for source data.

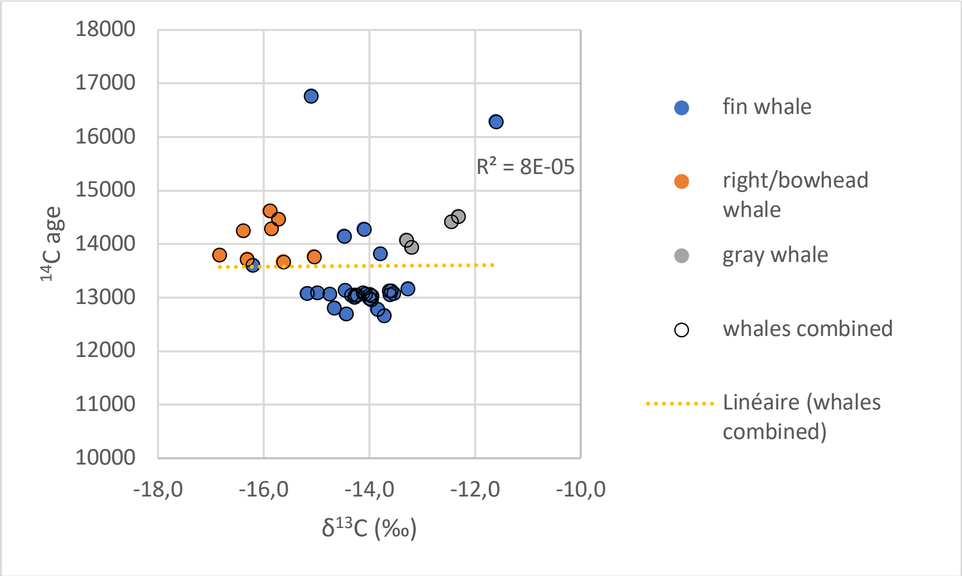

Supplementary Figure 98. Relationship between δ13C ratios and conventional 14C ages among the analyzed bones of fin, gray and right/bowhead whales. Number of specimens: fin whale = 31; gray whale = 4; right/bowhead whale = 8. See Supplementary Data 4, tab "PaleoCet material", for source data.

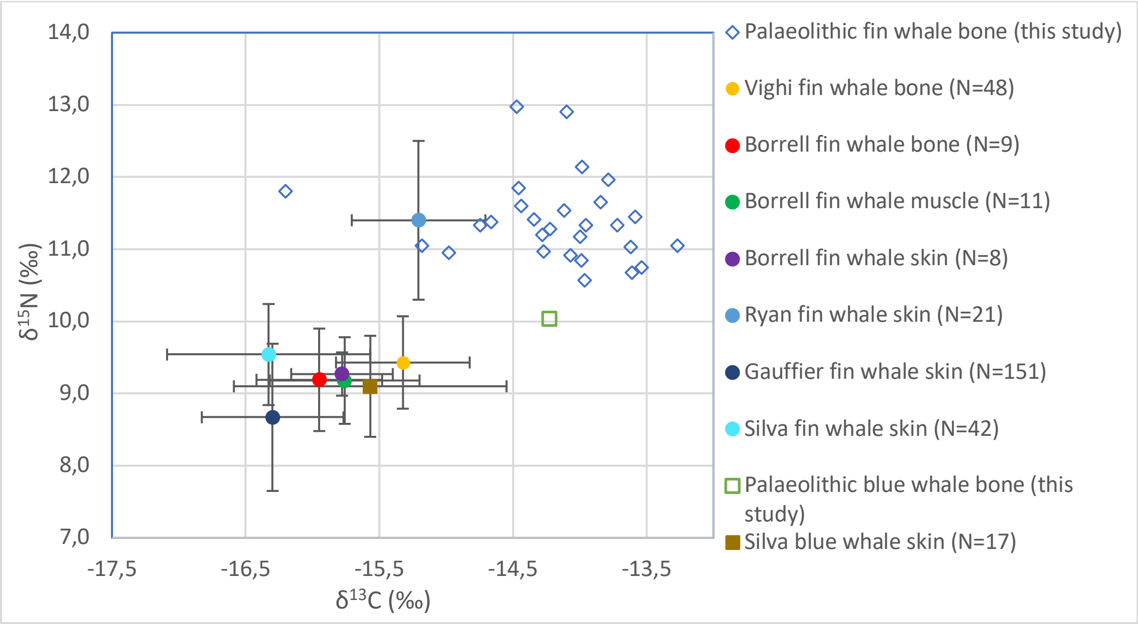

Supplementary Figure 99. δ13C and δ15N ratios from the studied Paleolithic fin whales and blue whale compared with stable isotope values from modern whales from the Atlantic Ocean (after applying Suess effect and, if applicable, tissue corrections)[1,2,3,4,5]. Whiskers represent errors bars on average values. See Supplementary Data 4 for source data.

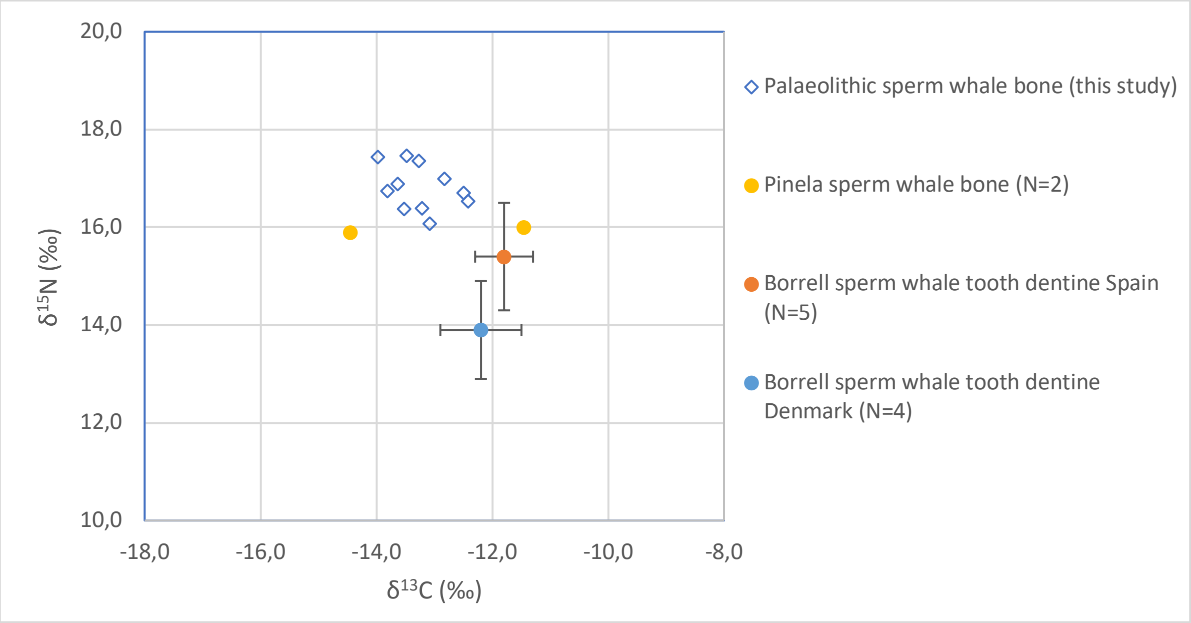

Supplementary Figure 100. $\delta^{13}C$ and $\delta^{15}N$ ratios from the studied Paleolithic sperm whales compared with stable isotope values from modern whales from the Atlantic[6,1] (after applying Suess effect and, if applicable, tissue corrections). Whiskers represent errors bars on average values. See Supplementary Data 4 for source data.

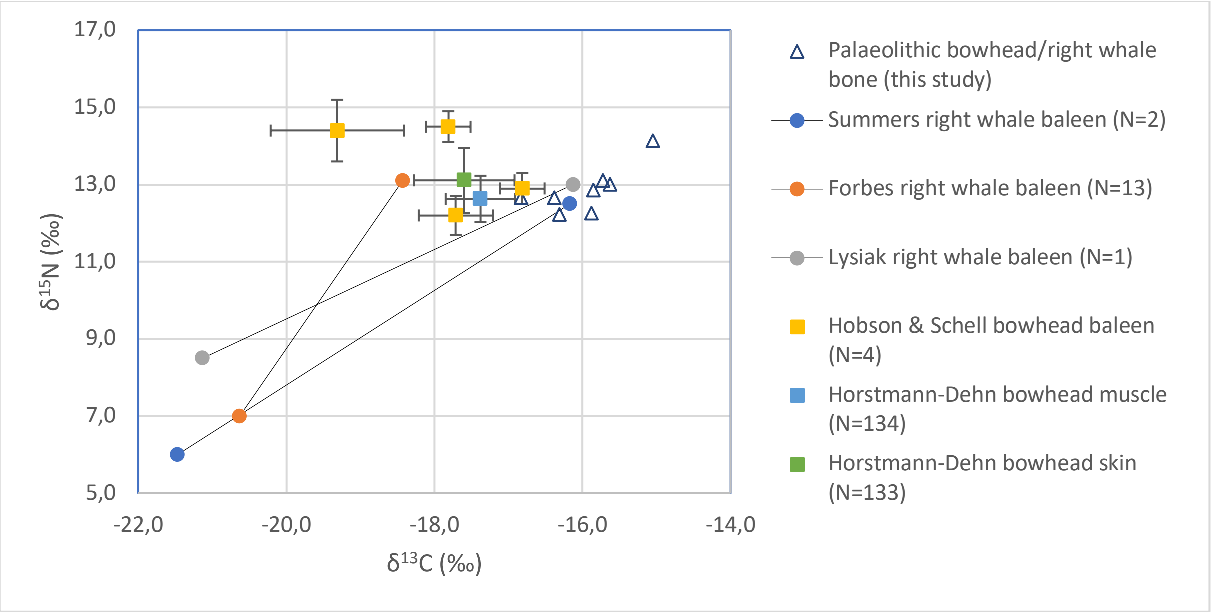

Supplementary Figure 101. $\delta^{13}C$ and $\delta^{15}N$ ratios from the studied Paleolithic bowhead/north Atlantic right whales compared with stable isotope values from modern whales from the Atlantic Ocean (right whales)[7,8,9] and Canadian Arctic (bowhead whales)[10,11] (after applying Suess effect and, if applicable, tissue corrections). Whiskers represent errors bars on average values, while values from Summers, Forbes and Lysiak present the observed range, connecting the lowest and highest values with a thin line (averages were not available). See Supplementary Data 4 for source data.

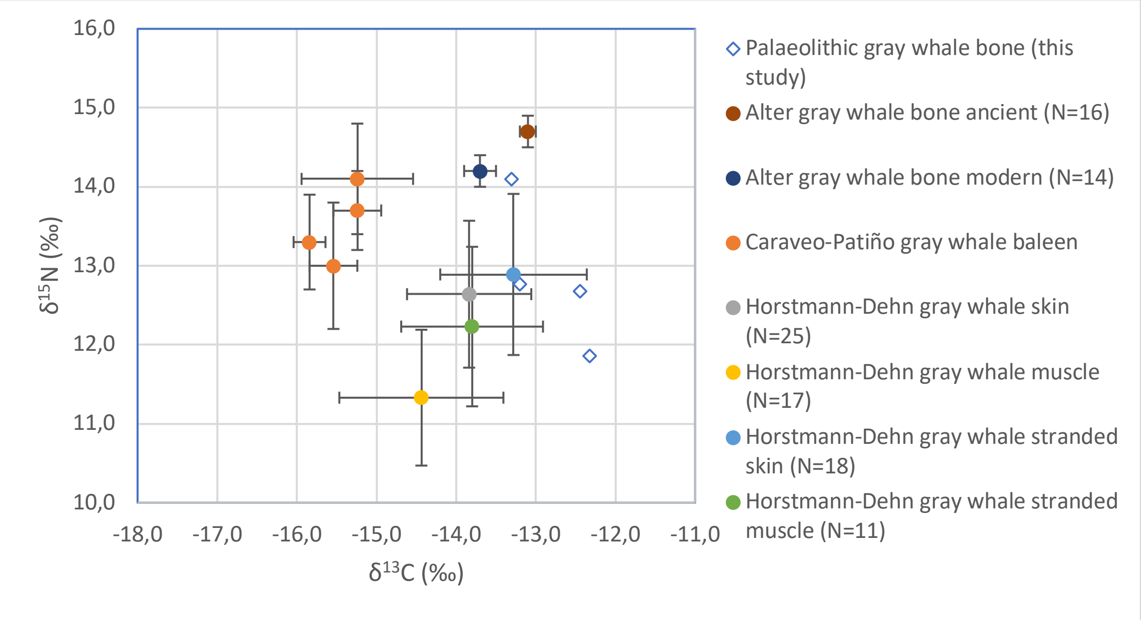

Supplementary Figure 102. $\delta^{13}C$ and $\delta^{15}N$ ratios from the studied Paleolithic gray whales compared with stable isotope values from ancient gray whales[12], modern whales from the Pacific Ocean[13,11] (after applying Suess effect and, if applicable, tissue corrections). Whiskers represent errors bars on average values. See Supplementary Data 4 for source data.

Supplementary Discussion: Comparing stable isotope signatures in Paleolithic versus modern whales

We compared the stable isotope ratios obtained for samples of Paleolithic whales in our dataset with ratios reported in the literature for modern counterparts. Teixeira et al.[14] outline useful guidelines for stable isotope analysis in cetacean research, underlining especially the difficulties when comparing data obtained using different tissues (muscle/skin/baleen/bone), the need for a Suess effect correction, tissue pretreatment (lipid removal), and considering intra- and inter-individual variation. We discuss below how we took into account each of these points.

Tissue corrections

Numerous previous studies applied stable isotope analysis to modern whale tissues, although typically on muscle, skin or baleen. A tissue correction is needed for making these values comparable with bone collagen stable isotope data. Schoeninger and Moore[15] report that the offset between muscle and bone collagen in $\delta^{13}C$ ratios is circa 2‰[16,17]. Fischer et al.[18] analyzed carbon and nitrogen stable isotope ratios on bone collagen and flesh of modern fish in Denmark, finding flesh-bone offsets of 2.1 ± 0.6‰ for $\delta^{13}C$ ratios and -0.6 ± 0.4‰ for $\delta^{15}N$ ratios. Borrell et al.[1] analyzed the stable isotope ratios of various tissues from fin whales caught between 1983 and 1985 from the Caneliñas whaling station off the coast of Northwest Spain. For bone protein from 9 individuals they found averages of $\delta^{13}C$ = -16.46 ± 0.47‰ and $\delta^{15}N$= 9.19 ± 0.71‰, and for muscle from 11 individuals of $\delta^{13}C$ = -18.27 ± 0.56‰ and $\delta^{15}N$= 9.88 ± 0.58‰, and skin values of $\delta^{13}C$ = -18.29 ± 0.38‰ and $\delta^{15}N$= 9.97 ± 0.30‰. This gives a muscle-bone offset of 1.81‰ for $\delta^{13}C$ ratios and -0.69 for $\delta^{15}N$ ratios and for skin-bone 1.83‰ for $\delta^{13}C$ ratios and -0.79 for $\delta^{15}N$ ratios. Based on these values from the literature, we opted for the corrections for both muscle-bone and skin-bone of 2‰ for $\delta^{13}C$ ratios and of -0.7‰ for $\delta^{15}N$ ratios.

Baleen is constructed of highly mineralized keratin[19], which is similar to bone collagen or dentine in composition, and therefore we considered that results of stable isotope analyses from baleen are directly comparable to those obtained from bone. While bone remodels and provides an average of many years, baleen plates grow continuously at the base whilst being eroded away at the tip[20]. Baleen tissue thus represents an archive of information about the animal's life history from the last 2 (gray whale[13]) to 20 years (bowhead whale[21]), depending on species, baleen length and growth rates, making it a very useful tissue to examine changes in foraging through fine time scales such as seasons.

Suess Effect correction

Comparing modern samples with archaeological or historic samples requires a correction to the $\delta^{13}C$ ratios for the Suess Effect, which involves the incorporation of largely depleted carbon into the atmosphere through the burning of fossil fuels since the industrial revolution[22]. We used the SuessR package[23] to calculate the correction needed based on the region and year of sample collection (Supplementary Data 4).

Tissue pretreatment

Bas et al.[24] examined the combined effect of delipidation and demineralization on the stable isotope results of sea lion and dusky dolphin bones. Their samples were either left untreated or were treated with a chloroform and methanol solution, followed by demineralization with HCl acid. They found atomic CN ratios above 3.6 for untreated samples, indicating the presence of lipids and/or carbonates. Compared with pretreated samples, all untreated samples had lower $\delta^{13}C$ (differences were ranging from 0.3 to 2 ‰), while this varied for $\delta^{15}N$ ratios (differences were ranging from +0.2 to -1.3 ‰). Das et al.[25] had the same findings for $\delta^{13}C$ ratios on skin samples. All skin and muscle samples in the literature used for this comparison applied a lipid removal step but no demineralization step. Baleen samples were often subjected to lipid removal (3 out of 5) but not to demineralization. Bone and tooth dentine were treated in a variety of ways in the literature, precluding the application of a systematic correction.

Intra- and inter-individual variation

Smith et al.[26] investigated intra-skeletal stable isotope variability in 72 cetacean skeletons from 14 species. They found that for 84.7% of the individuals there was >1‰ variability in $\delta^{13}C$ ratios, and for 55.5% >2‰ variability. Regarding $\delta^{15}N$ ratios, 59.7% of the individuals had >1‰ and 15.3% >2‰ variability. The authors assume that variation >1‰ in $\delta^{13}C$ ratios and >2‰ in $\delta^{15}N$ ratios reflects changes in foraging behavior. Borrell et al.[1] analyzed muscle tissues of a large number of individuals (n=65) of fin whale, finding no significant age-related variation (4-65 years), nor significant sex-related differences (n=55 males, n=10 females) in stable isotope values, deducing that this absence of individual variation reflects a consistent use of the same feeding grounds and a similar diet (almost exclusively krill) by different age groups and sexes. Muscle tissue from bowheads in the eastern Arctic showed no age-related isotopic differences, while their baleen revealed that subadult whales feed slightly more in nearshore waters, resulting in lower $\delta^{13}C$ ratios, compared with adults in deeper waters[27]. A significant negative correlation was found between age and $\delta^{15}N$ ratios for bowheads, with fetuses and juveniles having higher $\delta^{15}N$ ratios than adults, which is most likely due to milk consumption[11]. It was not possible to take intra- or inter-individual stable isotope variability into account in our analysis.

Fin whales

We contrasted stable isotope ratios obtained from our Paleolithic samples of fin whales (n = 30) with values obtained from modern fin whales in the eastern North Atlantic (after applying Suess effect corrections and, when necessary, tissue corrections) originating from bone samples (9 individuals[1] + 48 individuals[3]), muscle samples (11 individuals[1]) and skin samples (8 individuals[1] + 21 individuals[2] + 42 individuals[4] + 151 individuals[5]).

Fin whales in our sample generally display high $\delta^{15}N$ ratios when compared with their modern counterparts (Supplementary Figure 99), indicating that they occupied on average a higher trophic level than today's whales[28], or that the $\delta^{15}N$ baseline has significantly changed over time. When compared with the other whale species in our study, modern fin whales are considered a relative generalist predator. In the North Atlantic, their diet is dominated by zooplankton (including multiple euphausiid species) but can also include non-negligible contributions of small pelagic fishes (capelin, *Mallotus villosus*; Atlantic herring, *Clupea harengus*; sandlance, *Ammodytes* spp.[29]). Feeding strategies can vary across populations, among individuals of the same population, and over time in response to environmental change. For example, an analysis of stable isotope ratios in skin and blubber of 99 fin whales in the Saint Lawrence Estuary over 1998-2006[29] found that the population was initially dominated by two types of specialists (individuals focused either on krill or on lipid-rich pelagic fishes). It then shifted toward one composed either of krill specialists or (for about 66% of the individuals) true generalists feeding on various zooplankton and fish prey, a change that coincided with a decrease in biomass of their predominant prey, Arctic krill (*Thysanoessa* spp.). Previous studies also found variation in $\delta^{15}N$ ratios in baleen and muscle samples that reflect seasonal variation in feeding strategies of these highly migratory species: Aguilar et al.[30] (using part of the same dataset as Borrell et al.[1] but only looking at males) found higher $\delta^{15}N$ ratios during periods of intense feeding (at high latitudes), that decreased during fasting (migration and calving seasons), whereas Rita et al.[31] found that $\delta^{15}N$ ratios were higher in winter than in summer. Stable isotope ratios obtained from bones reflect the integration of feeding strategies over years. Accordingly, the relatively high $\delta^{15}N$ ratios in Paleolithic samples could indicate that on average fin whales' diets integrated relatively high fractions of fish when compared to today's whales, which may have been related to the different environmental circumstances.

The Paleolithic whales also exhibit high $\delta^{13}C$ ratios when compared with modern counterparts (Supplementary Figure 99). $\delta^{13}C$ ratios in marine waters vary geographically, with a tendency to decline from coastal and benthic areas to offshore and pelagic areas, and towards polar latitudes[32]. The higher $\delta^{13}C$ ratios in ancient whales may thus indicate a tendency to rely more on coastal and/or on more tropical and subtropical areas than today's populations.

Blue whales

We contrasted stable isotope ratios of the single blue whale bone sample in our study with skin from 17 blue whale individuals analyzed by Silva et al.[4], corresponding to blue whales migrating through the Azores and likely reflecting winter and spring feeding grounds in the Northwest African upwelling and pelagic tropical/subtropical regions. The ancient blue whale shows higher δ13C and δ15N values than its modern counterparts, and isotopically appears to fall in between the modern and ancient fin whales (Supplementary Figure 99). Modern blue whales are considered dietary specialists, feeding almost exclusively on krill (*Meganyctiphanes norvegica*, *Thysanoessa raschii* and *T. inermis*)[4]. They mostly feed in high-latitude summer grounds, and fast during migration to and from low latitude winter calving areas, but there is evidence of year-round feeding[33].

Sperm whales

We contrasted stable isotope ratios obtained from our Paleolithic bone samples of sperm whales (n = 11) with values obtained from modern sperm whales from the Atlantic (2 bone samples[6] + 9 tooth dentine samples[1]) after applying tissue and Suess effect corrections.

We found overlapping ranges of $\delta^{13}C$ ratios between modern and ancient whales, with the latter falling on the higher end of the $\delta^{15}N$ ratios range in relation to the former (Supplementary Figure 100). Sperm whales are dominant predators of the mesopelagic ocean, preying on many of the larger meso- and bathy-pelagic species of the deep ocean, including cephalopods and fish[34]. Modern sperm whales in the North Atlantic are highly structured by sex and age, with females and young whales found in tropical and temperate waters whereas older males progressively move towards higher latitudes (migrating periodically southwards for reproduction)[34,35]. Both males and females are currently found off NW Spain[35], but it is plausible that in the cooler Paleolithic oceans the area would have been dominated by older males and this could perhaps explain the higher $\delta^{15}N$ ratios in Paleolithic samples. Indeed, previous studies found that $\delta^{15}N$ ratios (measured in tooth dentine) tend to increase with age in males, attributed to the increasing strength and diving capacity that individuals acquire when growing old, which allow them to progressively feed on bigger prey (mostly giant squids) at higher trophic levels[35]. In contrast, an opposite trend (or no age effect) has been observed for females, possibly explained by a tendency to reduce the depth of dives (thus a shift to shallower and smaller prey) during pregnancy and lactation[35].

Right/bowhead whales

As we were unable to distinguish between bowhead whale and North Atlantic right whale through ZooMS, we compared the stable isotope signatures of Paleolithic samples identified as one of these species (n=8) with those of modern North Atlantic right whales (baleen of two individuals analyzed by Summers et al.[7]; 13 by Forbes et al.[9]; and one by Lysiak et al.[8]) and bowheads from the Canadian Artic (baleen from 4 individuals from Hobson and Schell[10]; muscle from 134 individuals and skin from 133 individuals from Horstmann-Dehn et al.[12]).

Modern bowhead whales and right whales are specialists that feed mainly on calanoid copepod species (most commonly, and respectively, *Calanus hyperboreus* and *Calanus finmarchicus*), but they can also feed on other mesozooplancton species at a higher trophic level (e.g. mysids, euphausiids, chaetognaths)[34,36]. Due to their distinct feeding habitats at certain latitudes, the species' overlap in $\delta^{13}C$ ratios is somewhat unexpected.

Modern right whales tend to have overall lower values of $\delta^{13}C$ and $\delta^{15}N$ ratios than modern bowhead whales, and the stable isotope signatures of Pleistocene bones overlap better with those of the later (Supplementary Figure 101). As the published studies investigating modern right whales observed stable isotopic ranges rather than averages with standard deviations, these are displayed here as such (Supplementary Figure 101). It is important to note that these ranges[7,8,9] cover the stable isotope value extremes (winter vs. summer feeding) and when averaged, they would most likely display lower $\delta^{13}C$ and $\delta^{15}N$ ratios compared with the bowhead data. Despite the partial overlap between the modern samples of both species precluding a more certain species identification of the Paleolithic bone samples, the ancient samples appear more likely to be bowheads than right whales. Although fin whales are often known to forage at a slightly higher trophic level relative to Balaenidae, when comparing modern fin whales (Supplementary Figure 99) and modern bowhead and North Atlantic right whales (Supplementary Figure 101), we found that fin whales also have lower $\delta^{15}N$ ratios than bowhead whales and a similar range to right whales. This pattern is not only evident among ancie
nt whale samples but also in modern whales and can probably be explained by their different migration styles (coastal vs open ocean) and summer feeding grounds characterized by different baseline $\delta^{15}N$ values.

Gray whales

As gray whales are currently extinct in the Atlantic, we could only compare our ancient specimens (n=4) with ancient gray whale from the Pacific (16 bone samples from 150-2690 yr BP[12]) and modern gray whales from the Pacific (14 bone samples[12], baleen from 4 individuals[13], 25 skin and 17 muscle samples from Alaskan individuals, as well as 18 skin and 11 muscle samples from individuals stranded in California[11]) (Supplementary Figure 102). Any differences in $\delta^{13}C$ ratios may be due to the large geographic distance, although the $\delta^{15}N$ ratios overlap considerably, potentially indicating similar foraging preferences. Modern Pacific gray whales are highly coastal, feeding in shallow Artic and subarctic benthic environments, primarily on tube-dwelling ampeliscid amphipods, polychaete worms and bivalves[34].

Supplementary references: references cited in the captions of Supplementary Figures 99 through 102, or in the Supplementary Discussion.

1. Borrell, A., Abad-Oliva, N., Gómez-Campos, E., Giménez, J., Aguilar, A. 2012. Discrimination of stable isotopes in fin whale tissues and application to diet assessment in cetaceans, *Rapid Communications in Mass Spectrom*etry 26, 1596–1602.

2. Ryan, C., Berrow, S. D., McHugh, B., O'Donnell, C., Trueman, C. N., O'Connor, I. 2013. Prey preferences of sympatric fin (*Balaenoptera physalus*) and humpback (*Megaptera novaeangliae*) whales revealed by stable isotope mixing models. *Marine Mammal Science* 30(1), 242-258. https://doi.org/10.1111/mms.12034

3. Vighi, M., Borrell, A., Aguilar, A., 2016. Stable isotope analysis and fin whale subpopulation structure in the eastern North Atlantic. *Marine Mammal Science* 32(2), 535–551. https://doi.org/10.1111/mms.12283

4. Silva, M. A., Borrell, A., Prieto, R., Gauffier, P., Bérubé, M., Palsbøl, P.J., Colaço, A. 2019. Stable isotopes reveal winter feeding in different habitats in blue, fin and sei whales migrating through the Azores. *Royal Society Open Science* 6, 181800. https://doi.org/10.1098/rsos.181800

5. Gauffier, P., Borrell, A., Silva, M. A., Víkingsson, G. A., López, A., Giménez, J., Colaço, A., Halldórsson, S. D., Vighi, M., Prieto, R., de Stephanis, R., Aguilar, A. 2020. Wait your turn, North Atlantic fin whales share a common feeding ground sequentially. *Marine Environmental Research* 155, 104884. https://doi.org/10.1016/j.marenvres.2020.104884

6. Pinela, A. M., Borrell, A., Cardona, L., Aguilar, A. 2010. Stable isotope analysis reveals habitat partitioning among marine mammals off the NW African coast and unique trophic niches for two globally threatened species. *Marine Ecology Progress Series* 416, 295–306. https://doi.org/10.3354/meps08790

7. Summers, E. L., Estrada, J. A., Zeeman, S. I. 2006. A note on geographic and seasonal fluctuations in the isotopic composition of baleen in four North Atlantic right whales (*Eubalaena glacialis*), *Journal of Cetacean Research and Management* 8(3), 241–245.

8. Lysiak, N. S. J., Trumble, S. J., Knowlton, A. R., Moore, M. J. 2018. Characterizing the Duration and Severity of Fishing Gear Entanglement on a North Atlantic Right Whale (Eubalaena glacialis) Using Stable Isotopes, Steroid and Thyroid Hormones in Baleen. *Frontiers in Marine Science* 5, 168. https://doi.org/10.3389/fmars.2018.00168

9. Forbes, R., Nakamoto, B., Lysiak, N., Wimmer, T., Hayden, B. 2023. Stable isotope analysis of baleen from North Atlantic right whales *Eubalaena glacialis* reflects distribution shift to the Gulf of St. Lawrence. *Marine Ecology Progress Series* 722, 177–193. https://doi.org/10.3354/meps14428

10. Hobson, K. A., Schell, D. M. 1998. Stable carbon and nitrogen isotope patterns in baleen from eastern Arctic bowhead whales (*Balaena mysticetus*). *Canadian Journal of Fisheries and Aquatic Sciences* 55, 2601–2607.

11. Horstmann-Dehn, L., Follmann, E. H., Rosa, C., Zelensky, G., George, C. 2012. Stable carbon and nitrogen isotope ratios in muscle and epidermis of arctic whales, *Marine Mammal Science* 28(2), E173–E190. https://doi.org/10.1111/j.1748-7692.2011.00503.x

12. Alter, S. E., Newsome, S. D., Palumbi, S. R. 2012. Pre-whaling genetic diversity and population ecology in eastern Pacific gray whales: insights from ancient DNA and stable isotopes. *PLoS ONE* 7, e35039. https://doi.org/10.1371/journal.pone.0035039.g006

13. Caraveo-Patiño, J., Hobson, K. A., Soto, L. A. 2007. Feeding ecology of gray whales inferred from stable-carbon and nitrogen isotopic analysis of baleen plates. *Hydrobiologia* 586, 17–25. https://doi.org/10.1007/s10750-006-0477-5

14. Teixeira, C. R., Troina, G. C., Daura-Jorge, F. G., Simões-Lopes, P. C., Botta, S. 2022. A practical guide on stable isotope analysis for cetacean research. *Marine Mammal Science* 38(3), 1200-1228.

15. Schoeninger, M., Moore, K. 1992. Bone stable isotope studies in archaeology. *Journal of World Prehistory* 6(2), 247-296.

16. Medaglia, C. C., Little, E. A., Schoeninger, M. J. 1990. Late Woodland diet on Nantucket Island: A study using stable isotope ratios. *Bulletin of the Massachusetts Archaeological Society* 51, 49–60.

17. van der Merwe, N. J. 1989. Natural variation in t3C concentration and its effect on environmental reconstruction using 13C/12C ratios in animal bones. In Price, T. D. (ed.), *The Chemistry of Prehistoric Human Bone,* Cambridge University Press, Cambridge, pp. 105-125.

18. Fischer, A., Olsen, J., Richards, M., Heinemeier, J., Sveinbjörnsdóttir, A. E., Bennike, P. 2007. Coast-inland mobility and diet in the Danish Mesolithic and Neolithic: evidence from stable isotope values of humans and dogs. *Journal of Archaeological Science* 34, 2125-2150.

19. Szewciw, L. J., de Kerckhove, D. G., Grime, G. W., Fudge, D. S. 2010. Calcification provides mechanical reinforcement to whale baleen alpha-keratin, *Proceedings of the Royal Society B: Biological Sciences* 277(1694), 2597-605.

20. Smith, M. E. K., Ososky, J. J., Hunt, K. E., Cioffi, W. R., Read, A. J., Friedlaender, A. S., McCarthy, M., Fleming, A. H. 2024. Historical baleen plates indicate that once abundant Antarctic blue and fin whales demonstrated distinct migratory and foraging strategies. *Ecology and Evolution* 14, e11376.

21. Schell, D. M., Saupe, S. M., Haubenstock, N. 1989. Bowhead whale (*Balaenoptera mysticetus*) growth and feeding as estimated by *δ*13C techniques. *Marine Biology* 103, 433–443.

22. Keeling, C. D. 1979. The Suess effect: ^{13}Carbon-^{14}Carbon interrelations. *Environment International* 2(4–6), 229–300.

23. Clark, C. T., Cape, M. R., Shapley, M. D., Mueter, F. J., Finney, B. P., Misarti, N. 2020. SuessR: Regional corrections for the effects of anthropogenic CO2 on δ13C data from marine organisms. *Methods in Ecology and Evolution* 2021:12, 1508–1520.

24. Bas, M., García, N. A., Crespo, E. A., Cardona, L. 2019. Intraskeletal variability in stable isotope ratios of C and N among pinnipeds and cetaceans. *Marine Mammal Science* 36, 375–385.

25. Das, K., Holleville, O., Ryan, C., Berrow, S., Gilles, A., Ody, D., Michel, L. N. 2017. Isotopic niches of fin whales from the Mediterranean Sea and the Celtic Sea (North Atlantic). *Marine Environmental Research* 127, 75e83.

26. Smith, K. J., Sparks, J. P., Timmons, Z. L., Peterson, M. J. 2020. Cetacean Skeletons Demonstrate Ecologically Relevant Variation in Intraskeletal Stable Isotopic Values. *Frontiers in Marine Science* 7, 388.

27. Lee, S. H., Schell, D. M., McDonald, T. L., Richardson, W. J. 2005. Regional and seasonal feeding by bowhead whales *Balaena mysticetus* as indicated by stable isotope ratios. *Marine Ecology Progress Series* 285, 271–287.

28. Peterson, B. J., Fry, B. 1987. Stable isotopes in ecosystem studies. *Annual Review of Ecology, Evolution, and Systematics* 18, 293–320.

29. Jory, C., Lesage, V., Leclerc, A., Giard, J., Iverson, S., Bérubé, M., Michaud, R., Nozais, C. 2021. Individual and population dietary specialization decline in fin whales during a period of ecosystem shift. *Scientific Reports* 11:17181.

30. Aguilar, A., Giménez, J., Gómez-Campos, E., Cardona, L., Borrell, A. 2014. d15N value does not reflect fasting in Mysticetes. *PLoS ONE* 9(3), e92288.

31. Rita, D., Borrell, A., Wodarg, D., Víkingsson, G., García-Vernet, R., Aguilar, A., Loick-Wilde, N. 2023. Amino acid-specific nitrogen stable isotope analysis reveals the trophic behavior of Icelandic fin whales in winter and suggests variable feeding strategies. *Marine Mammal Science* 1–14.

32. Magozzi, S., Yool, A., Vander Zanden, H. B., Wunder, M. B., Trueman, C. N. 2024. Using ocean models to predict spatial and temporal variation in marine carbon isotopes. *Ecosphere* 8(5), e01763.

33. Silva, M. A., Prieto, R., Jonsen, I., Baumgartner, M. F., Santos, R. S. 2013. North Atlantic Blue and Fin Whales Suspend Their Spring Migration to Forage in Middle Latitudes: Building up Energy Reserves for the Journey? *PLoS ONE* 8(10), e76507.

34. Wilson, D. E., Mittermeier, R. A. 2014. *Handbook of the Mammals of the World: Volume 4, Sea Mammals*, Barcelona, Lynx Edicions, 614 p.

35. Borrell, A., Velásquez Vacca, A., Pinela, A. M., Kinze, C., Lockyer, C. H., Vighi, M., Aguilar, A. 2013. Stable Isotopes Provide Insight into Population Structure and Segregation in Eastern North Atlantic Sperm Whales. *PLoS ONE* 8(12), e82398.

36. Pontbriand, T., Davoren, G., Fortune, S., Pomerleau, C., Young, B., Ferguson, S. 2023. Differences in individual bowhead whale *Balaena mysticetus* habitat use, foraging dive depth and diet during the peak feeding season. *Marine Ecology Progress Series* 706, 109–123.
