# Supplementary material for: Late Paleolithic whale bone tools reveal human and whale ecology in the Bay of Biscay
Source: Nat Commun. 2025 May 27;16:4646. doi: 10.1038/s41467-025-59486-8 (PMC12117114; doi:10.1038/s41467-025-59486-8)
Supplement: Supplementary file 2 — Description of Additional Supplementary Files [file 41467_2025_59486_MOESM2_ESM.pdf]

Description of Additional Supplementary Files

Supplementary Data 1:
Extrinsic, intrinsic and analytical information for all the elements studied.

Supplementary Data 2:
Updated inventory of the worked objects made of whale bone found in Magdalenian sites.

Supplementary Data 3:
Collagen peptide markers used to make ZooMS identifications of study samples.

Supplementary Data 4:
Table file (3 sheets) comparing stable isotope datasets from the Paleolithic whales with modern whales.

Supplementary Code 1:
Code used in OxCal 4.4 for the calculation of the calibrated and reservoir corrected ^{14}C ages of the whale samples.
