# Supplementary material for: Late Paleolithic whale bone tools reveal human and whale ecology in the Bay of Biscay
Source: Nat Commun. 2025 May 27;16:4646. doi: 10.1038/s41467-025-59486-8 (PMC12117114; doi:10.1038/s41467-025-59486-8)
Supplement: Supplementary file 8 — Reporting Summary [file 41467_2025_59486_MOESM8_ESM.pdf]

nature portfolio

Corresponding author(s): Jean-Marc Pétillon

Last updated by author(s): Mar 31, 2025

Reporting Summary

Nature Portfolio wishes to improve the reproducibility of the work that we publish. This form provides structure for consistency and transparency in reporting. For further information on Nature Portfolio policies, see our Editorial Policies and the Editorial Policy Checklist.

Statistics

For all statistical analyses, confirm that the following items are present in the figure legend, table legend, main text, or Methods section.

n/a	Confirmed	
☐	☒	The exact sample size (*n*) for each experimental group/condition, given as a discrete number and unit of measurement
☐	☒	A statement on whether measurements were taken from distinct samples or whether the same sample was measured repeatedly
☐	☒	The statistical test(s) used AND whether they are one- or two-sided<br>*Only common tests should be described solely by name; describe more complex techniques in the Methods section.*
☒	☐	A description of all covariates tested
☒	☐	A description of any assumptions or corrections, such as tests of normality and adjustment for multiple comparisons
☒	☐	A full description of the statistical parameters including central tendency (e.g. means) or other basic estimates (e.g. regression coefficient) AND variation (e.g. standard deviation) or associated estimates of uncertainty (e.g. confidence intervals)
☒	☐	For null hypothesis testing, the test statistic (e.g. *F*, *t*, *r*) with confidence intervals, effect sizes, degrees of freedom and *P* value noted<br>*Give P values as exact values whenever suitable.*
☒	☐	For Bayesian analysis, information on the choice of priors and Markov chain Monte Carlo settings
☒	☐	For hierarchical and complex designs, identification of the appropriate level for tests and full reporting of outcomes
☒	☐	Estimates of effect sizes (e.g. Cohen's *d*, Pearson's *r*), indicating how they were calculated

Our web collection on statistics for biologists contains articles on many of the points above.

Software and code

Policy information about availability of computer code

Data collection: All the intrinsic and extrinsic information on the samples studied, as well as radiocarbon data and ZooMS identifications, has been collected and presented on Excel spreadsheets (Supplementary Data 1 through 4).

Data analysis: The open access software OxCal was used to calibrate the radiocarbon ages and the correct credits for using this software are given (Bronk Ramsey 2009). The code itself has been included as a Supplementary Code file.

For manuscripts utilizing custom algorithms or software that are central to the research but not yet described in published literature, software must be made available to editors and reviewers. We strongly encourage code deposition in a community repository (e.g. GitHub). See the Nature Portfolio guidelines for submitting code & software for further information.

Data

Policy information about availability of data

All manuscripts must include a data availability statement. This statement should provide the following information, where applicable:

- Accession codes, unique identifiers, or web links for publicly available datasets
- A description of any restrictions on data availability
- For clinical datasets or third party data, please ensure that the statement adheres to our policy

A data availability statement is included in the manuscript. All data generated or analysed during this study are included in this published article or in public data repositories. There is no restriction on the data availability. All radiocarbon related information (Lab numbers, sample starting masses, collagen yields, AMS

April 2023

numbers, conventional 14C ages, calibrated 14C ages, stable isotope ratios, atomic C/N ratios) is available in the Supplementary Data 1 file. Collagen peptide mass spectra have been uploaded to a public depository: https://doi.org/10.34847/nkl.dbbfl6fw

Research involving human participants, their data, or biological material

Policy information about studies with human participants or human data. See also policy information about sex, gender (identity/presentation), and sexual orientation and race, ethnicity and racism.

Reporting on sex and gender	N/A
Reporting on race, ethnicity, or other socially relevant groupings	N/A
Population characteristics	N/A
Recruitment	N/A
Ethics oversight	N/A

Note that full information on the approval of the study protocol must also be provided in the manuscript.

Field-specific reporting

Please select the one below that is the best fit for your research. If you are not sure, read the appropriate sections before making your selection.

☐ Life sciences ☐ Behavioural & social sciences ☒ Ecological, evolutionary & environmental sciences

For a reference copy of the document with all sections, see nature.com/documents/nr-reporting-summary-flat.pdf

Ecological, evolutionary & environmental sciences study design

All studies must disclose on these points even when the disclosure is negative.

Study description	We analyzed worked bone objects, and unworked bone fragments, found in Late Upper Paleolithic sites (Magdalenian culture) from southwest France and northern Spain. These specimens had been visually identified as being made of whale bone in previous studies (Pétillon 2013, Castaños 2014, Lefebvre et al. 2021). The specimens were sampled for radiocarbon dating, stable isotope analysis, and species identification with ZooMS. The results provide the first assessment of the whale species present in the Bay of Biscay at that time, their diet and chronology.
Research sample	Before this study, 165 worked objects visually identified as made of whale bone had been reported from 25 Magdalenian sites. The reassessment of ancient collections as a part of this study, in combination with recent excavations, yielded 20 additional objects, bringing the total to 185 objects from 31 Magdalenian sites (Supplementary Data 2). In order to maintain the morphological integrity of the worked bone assemblage, a destructive ZooMS approach was applied to 83 of the 185 objects (i.e., 45%), with samples selected to ensure a representative selection of sites (n = 26) and of the objects' typology. In addition, at Santa Catalina, 41 unworked bone fragments had previously been attributed to cetaceans on a visual basis. In this study, the bone assemblage of Santa Catalina was reassessed and sampled to include all fragments that could potentially be whale bone, even if the morphological identification remained somewhat uncertain. A total of 90 unworked bone fragments from Santa Catalina were subjected to ZooMS analysis. Half of the elements whose identification as whale bone was confirmed by ZooMS were subject to additional sampling for radiocarbon dating and stable isotope analysis: 37 of the 71 worked objects (52%), and 31 of the 60 bones from Santa Catalina (52%).
Sampling strategy	Minimally-invasive ZooMS analysis was tested on a subset of the samples, but proved to be unsuccessful. Depending on the shape of each element, destructive sampling was done either: by cutting a piece from one of the extremities of the bone; by drilling with a pocket drill; or by coring using homemade diamond-coated core drills. Before any sampling, photogrammetry was used to produce a 3D model of each worked object and each unworked bone fragment in its integrity. Details are provided in the Methods section under the heading "Sample selection, photogrammetry and sampling".
Data collection	Collagen extraction, radiocarbon and stable isotope data have been collected by L. van der Sluis. This information has also been stored on a shared cloud hosted by the National museum of natural history Paris, which was set up by A. Zazzo and O. Tombret. For ZooMS analysis, collagen extraction and mass spectral analysis was conducted by Krista McGrath, and has been uploaded to a public repository.
Timing and spatial scale	Sampling dates were constrained by the acquisition of sampling permits from 22 different institutions (see below), the availability of museum curators, and the restrictions of the covid pandemic. Sampling work was carried out in 21 discrete sessions between May 2017 and April 2023. The sampling date for each object is mentioned in Supplementary Data 1 file. The objects sampled are from 26 archeological sites in the Asturias (ES), Cantabria (ES), Euskadi (ES), Nouvelle-Aquitaine (FR) and Occitanie (FR) regions, plus one object from Rhineland (D).
Data exclusions	Within this project we encountered a number of samples that had been contaminated with bone glue; these samples are discussed in van der Sluis et al., 2023 (Scientific Reports 13: 22119). The ZooMS identification of these samples is included in the data of this

	current submission, although we excluded the radiocarbon data of these samples, considering that the 14C ages are unreliable due to the bone glue contamination.
Reproducibility	The produced conventional radiocarbon ages can be calibrated again at any point using the freely available calibration software. The most up to date calibration curve has been used (Marine20). The calibrated ages could change in the future after another update of the calibration curve. However, the produced conventional radiocarbon ages can be used for this, they do not change. For the ZooMS analysis, the mass spectra have been uploaded to a public repository and can be re-analyzed at any point.
Randomization	The concept of blinding is not practised in the fields of radiocarbon dating and ZooMS analysis. For radiocarbon dating however, practises of quality assurance consist of analysing standard samples (known-age samples, oxalic acid and bone blanks) alongside unknown samples. A bone blank is used to define the background correction, which is then subtracted from all samples, and this can then be checked against a standard of known age, such as VIRI samples.
Blinding	See "Randomization" above.

Did the study involve field work? ☐ Yes ☒ No

Reporting for specific materials, systems and methods

We require information from authors about some types of materials, experimental systems and methods used in many studies. Here, indicate whether each material, system or method listed is relevant to your study. If you are not sure if a list item applies to your research, read the appropriate section before selecting a response.

Materials & experimental systems

n/a	Involved in the study
☒	☐ Antibodies
☒	☐ Eukaryotic cell lines
☐	☒ Palaeontology and archaeology
☒	☐ Animals and other organisms
☒	☐ Clinical data
☒	☐ Dual use research of concern
☒	☐ Plants

Methods

n/a	Involved in the study
☒	☐ ChIP-seq
☒	☐ Flow cytometry
☒	☐ MRI-based neuroimaging

Palaeontology and Archaeology

Specimen provenance	The specimens analyzed in this study are permanently stored in 22 institutions: 19 public museums (10 in France, 7 in Spain, 1 in Germany and 1 in the UK) and 3 curatorial repositories under the responsibility of the French ministry of Culture. The detailed list of storage places, object by object, appears in the Supplementary Data 1 file and is mentioned in the acknowledgements. Accession and sampling permits were obtained in each case from the relevant authority (museum curator, regional/local heritage authority, regional archeological service), with advice from the excavation directors when applicable, from 2017 to 2021. Sampling was carried out from 2017 to 2023 by J.-M. Pétillon and A. Lefebvre, either at the museums or at the TRACES laboratory (Toulouse, France) when loaning was permitted.
Specimen deposition	By the end of the study, all the objects sampled had been returned to their original storage institution. They are available for further analysis and sampling requests. All the material sampled from the objects has been used (consumed) in the analysis.
Dating methods	New radiocarbon dates were produced during this project on archaeological bone objects and all the information related to the chemistry pretreatment, combustion, graphitisation and AMS measurement are reported in the Methods section under the heading "Radiocarbon dating" and in the Supplementary Data 1 file.

☒ Tick this box to confirm that the raw and calibrated dates are available in the paper or in Supplementary Information.

Ethics oversight	Accession and sampling permits were obtained for each archeological collection from the relevant authority (museum curator, regional/local heritage authority, regional archeological service), with advice from the excavation directors when applicable. Sampling protocols were negociated and approved by said authority in each case. No other ethical approval was required.

Note that full information on the approval of the study protocol must also be provided in the manuscript.

Plants

Seed stocks	N/A
Novel plant genotypes	N/A
Authentication	N/A
